# Particle-attached bacteria act as gatekeepers in the decomposition of complex phytoplankton polysaccharides

**DOI:** 10.1186/s40168-024-01757-5

**Published:** 2024-02-20

**Authors:** Feng-Qing Wang, Daniel Bartosik, Chandni Sidhu, Robin Siebers, De-Chen Lu, Anke Trautwein-Schult, Dörte Becher, Bruno Huettel, Johannes Rick, Inga V. Kirstein, Karen H. Wiltshire, Thomas Schweder, Bernhard M. Fuchs, Mia M. Bengtsson, Hanno Teeling, Rudolf I. Amann

**Affiliations:** 1 Max Planck Institute for Marine Microbiology, Celsiusstraße 1, 28359 Bremen, Germany; 2 Institute of Pharmacy, University of Greifswald, Felix-Hausdorff-Straße 3, 17489 Greifswald, Germany; 3 Institute of Marine Biotechnology, Walther-Rathenau-Straße 49a, 17489 Greifswald, Germany; 4 Institute of Microbiology, University of Greifswald, Felix-Hausdorff-Straße 8, 17489 Greifswald, Germany; 5 Marine College, Shandong University, Weihai, 264209 China; 6 Max Planck Genome Centre Cologne, Carl von Linné-Weg 10, 50829 Cologne, Germany; 7 Alfred Wegener Institute for Polar and Marine Research, Biologische Anstalt Helgoland, Helgoland, 27483 Germany

**Keywords:** Algal bloom, Algal polysaccharide, Bacterioplankton, *Bacteroidota*, Carbohydrate-active enzyme, Carbon budget, Carbon cycle, Free-living bacteria, Helgoland Roads LTER, Marine microbes, Particle-attached bacteria, Particulate organic matter, Polysaccharide utilization locus

## Abstract

**Background:**

Marine microalgae (phytoplankton) mediate almost half of the worldwide photosynthetic carbon dioxide fixation and therefore play a pivotal role in global carbon cycling, most prominently during massive phytoplankton blooms. Phytoplankton biomass consists of considerable proportions of polysaccharides, substantial parts of which are rapidly remineralized by heterotrophic bacteria. We analyzed the diversity, activity, and functional potential of such polysaccharide-degrading bacteria in different size fractions during a diverse spring phytoplankton bloom at Helgoland Roads (southern North Sea) at high temporal resolution using microscopic, physicochemical, biodiversity, metagenome, and metaproteome analyses.

**Results:**

Prominent active 0.2–3 µm free-living clades comprised *Aurantivirga*, “Formosa”, *Cd*. Prosiliicoccus, NS4, NS5, *Amylibacter*, *Planktomarina*, SAR11 Ia, SAR92, and SAR86, whereas BD1-7, *Stappiaceae*, *Nitrincolaceae*, *Methylophagaceae*, *Sulfitobacter*, NS9, *Polaribacter*, *Lentimonas*, CL500-3, *Algibacter*, and *Glaciecola* dominated 3–10 µm and > 10 µm particles. Particle-attached bacteria were more diverse and exhibited more dynamic adaptive shifts over time in terms of taxonomic composition and repertoires of encoded polysaccharide-targeting enzymes. In total, 305 species-level metagenome-assembled genomes were obtained, including 152 particle-attached bacteria, 100 of which were novel for the sampling site with 76 representing new species. Compared to free-living bacteria, they featured on average larger metagenome-assembled genomes with higher proportions of polysaccharide utilization loci. The latter were predicted to target a broader spectrum of polysaccharide substrates, ranging from readily soluble, simple structured storage polysaccharides (e.g., laminarin, α-glucans) to less soluble, complex structural, or secreted polysaccharides (e.g., xylans, cellulose, pectins). In particular, the potential to target poorly soluble or complex polysaccharides was more widespread among abundant and active particle-attached bacteria.

**Conclusions:**

Particle-attached bacteria represented only 1% of all bloom-associated bacteria, yet our data suggest that many abundant active clades played a pivotal gatekeeping role in the solubilization and subsequent degradation of numerous important classes of algal glycans. The high diversity of polysaccharide niches among the most active particle-attached clades therefore is a determining factor for the proportion of algal polysaccharides that can be rapidly remineralized during generally short-lived phytoplankton bloom events.

Video Abstract

**Supplementary Information:**

The online version contains supplementary material available at 10.1186/s40168-024-01757-5.

## Background

Global photosynthetic net primary production (NPP) amounts to an estimated 104.9 gigatons of carbon per year [1]. Almost half of this is allotted to algae, in particular, to the small unicellular planktonic algae (phytoplankton) that dominate the world’s oceans [2]. Diatoms (*Bacillariophyta*) represent the most prominent phytoplankton group, in particular, in polar and upwelling regions, and have been estimated to fix up to 20 gigatons of carbon annually [3]. It has been suggested that the silicate shells (frustules) of diatoms provide a competitive advantage over other phytoplankton by allowing them to save energy for cytoskeleton maintenance [4]. Further abundant and globally distributed phytoplankton taxa include photosynthetic dinoflagellates and haptophytes (*Haptophyta*), such as coccolithophorids. For the haptophyte genus *Phaeocystis*, it has been shown that their small cells with high surface-to-volume ratios can outcompete diatom productivity under certain conditions [5]. *Phaeocystis* often dominates spring and summer blooms after diatoms have peaked in the coastal North Sea [6], where they can account for up to 65% of the annual primary production [7].

Primary production by marine phytoplankton is not constant but culminates during phytoplankton blooms. Such blooms can be massive, yet they are usually short-lived. Bloom termination is often initiated by nutrient depletion and can be amplified by a number of factors, such as self-shading, grazing (e.g., by copepods), and various infections, e.g., by viruses, algicidal bacteria, parasitic peronosporomycetes (oomycetes), dinoflagellates, and marine fungi [8–11]. Also, the coagulation of algae and increased sinking of the formed particles due to reduced buoyancy can play a role [12].

During phytoplankton blooms, copious amounts of algal organic matter are released as dissolved or particulate organic matter (DOM, POM). Most of this is rapidly remineralized by heterotrophic bacteria and zooplankton, but the exact proportions are a matter of debate. It has been estimated that 62% of the daily phytoplankton production is on average consumed by small zooplankton [13] (reviewed in [14]). Zooplankton sloppy feeding and excretion in turn increase the DOM and POM pools available to bacteria [15], and measurements of bacterial respiration rates have suggested that bacteria remineralize 70–92% of the POM within the mesopelagic zone (− 200 to − 1000 m) [16]. Only about 1–3% of biological net primary production reaches bathypelagic depths (below − 1000 m) [17] via the so-called biological pump, where it can be sequestered for longer periods of time. According to recent estimates, about 10 gigatons of carbon are exported to the deep sea annually, including 1.3 gigatons by the biological pump, 15% of which is phytodetritus [18].

About 95 to > 99% of the epipelagic marine bacteria typically consist of DOM-decomposing free-living (FL) planktonic bacteria (bacterioplankton) and the remainder of POM-decomposing particle-attached (PA) bacteria [19–21]. However, high particle abundances can elevate proportions of PA bacteria, at times possibly even above those of FL bacteria [19]. FL bacteria have been estimated to mediate 53% of the DOM and PA bacteria 50% of the POM fluxes [16]. Likewise, PA bacteria have been shown to exhibit higher per-cell activities (e.g., [20]) and higher proportions of hydrolytic enzymes [22, 23]. However, currently, we have only a poor understanding of the factors that determine the fractions of the organic matter that are remineralized by FL bacteria, PA bacteria, and the fractions that either feed the pool of recalcitrant DOM or sink out to the sea floor.

Depending on the developmental stage and physiological condition, up to 75% [24] or even more [25] of the dry weight of algae can consist of various polysaccharides, e.g., as intracellular stores of biochemical energy and as cell matrix and cell wall components. Many of these polysaccharides have no counterparts in terrestrial plants, in particular, those that are anionic, e.g., due to sulfation. The dominating polysaccharides in marine macroalgae (seaweeds) are well known, such as laminarins, fucoidans, cellulose, and alginates in brown algae (*Phaeophyta*); cellulose, xylans, and ulvans in green algae (*Chlorophyta*); and agars, carrageenans, and galactans (including porphyran and furcellaran) in red algae (*Rhodophyta*). Less is known about microalgal polysaccharides. Brown macroalgae and other stramenopiles, including diatom and raphidophyte phytoplankters, contain laminarin as a store of photoassimilated biochemical energy [26]. Laminarin, which is also used by haptophyte phytoplankters, is a water-soluble, structurally simple β-1,3-linked helical homopolymer of glucose with occasional β-1,6-branches that typically consist of 20 to 30 monomers [27]. The dry weight of diatoms can consist of up to 35% of laminarin during exponential growth, and even up to 80% has been reported for the stationary phase [28]. Laminarin is therefore one of the most abundant polysaccharides on Earth [29].

The polysaccharides that are encrusted in the siliceous diatom frustules are more heterogeneous, and little is known about their structures. Studies of *Phaeodactylum tricornutum* have identified a sulfated glucuronomannan as a major cell wall component that might be widespread in diatoms [30]. However, monosaccharides other than mannose and glucuronic acid have been identified in frustules, including fucose, galactose, glucose, xylose, rhamnose, and arabinose [31]. Compositions depend on diatom species and physiological state, which would indicate a huge diversity in corresponding structures. Considering the prevalence of diatoms, these polysaccharides are produced in large quantities and play a non-negligible role in global carbon cycling.

Many microalgae also exudate polysaccharide-rich extracellular polymeric substances (EPS). EPS have many functions, e.g., providing a nutritious matrix to attract beneficial bacteria, particularly in the immediate algal phycosphere. EPS also increase cell surface adhesiveness and thereby promote algae aggregation and flocculation [32–34]. Likewise, a portion of the EPS itself can coagulate into more dense transparent extracellular particles (TEP). The amount of EPS that algae produce depends on many factors. It tends to increase when nutrients become limiting, which is commonly interpreted as a mechanism to dispose excess carbon [35] as a substitute for adaptive photosynthesis downregulation. Not much is known about EPS composition, which may vary depending on algal species and physiological conditions, but most EPS seem to contain high proportions of sulfated polysaccharides [36].

Due to the inherent chemical heterogeneity and structural complexity, no bacterium can harbor the genes required to decompose all algal polysaccharides. Instead, bacteria specialize in subsets, which is why the remineralization of algal polysaccharides is a collective endeavor of polysaccharide-degrading bacteria with distinct substrate niches. Genes that code for the polysaccharide degradation machinery in bacterial genomes are often co-located as operons or regulons. Such polysaccharide utilization loci (sg. polysaccharide utilization locus (PUL)) are particularly prominent in the genomes of polysaccharide-degrading *Bacteroidota*, where they typically comprise a *susCD* gene tandem that codes for a SusD-like substrate-binding and for a SusC-like channel protein of a TonB-dependent transporter (TBDT) [37]. These are accompanied by genes coding for degradative carbohydrate-active enzymes (CAZymes), namely glycoside hydrolases (GHs), carbohydrate esterases (CEs), polysaccharide lyases (PLs), and by accessory genes coding for, e.g., surface glycan-binding proteins (e.g., [38]), sulfatases, ABC transporters, and other associated functions. PUL lengths depend on the target substrate and can vary considerably. While a typical laminarin PUL consists of around 20 genes (e.g., [39]), PUL-rich loci can also encompass close to 100 genes (e.g., [40]).

We have analyzed the microbial response of bacteria to spring phytoplankton blooms in a series of studies at the long-term ecological research (LTER) site “Kabeltonne” off Helgoland Island in the southern North Sea [39, 41–44], in which we focused on the response of FL (0.2–3 µm) bacteria and their associated polysaccharide niches. Recently, we could exemplarily show that abundant bloom-associated FL bacterioplankton clades preferentially consume water-soluble, low-complexity storage polysaccharides such as laminarin and α-glucans, which therefore exert a strong community structuring effect [39]. Also, other polysaccharides, such as alginate or mannose-containing polysaccharides, play a role, albeit in lower quantities [39]. An unknown proportion of the dissolved polysaccharides originate from POM that has been solubilized by PA bacteria and diffused away before uptake. However, so far, little is known about the involved polysaccharide-degrading PA bacteria and their connection to FL bacteria. A recent comparative study of FL and PA metagenome-assembled genomes (MAGs) from different water depths in the North Pacific Subtropical Gyre has shown that PA bacteria are characterized by higher predicted growth efficiencies and, on average, larger genomes with higher proportions of genes for peptidases, CAZymes, secretion, sensing and motility [23].

In this study, we investigated a diverse spring phytoplankton bloom that took place in 2018 off Helgoland Roads at high temporal resolution (51 sampling dates over a 90-day period). We aimed to disentangle the roles of PA bacteria in comparison with FL bacteria with respect to their potential to degrade phytoplankton-derived polysaccharides. We collected microscopic algal biodiversity and biovolume data, eukaryote 18S rRNA gene amplicon data, and 16S rRNA gene amplicon data of bacterial communities from FL (0.2–3 µm), PA3 (3–10 µm), and PA10 (> 10 µm) filter fractions together with a broad range of physicochemical data. In addition, we performed metagenomics of FL (18 samples), PA3 (16 samples), and PA10 (8 samples) bacterial communities, reconstructed MAGs of abundant key players, and compared their polysaccharide degradation potentials. These data were complemented by metaproteomes from 10 selected time points during the bloom to link bacterial protein to the decomposition of algal glycans.

## Results

### The 2018 Helgoland spring phytoplankton bloom was diverse and polyphasic

**Fig. 1:**
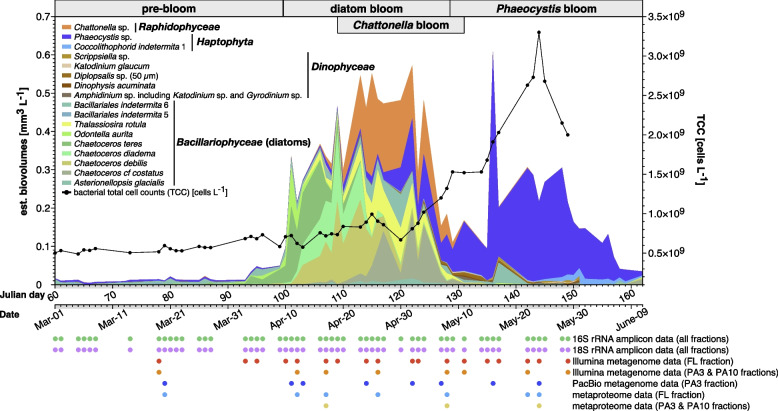
2018 spring phytoplankton bloom at Helgoland Roads and associated datasets. Estimated biovolumes of abundant phytoplankton taxa (stacked colored areas) as assessed by microscopy and of total cell counts (TCC) of DAPI (4′,6-diamidino-2-phenylindole)-stained bacteria (black line). Complete data was sampled until the end of May. Additional data for June is shown to visualize the end of the bloom but does not include TCC and *Dinophyceae* biovolume information. The core sampling period from March 1 until May 31 is indicated by a gray area on the abscissa. Additional data including abundances of non-photosynthetic plankters are provided in Additional file 2: Fig. S1 and Additional file 3. Associated omics sampling dates are indicated by colored circles at the bottom

Based on microscopically determined phytoplankton taxa, corresponding biovolume estimates (< 0.1 to 1.71 mm^3^ L^−1^, Additional file 1: Table S1), and chlorophyll *a* measurements (~ 2 to 33.8 units, Additional file 1: Table S1), the 2018 Helgoland spring bloom consisted of a pre-bloom phase dominated by lowly abundant diatoms and *Phaeocystis* sp. haptophytes (March 1 to April 9), a diatom-dominated phase (April 10 to May 08) largely overlapping with a notable bloom of *Chattonella* raphidophytes (April 19 to May 11), and a late phase dominated by *Phaeocystis* sp. haptophytes and few *Dinophyceae* (May 09 to May 31) (Fig. 1).

Diatoms comprised various *Chaetoceros* species and *Thalassiosira rotula*. After they went into decline, *Phaeocystis* sp. and *Dinophyceae* numbers increased, with *Phaeocystis* sp. becoming dominant until the first wave of blooming algae ended about 1 week into June. A remarkable correlation was obtained between chlorophyll *a* measurements and estimated biovolumes of photosynthetic plankters (Additional file 2: Fig. S1A). Additional non-photosynthetic plankters comprised in particular dinoflagellates, e.g., *Noctiluca scintillans*. The latter was detected at the end of May and, despite being low in numbers, dominated the biovolume of unicellular eukaryotic plankters due to large cell sizes (Additional file 1: Table S1, Additional file 2: Fig. S1B).

Analysis of the 15 most abundant 18S rRNA gene amplicon sequence variants (ASVs) largely supported microscopic observations (Additional file 2: Fig. S2). For example, the *Chaetoceros* bloom was detected in the PA10, and the *Phaeocystis* and *Noctiluca* blooms in the PA3 fractions (*Phaeocystis* cells are small, and *Noctiluca* cells are fragile and thus broke during filtration). One inconsistency was that *Chattonella* could not be detected, likely because their particularly fragile, large, wall-less cells disintegrated during filtration. In addition, 18S rRNA ASV data revealed a noteworthy peak of *Cryothecomonas* nanoflagellates towards the end of the diatom bloom.

We focused on the period from March 1 to May 31. FL bacterial total cell counts (TCC) increased continuously from 0.5 × 10^9^ L^−1^ on March 1 to a peak of 3.3 × 10^9^ L^−1^ on May 24 (Fig. 1). This increase was gradual during the pre- and main bloom phases and progressed more rapidly after diatoms peaked at the end of April. Likewise, flagellate numbers increased throughout the bloom, ranging from 2.4 × 10^6^ L^−1^ on March 1 to 1.2 × 10^7^ L^−1^ on May 16 (Additional file 2: Fig. S3A). Flagellate numbers correlated well with Chl *a* and biovolume estimates, indicating that flagellates not only preyed on bacteria but also on microalgae. The remaining zooplankton was dominated by various copepod species with undulating abundances over time that showed no clear correlation to phytoplankton data, possibly due to vertical migration in and out of the sampled surface water (Additional file 1: Table S2).

### An influx of nutrient-rich coastal water triggered the onset of the bloom

Physicochemical data indicated an incursion of nutrient-rich coastal waters at the onset of the diatom bloom, as on April 10 nitrate concentrations spiked to 19.0 µM, silicate concentrations spiked to 10.7 µM, and salinity decreased from 33.8 to 32.6 (Additional file 2: Fig. S3B-D). A second influx likely occurred from May 22 to 29 and was accompanied by an increase in silicate concentrations from 1.0 to 4.0 µM and a drop of salinity to 31.7. A spike in phosphate concentrations from 0 to 0.7 µM was also detected during this period (Additional file 2: Fig. S3E).

Wind directional data (Additional file 1: Table S3) supported these influx events, since northeasterly to easterly winds dominated from April 9 to 14 and from May 23 to 30 (Additional file 2: Fig. S4; see [39] for details). Additional rain and sunshine data are provided in Additional file 1: Table S4.

### FL and PA bacterial communities exhibited distinct diversities and compositional shifts over the bloom’s progression

**Fig. 2:**
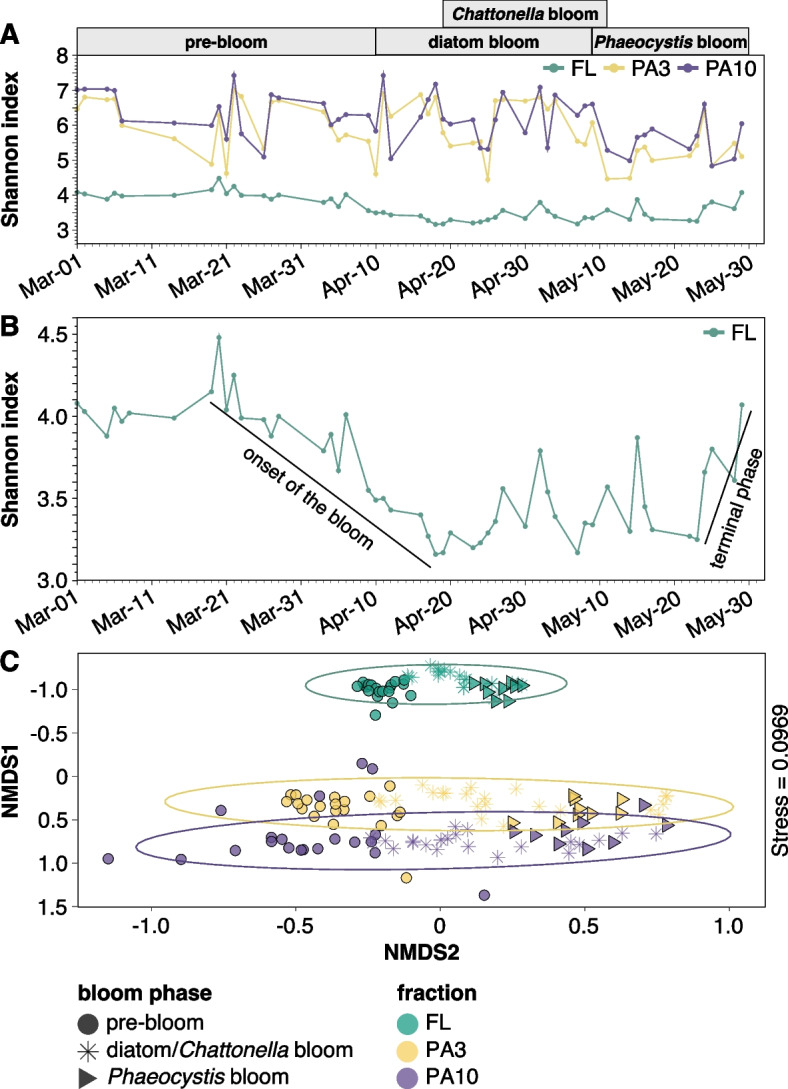
Diversity as assessed by 16S rRNA gene amplicon data. **A** Shannon index of the sampled bacterial communities over time. Colors correspond to sample fractions (FL, 0.2–3 µm; PA3, 3–10 µm; PA10, > 10 µm). **B** Shannon index of the FL fraction over time. **C** Non-metric multidimensional scaling (NMDS) analysis of the bacterial community of all three fractions. Colors correspond to sample fractions and shapes to bloom phases

16S rRNA gene amplicon sequencing of 153 samples from FL and PA fractions yielded 24,356 unique ASVs (Additional file 1: Table S5). Good’s coverage (a measure for the proportion of singletons) indicated that this was adequate to capture basically all of the diversity of the FL communities (avg. coverage, ~ 1.0) and most of the diversities of both PA fractions (avg. coverages, 0.96 and 0.91, respectively) (Additional file 2: Fig. S5A). FL bacterial communities had significantly lower alpha diversity indices (Chao1, Simpson’s, Shannon) than PA3 and PA10 communities (ANOVA, *p* < 0.001), whereas the difference between both PA communities was less pronounced (Additional file 2: Fig. S5B-D). Shannon indices exhibited distinct patterns over time (Fig. 2A, B), with a high correlation of PA3 and PA10 samples (*p* < 0.0001, Fig. 2A). FL Shannon indices were highest prior to the pre- and early diatom-bloom, decreased and stayed low during the main diatom and early *Phaeocystis* bloom phases, and finally increased again towards the bloom’s end in the late *Phaeocystis* bloom. No such trend was observed for PA communities (Fig. 2A, B).

NMDS analyses based on weighted UniFrac distances corroborated that PA3 and PA10 communities were more alike and FL communities more distinct. Pre-bloom communities grouped well and were distinct from main and late-bloom communities (Fig. 2C, left to right). While these differences were less pronounced between the two main bloom phases, they were still detectable. The average distances between the pre-bloom and the main bloom stages were also smaller in the FL than in both PA fractions (Fig. 2C), indicating that the bloom caused a more profound community change in both PA fractions.

### Distinct bloom phases selected for distinct genera in all size fractions

**Fig. 3:**
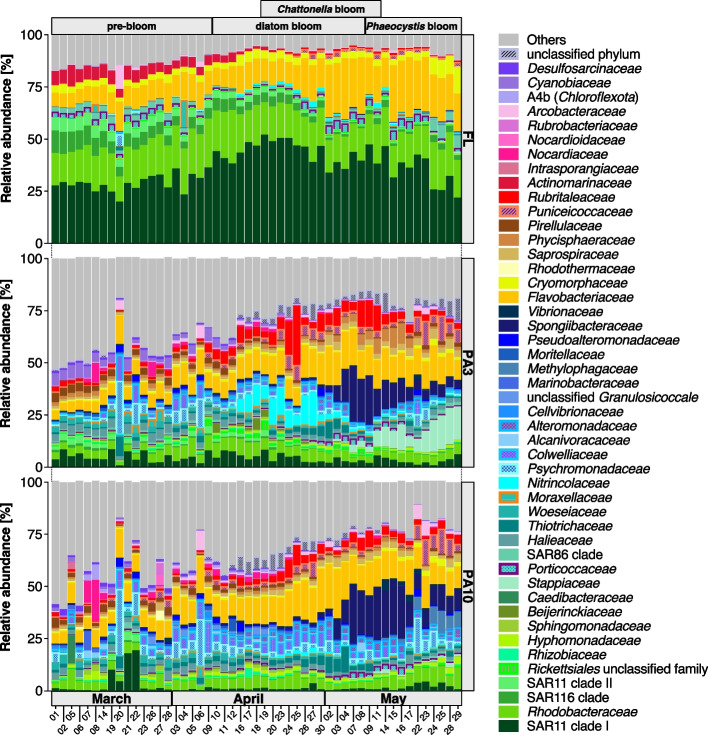
Compositional differences among fractions as assessed by 16S rRNA gene amplicon data. Relative abundances of families are shown that belong to the topmost five abundant in at least two samples. The remaining families are subsumed as “Others.” Details are provided in Additional file 1: Table S5

Both, FL and PA communities showed clear temporal successions of distinct bacterial clades. PA communities, however, were not only more diverse but also dominated by different taxa and exhibited more dynamic compositional shifts (Fig. 3, Additional file 1: Table S5). While FL communities were dominated by *Alphaproteobacteria*, proportions were lower within PA3 and PA10 communities (Additional file 2: Fig. S6A). In contrast, *Gammaproteobacteria* exhibited particularly high relative abundances in PA3 and PA10 communities but less so in FL communities. Likewise, *Verrucomicrobiota* and *Planctomycetota* exhibited higher relative abundances in PA3 and PA10 than in FL communities, whereas *Bacteroidota* were ubiquitous in all samples (Additional file 2: Fig. S6A). *Flavobacteriaceae* accounted for similar percentages in all fractions before May 4 (FL, 7–25%; PA3, 4–19%; PA10, 5–24%, Fig. 3). Afterwards, *Flavobacteriaceae* proportions increased rapidly during May 4 to May 8 and May 15 to May 29 in the FL (up to 30%) but not in both PA fractions (up to 18%) (Fig. 3). *Cryomorphaceae* relative abundances were higher in FL than in PA communities, while it was the opposite for *Saprospiraceae* (Fig. 3).

As reported for FL bacterioplankton sampled at Helgoland Roads in previous years [41, 42], SAR11 clade Ia, *Planktomarina*, and *Amylibacter* accounted for a substantial fraction of ASVs. These three alphaproteobacterial clades had high relative abundances in all FL samples but exhibited lower relative abundances in PA3 and were even rare in PA10 samples (Additional file 2: Fig. S7A). Members of alphaproteobacterial unclassified *Stappiaceae* were thriving in PA3 during the late *Phaeocystis* bloom, while alphaproteobacterial *Sulfitobacter* simultaneously ramped up in PA10 samples.

During the late diatom and *Phaeocystis* bloom phases, the FL bacterial community consisted primarily of *Bacteroidota*, including *Cd.* Prosiliicoccus [45], *Aurantivirga*, “Formosa”, and members of the NS3a and NS5 marine groups, *Gammaproteobacteria* including SAR92 and unclassified *Nitrincolaceae*, as well as a distinct group of *Verrucomicrobiota* including *Lentimonas* (Additional file 2: Fig. S7A-B). Members of *Cd.* Prosiliicoccus, the NS5 marine group, and *Aurantivirga* were also detected in the PA fractions but with lower relative abundances, some of which were probably due to carryover during fractionating filtration (Additional file 2: Fig. S7A). “Formosa” was present with similar low overall maximum relative abundance in FL and PA fractions (Additional file 2: Fig. S7B). *Algibacter* was mostly detected in the PA fractions and increased during the main bloom phases in PA3 communities (Additional file 2: Fig. S7B).

*Polaribacter* was not as abundant in FL communities as in previous [42] or later [39] years. More *Polaribacter* and unclassified *Saprospiraceae* were detected in both PA than in FL communities. *Maribacter* and *Winogradskyella* [21] were thriving during the late bloom phases but only in PA10 communities (Additional file 2: Fig. S7B).

During the bloom, *Gammaproteobacteria* had higher relative abundances in PA than in FL communities*.* For instance, in comparison with FL communities, members of the BD1-7 clade and *Colwellia* had higher relative abundances in PA communities during the diatom and *Phaeocystis* bloom phases, while unclassified *Nitrincolaceae* had higher relative abundances in PA3 communities (Additional file 2: Fig. S7A). Likewise, unclassified *Methylophagaceae* and *Glaciecola* exhibited higher relative abundances in PA10 communities (Additional file 2: Fig. S7A-B).

Furthermore, *Persicirhabdus* (*Verrucomicrobiota*) were proportionally more abundant in PA communities during the diatom bloom, and members of CL500-3 (*Planctomycetota*), known to be also associated with blooming freshwater algae [46], were proportionally more abundant in PA3 communities during the late diatom and *Phaeocystis* bloom phases. Similar bloom-associated temporal dynamics were also discernible in several groups that were not among the selected topmost genera. For instance, *Arenicella* and “Formosa” members were present only during the late diatom and *Phaeocystis* bloom phases in all fractions (Additional file 2: Fig. S7B). Likewise, members of the SUP05 cluster and NS7 marine group were present during the pre-bloom and early diatom bloom phases in FL communities, while at the same time, members of the DEV007 clade (*Verrucomicrobiota*) were present in PA3 communities (Additional file 2: Fig. S7B). Further composition dynamics at the ASV level are provided in Additional file 3.

### FL and PA community members exhibited distinct CAZyme composition dynamics

We selected 42 samples for metagenome sequencing, namely 18 FL (Illumina), 16 PA3 (Illumina, 8; PacBio, 8), and 8 PA10 (Illumina) samples (Additional file 1: Table S6). The resulting metagenomes amounted to 1.6 Tbp raw sequences. *K*-mer-based metagenome composition analyses corroborated significant differences between fractions (PERMANOVA, *p* = 0.003; Additional file 2: Fig. S8A). Distances between the pre-bloom and the two bloom periods were closer for FL than for PA data, corroborating 16S rRNA gene amplicon-based NMDS analyses (Fig. 2C). Individual assemblies of all metagenomes yielded 18.2 Gbp with 2.5 kbp minimum length (Additional file 1: Table S6).

**Fig. 4:**
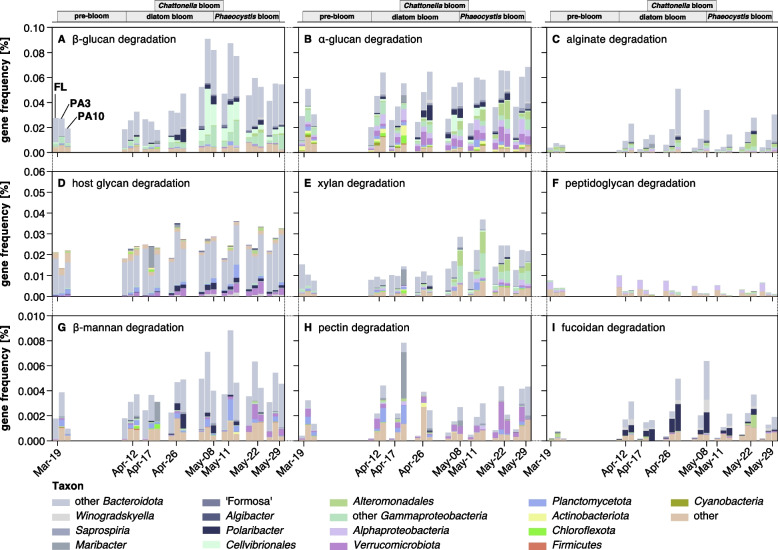
Frequencies of CAZyme genes attributed to the degradation of specific polysaccharide substrates. CAZyme genes in metagenomes and corresponding substrates were predicted using the dbCAN3-sub database. Gene frequencies were calculated as follows: frequency = Σ(average coverage of target genes) × 100/Σ(average coverage of all genes). Only dates with data for all fractions were plotted as stacked bar charts (left to right: FL, 0.2–3 µm; PA3, 3–10 µm; PA10, > 10 µm). Colors represent dominating taxa. Additional data for α-rhamnosides, chitin, arabinans, α-mannans, cellulose, and sialic acids is depicted in Additional file 2: Fig. S10, and complete data is summarized in Additional file 1: Table S7

We computed and compared CAZyme relative frequencies in assembled Illumina metagenome data over time (eight samples of each fraction, Additional file 1: Table S7). Overall, genes targeting β-1,3-glucan (laminarin) were most frequent, with peaking relative frequencies towards the end of the diatom bloom (Fig. 4A). Respective genes were dominated by *Bacteroidota* and *Gammaproteobacteria* in all fractions, whereas *Verrucomicrobiota* (more prominent in FL fractions) and *Planctomycetota* (more prominent in PA fractions) contributed only little (Additional file 1: Table S7). These data suggested an overall increase of laminarin-consuming bacteria when the diatom bloom collapsed, most notably in PA3 communities. This corroborates recent data from FL bacteria during the 2020 Helgoland spring bloom, where laminarin PULs were the most frequent and highest expressed of all PULs [39]. In terms of gene compositions, β-glucan PULs comprised the previously described variant-1 [39] coding for GH149, GH17, GH16, GH158 and GH30 enzymes (including variations), variant-2 coding for GH16 or GH17 and GH3 enzymes [39], and a PUL type coding only for GH16 enzymes (Additional file 2: Fig. S9).

Genes targeting α-glucans exhibited the second highest relative frequencies and exhibited no discernible trend (Fig. 4B). For the most part, respective genes were more frequent in PA than in FL communities. More *Gammaproteobacteria* and *Planctomycetota* contained these genes in PA than in FL communities. Four types of α-glucan PULs were present: type I coding for only one or more GH13 enzymes; type II coding for GH13, GH65, and sometimes an additional GH31 enzyme; type III coding for GH13, GH77, and GH57 enzymes; and type IV coding only for GH13 and GH31 enzymes (Additional file 2: Fig. S9). Genes targeting alginate were also frequent, with notable higher proportions in PA communities, particularly in PA10 (Fig. 4C).

Relative frequencies of host glycan degradation genes, e.g., genes targeting eukaryotic N-glycans, showed no trend in FL communities but were for the most part higher in PA communities, where they increased during the diatom and *Phaeocystis* bloom phases (Fig. 4D). Respective genes attributed to unclassified *Bacteroidota*, *Polaribacter*, *Verrucomicrobiota*, and *Planctomycetota*, with the latter preferring PA10 fractions*.*

Xylan degradation genes were rarer. Their relative frequencies ramped up in PA communities after the diatom bloom abated, in particular, in PA3 with high proportions of *Alteromonadales* and other *Gammaproteobacteria* during the *Phaeocystis* bloom (Fig. 4E). Genes targeting peptidoglycan (murein) were notably more frequent among FL than PA bacteria. Proportions were highest during the pre- and diatom bloom stages, and lower during the late bloom (Fig. 4F).

For α-mannans, there were no consistent differences between FL and PA communities (Additional file 2: Fig. S10D). Frequencies were highest during the early diatom bloom phase and leveled off towards the end of the *Phaeocystis* bloom. In contrast, proportions of genes for β-mannan degradation were often highest among PA3 bacteria, in particular, during the diatom to *Phaeocystis* bloom transition phase (Fig. 4G). A transition in α-mannan degradation from *Flavobacteriales* to other *Bacteroidota* was observed before, during, and after the bloom (Additional file 2: Fig. S10D), whereas bacterial communities harboring β-mannan degradation genes were dominated by *Planctomycetota*, *Verrucomicrobiota*, and *Bacteroidota* during the main and late bloom stages (Fig. 4G).

Proportions of genes targeting pectins (Fig. 4H) were notably higher in both PA fractions and increased during the diatom and late *Phaeocystis* blooms. During the diatom and *Phaeocystis* bloom phases, fucoidan degradation genes had much higher proportions in PA10 than in PA3 or FL communities, which coincided with a notable increase in the proportions of *Polaribacter*, *Maribacter*, and other *Bacteroidota* (Fig. 4I).

Genes for the degradation of sialic acids were more frequent in PA3 communities but ramped up in FL communities after the diatom bloom abated (Additional file 2: Fig. S10F). However, during the *Phaeocystis* bloom, sialic acid degradation potential appeared to have shifted towards PA communities. This shift was characterized by an increase in *Planctomycetota* and unclassified *Bacteroidota*, along with the emergence of *Saprospiria* (Additional file 2: Fig. S10F). Genes for the degradation of α-rhamnosides, chitooligosaccharides, arabinans, and cellulose were notably more frequent in both PA fractions and increased as the bloom progressed (Additional file 2: Fig. S10A-C, E).

### Representative PA and FL community MAGs

**Fig. 5:**
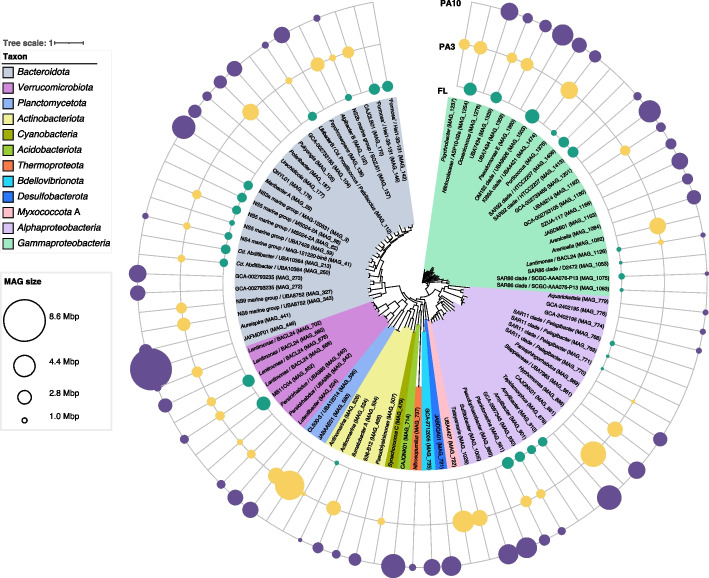
Sizes of the ten topmost abundant MAGs across each sample (*n* = 136). De-replication was carried out within each fraction, and MAGs belonging to the top ten in terms of abundance (as determined by MAG abundance) were specifically chosen for this analysis. The central maximum-likelihood tree of MAGs was computed in anvi’o v7.1 based on protein sequences of 38 universal single-copy genes. Circle area sizes correspond to MAG sizes with colors representing the fractions from which the MAGs were retrieved

From all 42 assembled metagenomes, we reconstructed 1944 initial bins (Illumina, 1721; PacBio, 223). Manual refinement retained 146 MAGs that fulfilled the MIMAG high-quality (HQ) criteria (> 90% completeness; < 5% contamination; presence of 23S, 16S, and 5S rRNA genes; and ≥ 18 tRNAs) [47], 964 MAGs (Illumina 895; PacBio, 69) of at least medium quality (MQ) (≥ 50% completeness, < 10% contamination), and 399 Illumina MAGs that did adhere to the “near complete” category by Almeida et al. [48] and were thus treated like HQ MAGs in downstream analyses (Additional file 1: Table S8, Additional file 2: Fig. S11). Dereplication of these in total 1509 MAGs at 95% ANI yielded 305 species-level MAGs of 16 known phyla (Additional file 1: Table S8, Additional file 2: Fig. S12), including 139 (45.7%) HQ MAGs. The average number of recovered MAGs per sample increased with decreasing filter pore size in fractionating filtration (PA10, 20; PA3, 26; FL, 57), due to the decreased capture of eukaryotic biomass [49]. The average size of HQ MAGs was larger in PA than in FL communities (Additional file 2: Figs. S13-14). Sizes of abundant MAGs ranged from 0.8 to 8.6 Mbp in PA and from 0.6 to 2.9 Mbp in FL communities (Fig. 5). Details are provided in Additional file 1: Table S9 and Additional file 3.

Based on 16S rRNA gene amplicon data (Additional file 1: Table S5), we selected 40 high-abundance genera (Additional file 1: Table S10), 39 of which were represented by corresponding MAGs. MAGs from FL community samples included previously identified relevant clades at Helgoland Roads, such as *Aurantivirga* [44], *Polaribacter* [50], “Formosa” species Hel1_33_131 [51], *Cd.* Prosiliicoccus [45], the NS4 and NS5 marine groups [52], *Amylibacter*, and the SAR11 Ia, SAR92, and SAR86 clades. MAG abundances based on read frequencies were also not unusual compared to earlier years with one notable exception. We detected *Polaribacter* clade 2-b [50] (Additional file 2: Fig. S15), but in agreement with ASV data, its maximum relative abundance of < 0.1% was much lower than in previous observations, with recorded maxima of 14.7% (2010), 19.8% (2011), and 34.5% (2012) [53].

More *Planctomycetota* (FL, 2; PA3, 9; PA10, 2) and *Chitinophagales* (FL, 2; PA3, 7; PA10, 5) MAGs were obtained from PA3 and PA10 metagenomes, whereas more alphaproteobacterial MAGs were obtained from FL metagenomes (FL, 31; PA3, 22; PA10, 23). Also, the genus diversity of *Flavobacteriaceae* MAGs was higher in PA than in FL communities (FL, 12; PA3, 17; PA10, 14). Based on ASV and MAG abundance data, we categorized MAGs into those that were most abundant in either FL or PA communities and those that were abundant in both. In total, 152 MAGs belonged to the abundant PA MAGs (Additional file 1: Table S11), including *Polaribacter*. Further details are provided in Additional file 3.

### Novel PA community MAGs

Comparison of all MAGs to those obtained for FL bacteria during the 2010 to 2016 spring blooms [43, 44] identified 100 species-level PA MAGs (HQ, 42; MQ, 58) that were uniquely obtained in 2018 (Additional file 1: Table S8, Additional file 2: Fig. S16). According to GTDB r207_v2, 76 of these MAGs represented novel species (Additional file 1: Table S8).

The 100 PA MAGs comprised a high proportion of over 3.5 Mbp (38/100 as compared to 57/305 for all species-level MAGs) (Additional file 2: Fig. S16C). They were dominated by *Paraglaciecola* (MAG_1218), *Pseudoalteromonas* (MAG_1211 and 1212), and UBA12014 (CL500-3, *Planctomycetota*, MAG_591) in PA3 and GCA-002793235 (*Vicingaceae*, MAG_272), GCA-002733465 (*Kangiellaceae*, MAG_1201), *Maribacter* A (MAG_26), *Polaribacter* (MAG_189), and *Pseudolysinimonas* (MAG_507) in PA10 communities (Additional file 2: Fig. S12, Additional file 3). We also obtained HQ MAGs of *Acidobacteriota* and *Chloroflexota*, plus seven of ten *Planctomycetota* MAGs and four *Polaribacter* MAGs representing species that we did not identify at Helgoland Roads before.

### Few particularly active but distinct MAGs dominated FL and PA communities

Seven sampled FL metaproteomes yielded 43,750 unique proteins (Additional file 1: Table S12). A total of 15,906 of these proteins were assigned to 177 FL MAGs, with up to 16.3% SusC- and SusD-like proteins and various TBDTs (Additional file 2: Fig. S17, Additional file 3). Such high proportions agree with previous metaproteome studies on bloom-associated bacterial communities [41, 43, 54]. Conversely, only 5018 proteins were obtained from three PA3 and PA10 metaproteome sampling dates, which were dominated by eukaryotic proteins (42.7 to 64.0%). Just 932 of these proteins could be assigned to bacterial MAGs (Additional file 1: Table S12, Additional file 3), which is why we used these data only to pinpoint the most active PA MAGs (Additional file 2: Fig. S18).

**Fig. 6:**
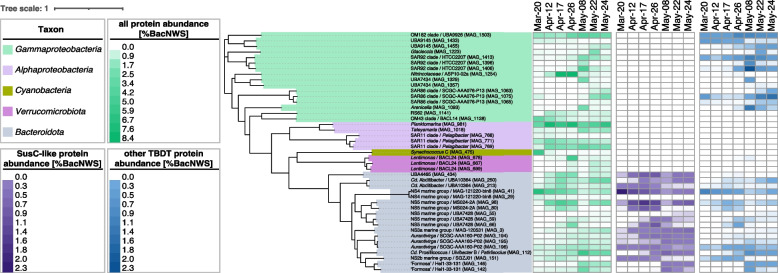
Topmost expressed bacterial MAGs obtained from sampled FL bacteria. In total, 44 highly expressed bacterial FL MAGs were selected, and a maximum-likelihood tree was computed in anvi’o v7.1 based on protein sequences of 38 universal single-copy genes. Taxonomic affiliations according to GTDB r207_v2 are provided for each MAG with corresponding Silva r138.1 affiliations in parenthesis. Corresponding phyla (class for *Proteobacteria*) are represented by background colors. Protein expression based on summarized protein abundances as a percentage of bacterial normalized weighted spectra (%BacNWS) are represented by heatmaps: (i) overall expression (shades of green), (ii) expression of SusC-like proteins (shades of purple), and (iii) expression of TonB-dependent transporters (TBDTs) except for SusC-like proteins (shades of blue)

Protein abundance data corresponded well with calculated MAG abundances from corresponding Illumina metagenomes (Additional file 2: Fig. S19). Abundant MAGs with high overall protein expression on all seven FL sampling dates comprised members of the NS4 marine group, *Planktomarina*, and the OM182 and SAR11 clade Ia clades. The highest overall expression was observed in a *Nitrincolaceae* ASP10-02a clade MAG, but only during the diatom bloom phase (Fig. 6). The expression of SusC-like proteins is indicative of oligosaccharide uptake in *Bacteroidota*. MAGs with high SusC-like protein expression during the diatom and *Phaeocystis* bloom phases comprised members of *Cd.* Prosiliicoccus, *Aurantivirga*, the NS3a, NS5, and NS4 marine groups, *Cd*. Abditibacter, and the *Cyclobacteriaceae* clade UBA4465 (Fig. 6). Other MAGs expressed SusC-like proteins only during distinct bloom phases, such as during the diatom bloom phase (other members of the NS5 marine group), the pre- and diatom bloom phases (a member of the NS2b marine group), the late diatom and *Phaeocystis* bloom phases (members of “Formosa”), or only the *Phaeocystis* bloom phase (again a member of the NS5 marine group).

Apart from bacteroidotal SusC-like proteins, TBDTs for the uptake of larger substrates, possibly including oligosaccharides, were predominantly expressed by members of various gammaproteobacterial clades. MAGs of the OM182, SAR92, and SAR86 clades exhibited high TBDT expression during all sampling dates, while others showed such expression mostly during the *Phaeocystis* bloom, e.g., MAGs of the SAR86 clade and *Glaciecola*.

In accordance with ASV data, *Polaribacter* were found to be only lowly abundant and hardly expressed in FL communities but prominent in PA communities (Additional file 2: Fig. S7A). Further data are shown in Additional file 2: Fig. S20. It is noteworthy that *Alphaproteobacteria* in FL communities expressed mostly ABC-type transporters, whereas in PA10 communities, members of the alphaproteobacterial genera *Parasphingopyxis*, *Parasphingorhabdus*, *Maricaulis*, and *Hyphomonas* featured the highest TBDT expressions (Additional file 2: Fig. S19). *Parasphingopyxis* species have been isolated from red macroalgae and *Maricaulis* from dinoflagellate phycospheres [55, 56], while *Parasphingorhabdus* species have been found in mollusk guts [57]. *Maricaulis* and *Hyphomonas* can attach to surfaces via prosthecae and feature complicated life cycles [58, 59]. Further details on active MAGs are provided in Additional file 3.

### Active CAZymes, PULs, and PUL-like clusters

Expressed CAZymes in FL community metaproteome data mapped to PULs and PUL-like clusters that were predicted to target host glycans, α-glucans, β-glucans, xyloglucans, fucose, alginate, and chitin (Additional file 1: Table S12). High expression was also observed for α-glucan degradation CAZymes with a peak on April 26, and β-glucan (laminarin) degradation CAZymes, which were particularly expressed during the diatom bloom’s end and the second bloom phase (May 8, 22, and 24) (Additional file 2: Fig. S21). On May 8, after the diatom bloom, also few CAZymes targeting fucose-containing polysaccharides were expressed. Complementary information on the PA metaproteome data is provided in Additional file 2: Fig. S18 and Additional file 3.

### MAG analyses highlight distinct polysaccharide degradation potentials in abundant FL and PA community members

Out of the 305 species-level MAGs of all fractions, 244 contained degradative CAZymes including 161 candidate PULs (*susCD* gene tandems plus at least 1 degradative CAZyme), 1056 PUL-like clusters (1 *susC*-, *susD*-like or other TBDT gene plus at least 1 degradative CAZyme), and 652 CAZyme-rich gene clusters (at least 3 degradative CAZyme genes) (Additional file 1: Table S13, Additional file 2: Fig. S22).

**Fig. 7:**
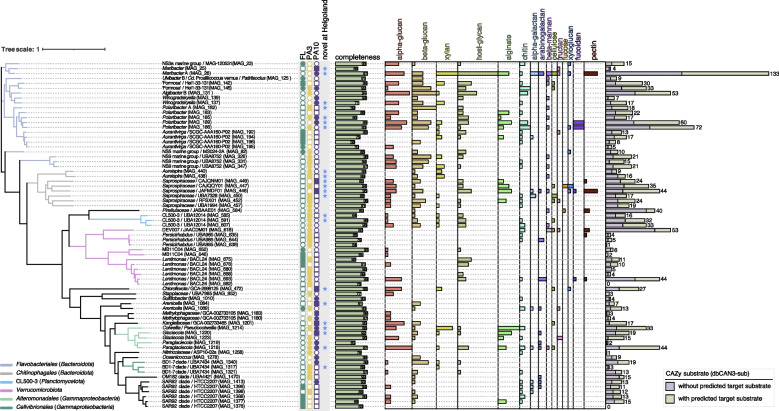
Predicted substrates of PULs and PUL-like clusters in abundant MAGs. Based on 16S rRNA amplicon and corresponding MAG abundance data, we selected 71 MAGs from bacterial clades that were prominent either during particular bloom phases and/or in particular size fractions. A maximum-likelihood tree (computed in anvi’o v7.1 based on protein sequences of 38 universal single-copy genes) is shown to the left. PULs and PUL-like clusters in these MAGs were annotated and corresponding target substrate classes were using the dbCAN3-sub database. The numbers of involved degradative CAZyme genes (GH, PL, CE) are represented by bar charts. The stacked bars to the very right correspond to the total number of CAZyme genes per MAG with and without predicted substrate specificities. In addition, the fractions are indicated where the MAGs were most abundant (FL, 0.2–3 µm; PA3, 3–10 µm; PA10, > 10 µm), with circles representing data obtained from corresponding ASVs and squares representing read-based MAG abundances. Further details are provided in Additional file 1: Table S7

We linked MAGs with 16S rRNA gene amplicon data to leverage the high temporal resolution amplicon data to uncover variations in MAG abundances (Additional file 1: Table S10, Additional file 2: Fig. S23), for which we selected the 71 most abundant MAGs for in-depth PUL analysis. Nine of these harbored 40 or more CAZyme genes, all of which were prevalent in PA communities (Fig. 7). A description of the most prominent MAGs, their links to ASVs and changes over time as well as their key CAZyme genes and inferred substrates is provided in Additional file 3, whereas a more holistic summary of the main results is provided in the subsequent discussion.

## Discussion

The 2018 spring phytoplankton bloom at Helgoland Roads was among the most diverse in terms of phytoplankton species richness that we analyzed since 2009 [42, 43], in particular, compared to that of 2020, where algal biomass was almost entirely dominated by few diatom species during two sharply separated bloom phases [39]. The 2018 spring bloom in contrast was characterized by more complex gradual successions of diatoms, raphidophytes, haptophytes, and—to a lesser extent—photosynthetic dinoflagellates.

In 2018, an influx of nitrate- and silicate-rich freshwater around April 10 was likely instrumental in bolstering the diatom bloom, which resulted in an almost complete consumption of free silicate within a fortnight. A second influx event around May 23 during the late *Phaeocystis* bloom coincided with the emergence of *Noctiluca scintillans*, a heterotrophic giant dinoflagellate (0.2–2 mm diameter) that frequently occurs in Helgoland waters from June to August [60]. *N. scintillans* was most probably transported with coastal waters to Helgoland, and since *N. scintillans* prey on *Phaeocystis* [61], likely contributed to the *Phaeocystis* bloom’s demise. Likewise, *Cryothecomonas* nanoflagellates, detected after the diatom bloom’s peak, prey on diatoms [62], and thus likely contributed to the termination of the diatom bloom.

The bacterioplankton responded to the spring bloom with swift successions of distinct clades, which were more dynamic in the more diverse PA communities. Some bacterial clades correlated with distinct phytoplankton bloom phases, e.g., *Polaribacter*, *Winogradskyella*, and unclassified *Nitrincolaceae* with the diatom bloom, and unclassified *Stappiaceae*, *Sulfitobacter*, and unclassified *Methylophagaceae* with the *Phaeocystis* bloom. Other clades were abundant during both, the late diatom and *Phaeocystis* bloom phases, e.g., *Cd*. Prosiliicoccus, “Formosa”, *Algibacter*, *Glaciecola*, and the BD1-7 clade.

The positive selection of bloom-adapted bacterial clades resulted in a decline in the diversity of FL bacteria and a size increase of the most abundant MAGs, notably in *Bacteroidota*, *Gammaproteobacteria*, and, to a lesser extent, *Planctomycetota*, *Verrucomicrobiota*, and *Alphaproteobacteria*. Diversity increased again during the collapse of the *Phaeocystis* bloom with the proliferation of more opportunistic generalists, such as members of the SAR86 clade and *Methylophagaceae* [63]. These clades featured smaller genomes, which was reflected in a decrease in the average size of abundant MAGs during the terminal bloom phase. Contrasting patterns were observed in both PA fractions, where diversities did not decrease during the main bloom phases, while the sizes of the most abundant MAGs decreased during the diatom bloom and increased notably during the late *Phaeocystis* bloom towards the bloom’s end. This illustrates that different selective forces shaped FL and PA communities.

### PA bacteria harbored more genes to degrade hardly soluble and structurally complex polysaccharides

Both abundant FL and PA bacteria featured high proportions of polysaccharide-degrading bacteria, however, with distinct CAZyme and PUL repertoires. Genes for the degradation of laminarins and α-glucans, both abundant, soluble, and structurally simply storage glucans, were the most prominent among FL bacteria, corroborating previous observations at Helgoland Roads [39]. Surprisingly, such genes were proportionally even more abundant among PA bacteria. Owing to sheer numbers, FL bacteria likely decomposed the bulk of laminarins and α-glucans, but the high proportion of respective genes in PA bacteria indicates that they are far from insignificant in this process. PUL analyses of 71 abundant MAGs from all fractions (Fig. 7) substantiated the salient role of storage glucans, as 40 contained β-glucan and 43 α-glucan PULs (Additional file 2: Fig. S9), fortifying the view that these glucans become available to PA bacteria that colonize senescent or dead algae.

Like during the 2020 spring bloom [39], α-glucan PULs were also dominated by type I α-glucan PULs in 2018. In addition to the previously described α-glucan PULs types I, II, and IV [39, 44], we also identified an additional type III comprising GH13, GH57, and GH77 (see [64]) genes in *Gammaproteobacteria*. MAG analyses showed an increase in PA *Gammaproteobacteria* with α- and β-glucan utilization genes after the diatom bloom (Additional file 2: Fig. S9). For example, MAG_1223 (*Glaciecola*) and MAG_1218 (*Paraglaciecola*) contained abundant genes for α-glucan hydrolysis, while MAG_1340 (BD1-7 clade) was rich in β-glucan hydrolytic genes (Fig. 7), suggesting that besides *Bacteroidota* also *Gammaproteobacteria* are significant α- and β-glucan consumers during algae die off phases.

Many marine macroalgae, e.g., *Saccharina* and *Fucus* brown algae, release gel-forming alginate and pectin-like polysaccharides [65]. Besides, alginate biosynthesis genes have been found in bloom-associated marine SAR92 clade *Gammaproteobacteria* [63]. Metatranscriptome analyses have furthermore suggested that alginate is an abundant bacterial substrate during spring blooms at Helgoland Roads [39]. Alginate and pectin degradation gene frequencies were notably more abundant in PA communities, reflecting the low solubilities of both substrates. Alginate gene frequencies in general prevailed over pectin degradation gene frequencies. This corroborates studies on the bacterial colonization of synthetic alginate and pectin particles by Bunse et al., where alginate was the preferred substrate [66]. We found alginate utilization genes predominantly in metagenome sequences attributed to unclassified *Bacteroidetes*, *Polaribacter*, and *Alteromonadales*. This was corroborated by MAG analyses, with alginate PULs present in PA *Saprospiraceae (Bacteroidota)*, *Polaribacter* (*Bacteroidota*), and *Colwellia* (*Alteromonadales*) (Fig. 7). These in situ data also support the in vitro experiments of Bunse et al., who identified *Colwellia* as among the primary colonizers on synthetic alginate particles [66].

Xylan degradation gene frequencies were largely stable among FL bacteria but increased considerably in PA bacteria during the late diatom and *Chattonella* bloom phases, surpassing FL frequencies more than twofold. This likely reflects an increased availability of structural xylans from disintegrating algae as well as poor xylan solubilities. Studies on the diatom *Thalassiosira weissflogii* have shown that its xylans and mannans are primarily found in POM and only little in DOM [67], consistent with functions as cell wall polysaccharides [30, 68]. We found xylan degradation genes in PA bacteria (e.g., *Colwellia*) and in FL bacteria (e.g., “Formosa”), but overall gene proportions suggest a higher proportion of xylan degrading bacteria among PA bacteria.

The bacterial cell wall polysaccharide peptidoglycan is also hardly soluble. However, peptidoglycan degradation gene [69] frequencies were substantially higher in FL than in PA bacteria, suggesting that peptidoglycan is rapidly solubilized and recycled. This is consistent with the fact that peptidoglycan is not known to significantly accumulate in POM [70].

Conversely, fucoidan utilization gene frequencies were consistently higher in PA than in FL communities. Apart from the well-known *Verrucomicrobiota* [71], we observed such genes also in *Polaribacter*-affiliating metagenome sequences during the diatom bloom. This was confirmed by the presence of fucoidan-targeting gene clusters in various PA MAGs, including *Polaribacter* MAG_186 and MAG_189, *Saprospiraceae* MAG_446 and MAG_449, *Planctomycetota* MAG_584, and *Lentimonas* MAG_693 (Fig. 7). *Polaribacter* MAG_189 was only abundant at the beginning of the diatom bloom, whereas *Polaribacter* MAG_186 prevailed during the diatom bloom, and the *Planctomycetota*, *Saprospiraceae*, and *Lentimonas* MAGs were most abundant during the *Phaeocystis* bloom. The presence of fucoidan throughout the bloom is plausible, since fucoidan-containing polysaccharides secreted by diatoms [67, 72] are rather persistent to bacterial degradation [73].

Genes for host glycan recognition, binding, and degradation were present in 33 of the 71 studied abundant MAGs, in particular in *Bacteroidota*, *Verrucomicrobiota*, and *Planctomycetota* (Fig. 7), and comprised GH92 (α-mannosidase) as well as GH20 and GH109 (β-1,6-N-acetylglucosaminidase) family genes. Host glycans are branched heteropolysaccharides that decorate eukaryotic host cell surfaces, e.g., mucin O-linked glycans, N-linked glycoproteins, and highly sulfated glycosaminoglycans (GAGs) in the human gut [74, 75]. Microalgae are also decorated with host glycans that are known to play a role in algal symbiont interactions with their hosts (e.g., [76]). In particular, mannose-rich N-glycans have been detected in microalgae [77, 78]. Binding to host glycans allows bacteria to initiate colonization of eukaryote surfaces, but host glycans also constitute an important substrate, not only for human gut bacteria but also for PA bacteria during phytoplankton blooms. For human gut *Bacteroides*, it has been shown that problematic antennary monosaccharides are removed from host glycans before uptake [75]. It is likely that such extracellular pre-digestion also occurs among marine bacteria, and if the selectively removed monosaccharides can diffuse away, this would explain the source of soluble sulfated methylpentoses (fucose, rhamnose) that constitute a preferential substrate for recurring small-celled FL *Verrucomicrobioata* at Helgoland Roads [79].

### The highly adaptable CAZome

CAZyme repertoires can vary considerably even between species of the same genus (e.g., [39]). For instance, *Winogradskyella* HQ MAG_139 harbored a much lower number of PULs and CAZyme-rich gene clusters than MAG_137, even though the latter was of lesser quality (94% vs 71%, Fig. 7). *Lentimonas* represents another illustrative case with three HQ MAGs, of which only MAG_693 contained abundant CAZyme genes (Fig. 7). CAZyme gene and PUL repertoires thus confer information about the adaptation of a given species towards a specific polysaccharide niche rather than its overall phylogenetic position in the tree of life. This implicates that the process of polysaccharide niche adaptation must considerably outpace the evolution of novel species, possibly by frequent lateral gene transfer [80].

CAZyme-rich MAGs often exhibited higher relative abundances during the bloom than closely related ones with fewer CAZymes, particularly in the PA fractions. This trend was evident for MAGs of *Maribacter*, *Winogradskyella*, *Polaribacter*, *Lentimonas*, and the CL500-3 and BD1-7 clades. *Lentimonas* MAG_693 for example harbored CAZyme genes targeting a variety of polysaccharide substrates (Fig. 7). Corresponding ASV data confirmed that this MAG represented a distinctively PA-associated species, consistent with a previous study on *Lentimonas* [81]. In this study, we found additional preferentially FL *Lentimonas* species, highlighting a broader niche spectrum within members of this genus (Fig. 7). Similarly, members of *Polaribacter* usually exhibit high FL abundances during diatom-dominated blooms at Helgoland Roads [50]. *Polaribacter* MAG_183 in this study corresponds to an abundant FL *Polaribacter* (MAG P_MB288) that we observed during the Helgoland spring bloom in 2020 [39]. However, in contrast to 2020, the dominating *Polaribacter* during the 2018 bloom were distinct and exhibited a clear preference for PA communities. In accordance with general trends, the dominant PA *Polaribacter* featured a larger MAG (MAG_189) with a higher number of CAZyme genes.

### Noteworthy clades with low CAZyme gene proportions

Members of the BD1-7 clade, unclassified *Stappiaceae*, and *Nitrincolaceae* featured high abundances but not CAZyme-rich MAGs. The gammaproteobacterial BD1-7 clade belongs to the group of Oligotrophic Marine *Gammaproteobacteria* (OMG) [82]. Members of this clade are known to associate with phytoplankton [83] as with other eukaryotes, such as sponges [84], corals [85], brown algae [86], and squids [87], where they may exert symbiotic functions. Alphaproteobacterial *Stappiaceae* are related to the abundant *Roseobacteraceae*. The genera include bacteriochlorophyll-producing *Roseibium*, members of which have been isolated from red algae [88], corals [89], oysters [90], and dinoflagellates [91], possibly also in a symbiotic function. *Nitrincolaceae* (formerly *Oceanospirillaceae*), abundant in PA3 communities, are opportunistic *Gammaproteobacteria* that frequently associate with phytoplankton (e.g., [92]), including *Reinekea* species, which we have observed in high abundance at Helgoland Roads before [42, 93]. Members of the *Nitrincolaceae* ASP10-2a clade are known to be diatom-associated [94].

### Bloom-associated PA bacteria and global carbon cycling

The bulk of particles during phytoplankton blooms are either formed directly by aggregation of algal necromass or indirectly via the formation and excretion of fecal pellets by grazing of small zooplankton. The latter have been estimated to consume almost two-thirds of the phytoplankton cells on a daily basis [13], which is why fecal pellets constitute high proportions of the POM during phytoplankton blooms. Copepods are abundant zooplankters and have short gut transmit times (30 to 90 min [95, 96]), which is why their fecal pellets contain considerable proportions of only partially degraded microalgae [97]. A substantial part of the captured PA communities in our study thus represent primary or secondary fecal pellet colonizers that consume residual pelleted algal polysaccharides. According to recent estimates, phytoplankton-specific loss rates to zooplankton grazing constitute the greatest uncertainty in CMIP6 marine biogeochemical models used to assess bacterial remineralization versus sequestration rates of algal biomass. These uncertainties range in the gigatons of carbon per year [98], which is substantial considering that recent anthropogenic carbon emissions have been estimated at around 10 gigatons per year (IPCC for the year 2018).

## Concluding remarks

Marine bacteria that colonize suspended particles inhabit a much more diverse habitat with ampler niche spaces and closer interactions than free-floating bacteria in the water column. However, to obtain quantitative data on bacterial polysaccharide degradation on particles in situ poses a considerable challenge. This applies in particular to reliable biochemical data on polysaccharide turnover rates, precise cell counts of PA bacteria, and even precise PA bacterial diversities owing to high proportions of chloroplast sequences in corresponding 16S rRNA gene amplicon data. Also, to obtain corresponding sufficiently deep metaproteome data remains a challenge. Finally, our sampling method does neither allow to discriminate different types of particles apart from broad size ranges nor to discriminate between loosely particle-associated and truly particle-attached bacteria—a limitation that we have discussed in detail in a previous study on the diversity, isolation, and cultivation of PA bacteria during the 2018 Helgoland spring bloom [21].

These challenges notwithstanding, we could demonstrate that PA bacterial communities were more diverse and underwent more dynamic changes in response to the 2018 spring phytoplankton bloom at Helgoland Roads than their FL counterparts. PA communities also featured a substantially higher metabolic potential for the degradation of a wide variety of polysaccharides. This was not only evident from assembled metagenome data but also in representative MAGs of abundant and active species. In the aforementioned study [21], we have also shown that PA bacteria represented less than 1% of the total bacterial community during spring 2018 at Helgoland Roads. However, considering that a major proportion of algal necromass passes through the POM pool, these bacteria must act as gatekeepers for the solubilization and subsequent remineralization of significant, yet-to-be-quantified proportions of algal polysaccharides during and after phytoplankton blooms, in spite of being considerably outnumbered by FL bacteria.

## Materials and methods

### Sampling, physicochemical, and phytoplankton data

Seawater samples were collected during spring 2018 (March 1 to May 29) off the North Sea island Helgoland (German Bight) at the LTER site “Kabeltonne” (54° 11.3′ N, 7° 54.0′ E, DEIMS.iD: https://deims.org/1e96ef9b-0915-4661-849f-b3a72f5aa9b1) by fractionating filtration (FL, 0.2–3 µm; PA3, 3–10 µm; PA10, > 10 µm) as described previously [42] (see Additional file 3 for details).

Wind direction data were obtained from the Climate Data Store of the Copernicus Climate Change Service [99]. Other physicochemical data, such as Secchi depth, water temperature, salinity, chlorophyll *a* content, dissolved inorganic nitrogen (NO_2_^−^, NO_3_^−^, NH_4_^+^), silicate, and phosphate as well as microscopic algae and zooplankton counts and taxonomic classifications were obtained as part of the Helgoland Roads LTER time series [100, 101]. Biovolumes of abundant plankters were determined in the framework of the Sylt Roads time series [102] as described elsewhere [103]. These data are summarized in Additional file 1: Table S1. Both the Helgoland and Sylt Roads time series are conducted by the Alfred Wegener Institute, Helmholtz Centre for Polar and Marine Research (Bremerhaven, Germany).

### 16S and 18S rRNA gene amplicon sequencing and analysis

Sequencing of 16S rRNA gene amplicons was performed at the Max Planck Genome Centre Cologne (Germany). DNA from biomass retained on filters was extracted as described elsewhere [21] and amplified using primers 341F and 805R targeting the V3 and V4 regions [104] for the FL samples, and primers 515F and 806R targeting the V4 region [105] for the PA3 and PA10 samples. Sequencing was carried out on an Illumina HiSeq 2500 (Illumina, San Diego, CA, USA) in rapid mode with 2 × 250 bp paired-end reads.

Sequences were analyzed for single nucleotide-resolved amplicon sequence variants (ASVs) using the DADA2 v1.19.2 package [106] with R v4.0.3 (http://www.R-project.org) (Additional file 3). ASVs assigned to chloroplasts, mitochondria, *Eukarya*, *Archaea*, or unclassified sequences were excluded from further analyses (Additional file 2: Fig. S6B). Possible impacts of the different primer sets and the omission of rarefaction are provided in Additional file 3, as well as details on the analysis of the 18S rRNA amplicon data.

### Metagenome sequencing and assembly

Metagenomes were sequenced at the Max Planck Genome Centre Cologne, 34 on an Illumina HiSeq 2500 using 2 × 150 bp chemistry, and eight additional PA3 metagenomes on a PacBio Sequel II (Menlo Park, CA, USA) using one SMRT cell per sample in long-read HiFi mode. The quality of Illumina reads was assessed with FastQC v0.11.9 [107].

Quality-filtered reads from FL metagenomes were assembled individually within SPAdes v3.11.1 [108]. Quality-filtered reads from PA3 and PA10 Illumina metagenomes were assembled individually using MEGAHIT v1.2.9 [109]. Assemblies of PA3 PacBio metagenomes were generated using Flye v2.9.1 [110] (Additional file 3). Assembly quality was assessed with QUAST [111]. Contigs below 2.5 kbp were removed using *anvi-script-reformat-fasta* within anvi’o v6.2 [112].

### Metagenome-assembled genome (MAG) retrieval and analysis

MAG retrieval was performed as described previously [43] (Additional file 3). MAGs were classified into low-, medium-, and high-quality categories according to the criteria described in Bowers et al. [47] using CheckM v1.1.3 [113]. Only medium- and high-quality MAGs were used in further analyses. Dereplication was done using dRep v3.0.0 [114] with an average nucleotide identity (ANI) > 95%. ANI was calculated using FastANI [115]. MAG abundances were calculated as described previously [116] (Additional file 3). 16S rRNA gene sequences were extracted from MAGs using barrnap v0.9 (https://github.com/tseemann/barrnap) and subsequently classified in the Silva Incremental Aligner (SINA) with Silva SSU 138.1 taxonomy [117]. MAG taxonomies were determined by GTDB-Tk v2.1.0 [118, 119] with GTDB release R207_v2. Differences in the denominations of taxa between Silva and GTDB were resolved as described previously [44]. A phylogenomic tree of dereplicated MAGs was constructed using FastTree [120] from within anvi’o v7.1 and visualized using interactive Tree of Life (iTol) v6.5.6 [121].

### Interrelation of 16S rRNA gene amplicon and MAG data

Blastn was used to search all prevalent ASVs from abundant genera within the 16S rRNA gene amplicon dataset against all MAG-derived 16S rRNA gene sequences. For identical hits with 100% coverage, we assumed that changes in ASV relative abundance reflected changes of the corresponding MAG over time. Since not all MAGs contained 16S rRNA genes, we extended our search to all MAGs that we obtained from the Helgoland metagenome samples from 2010 [44], 2012 [44], 2016 [43, 44], and 2020 [39]. MAGs from 2018 without 16S rRNA gene sequence were considered to match MAGs from other sampling years, if both exhibited an ANI of at least 95%. In addition, we included two matching MAGs from the GTDB database. Details are provided in Additional file 1: Table S10.

### Metagenome and MAG annotation

For assembled Illumina metagenomes, protein-coding sequences were predicted using Prodigal [122], Aragorn [123], and barrnap (https://github.com/tseemann/barrnap) as implemented in Prokka v1.14.6 [124] (default settings). For PacBio data, FragGeneScan v1.31 [125] was used (setting *-w 1*) due to a higher number of frameshifts. Functional MAG annotations were done as described in Additional file 3.

### Gene frequency analyses

Eukaryotic and unclassified reads were removed from unassembled Illumina metagenomes according to Kaiju v1.9.0 [126] annotations. Metagenomes were subsequently assembled with MEGAHIT v1.2.9, and frequencies of genes of interest were computed for each metagenome as follows: gene frequency = (sum of average coverage of target gene(s)) × 100 / (sum of average coverage of all genes) [42]. The average coverages of target genes were determined in SqueezeMeta v1.3.1 [127] using bowtie2 [128] for mapping. CAZymes were predicted as described in Additional file 3.

### Prediction of CAZyme-rich gene clusters and PULs

CAZyme-rich gene clusters and PULs were identified in a sliding window approach as described previously [40, 43] with a window length of ten genes. When at least three genes within the window coded for either GHs, PLs, CEs, sulfatase, TBDTs, or SusD-like proteins, we considered this a candidate locus. The resulting CAZyme-rich loci were manually annotated based on a combination of multiple databases (Additional file 3). Putative target substrate classes of PULs, PUL-like, and CAZyme-rich gene clusters in MAGs were predicted using the dbCAN3-sub database [129].

### Metaproteome analyses

Metaproteomes were analyzed on seven dates for FL samples (2018/03/20, 2018/04/12, 2018/04/17, 2018/04/26, 2018/05/08, 2018/05/22, 2018/05/24) and on three dates for PA samples (2018/04/17, 2018/05/08, 2018/05/24). Proteins were extracted from filtered biomass and subsequently analyzed as described elsewhere [43, 130, 131] (Additional file 3).

### Supplementary Information

**Additional file 1:** Supplementary tables.

**Additional file 2: **Supplementary figures.

**Additional file 3: **Supplementary text.

## Data Availability

Metagenome reads, assemblies, and MAGs were deposited in the European Nucleotide Archive (ENA) under project numbers PRJEB38290 and PRJEB67502. 16S rRNA gene amplicon sequences of FL and PA fractions were deposited in ENA under project numbers PRJEB51721 and PRJEB51816, respectively. Mass spectrometry proteome data were deposited at the ProteomeXchange Consortium via the PRIDE partner repository [132]. Original mass spectrometry proteome data of FL bacteria are accessible as project PXD042676 and data for PA bacteria as project PXD046705.
