# Supplementary material for: Particle-attached bacteria act as gatekeepers in the decomposition of complex phytoplankton polysaccharides
Source: Microbiome. 2024 Feb 20;12:32. doi: 10.1186/s40168-024-01757-5 (PMC10877868; doi:10.1186/s40168-024-01757-5)
Supplement: Supplementary file 3 — Additional file 2: Supplementary figures. [file 40168_2024_1757_MOESM2_ESM.pdf]

*Microbiome - Research*

# Particle-attached bacteria act as gatekeepers in the decomposition of complex phytoplankton polysaccharides

Feng-Qing Wang[1], Daniel Bartosik[2,3†], Chandni Sidhu[1†], Robin Siebers[4], De-Chen Lu[1,5], Anke Trautwein-Schult[4], Dörte Becher[4], Bruno Huettel[6], Johannes Rick[7], Inga V. Kirstein[7], Karen H. Wiltshire[7], Thomas Schweder[2,3], Bernhard M. Fuchs[1], Mia M. Bengtsson[4*], Hanno Teeling[1*], Rudolf I. Amann[1*]

[1] Max Planck Institute for Marine Microbiology, Celsiusstraße 1, 28359 Bremen, Germany

[2] Institute of Pharmacy, University of Greifswald, Felix-Hausdorff-Straße 3, 17489 Greifswald, Germany

[3] Institute of Marine Biotechnology, Walther-Rathenau-Straße 49a, 17489 Greifswald, Germany

[4] Institute of Microbiology, University of Greifswald, Felix-Hausdorff-Straße 8, 17489 Greifswald, Germany

[5] Marine College, Shandong University, Weihai 264209, China

[6] Max Planck Genome Centre Cologne, Carl von Linné-Weg 10, 50829 Köln, Germany

[7] Alfred Wegener Institute for Polar and Marine Research, Biologische Anstalt Helgoland, 27483 Helgoland, Germany

[†] These authors contributed equally to this study.

* Corresponding authors:
Rudolf I. Amann, Max Planck Institute for Marine Microbiology, Celsiusstraße 1, 28359 Bremen, e-mail: ramann@mpi-bremen.de, phone: +49 421 2028 9300

Hanno Teeling, Max Planck Institute for Marine Microbiology, Celsiusstraße 1, 28359 Bremen, e-mail: hteeling@mpi-bremen.de, phone: +49 421 2028 9760

Mia M. Bengtsson, University of Greifswald, Felix Hausdorff-Straße 8, 17489 Greifswald, Germany, e-mail: mia.bengtsson@uni-greifswald.de, phone: +49 383 4420 4212

E-mail addresses and telephone numbers of all authors:


| | | |
|---|---|---|
| Feng-Qing Wang | fwang@mpi-bremen.de | +49 421 2028 9390 |
| Daniel Bartosik | daniel.bartosik@uni-greifswald.de | +49 3834 420 4918 |
| Chandni Sidhu | csidhu@mpi-bremen.de | +49 421 2028 9582 |
| Robin Siebers | siebersr@vodafonemail.de | +49 3834 420 5931 |
| De-Chen Lu | 202267000015@sdu.edu.cn | +86 0631 5688303 |
| Anke Trautwein-Schult | anke.trautwein-schult@uni-greifswald.de | +49 3834 420 5922 |
| Dörte Becher | dbecher@uni-greifswald.de | +49 3834 420 5903 |
| Bruno Hüttel | huettel@mpipz.mpg.de | +49 221 5062 828 |
| Johannes Rick | johannes.rick@awi.de | +49 4651 956 4220 |
| Inga V. Kirstein | inga.kirstein@awi.de | +49 4725 819 3153 |
| Karen H. Wiltshire | Karen.Wiltshire@awi.de | +49 4651 956 4112 |
| Thomas Schweder | schweder@uni-greifswald.de | +49 3834 420 4212 |
| Bernhard M. Fuchs | bfuchs@mpi-bremen.de | +49 421 2028 9350 |
| Maria M. Bengtsson | mia.bengtsson@uni-greifswald.de | +49 421 2028 9390 |
| Hanno Teeling | hteeling@mpi-bremen.de | +49 421 2028 9760 |
| Rudolf I. Amann | ramann@mpi-bremen.de | +49 421 2028 9300 |


## Conflict of interest

The authors declare no conflict of interest.

**Supplementary Figures for this manuscript include the following:**

Supplementary Fig. S1 to S25.

## Supplementary Figures

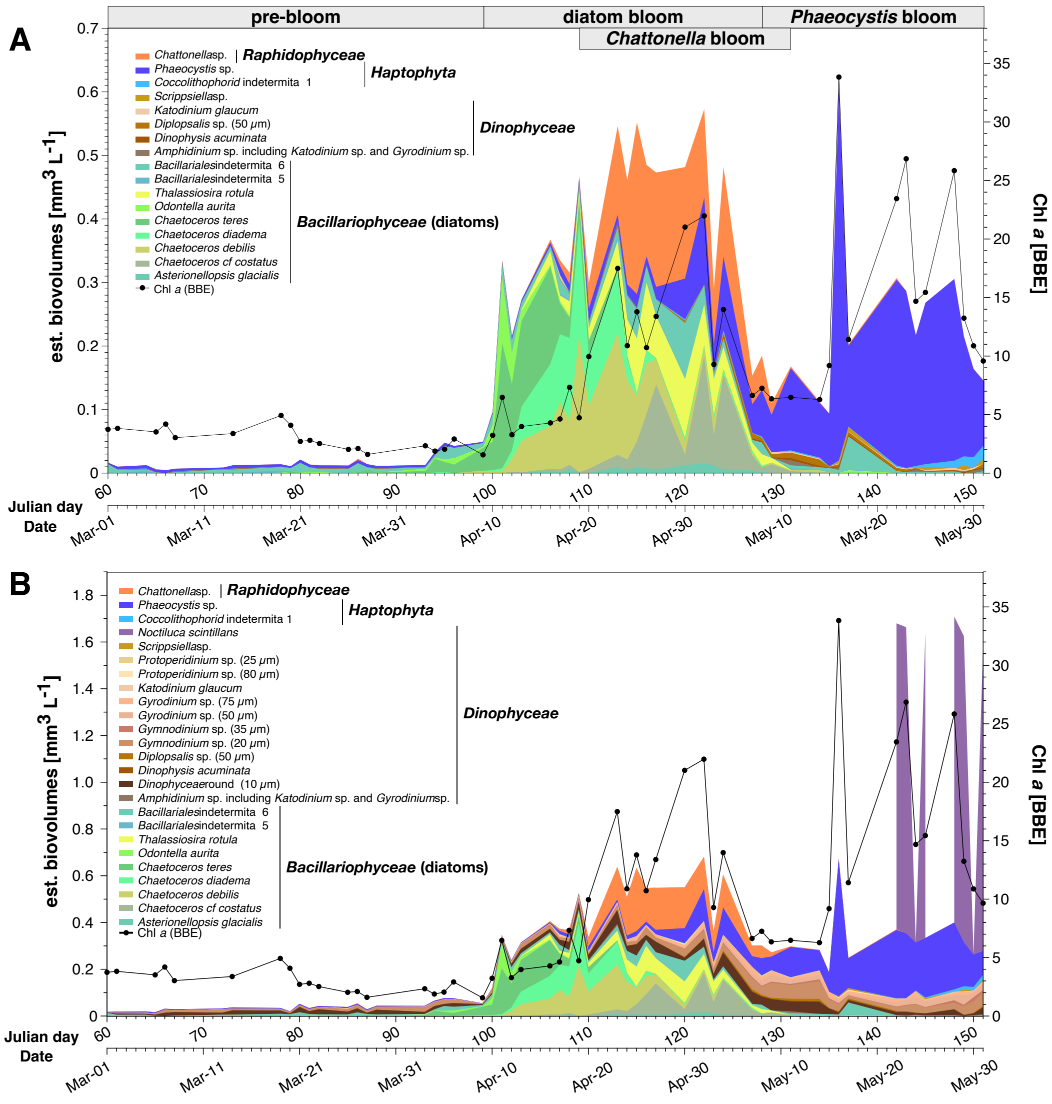


**Fig. S1. 2018 spring phytoplankton bloom at Helgoland Roads.** Estimated biovolumes of microscopically determined abundant plankton taxa (stacked colored areas) and chlorophyll *a* measurements (black line) as assessed by fluorescence-based algal group analyzer measurements (AlgaeLabAnalyser, BBE Moldaenke GmbH, Schwentinental, Germany). **A** photosynthetic plankters, **B** all plankters.

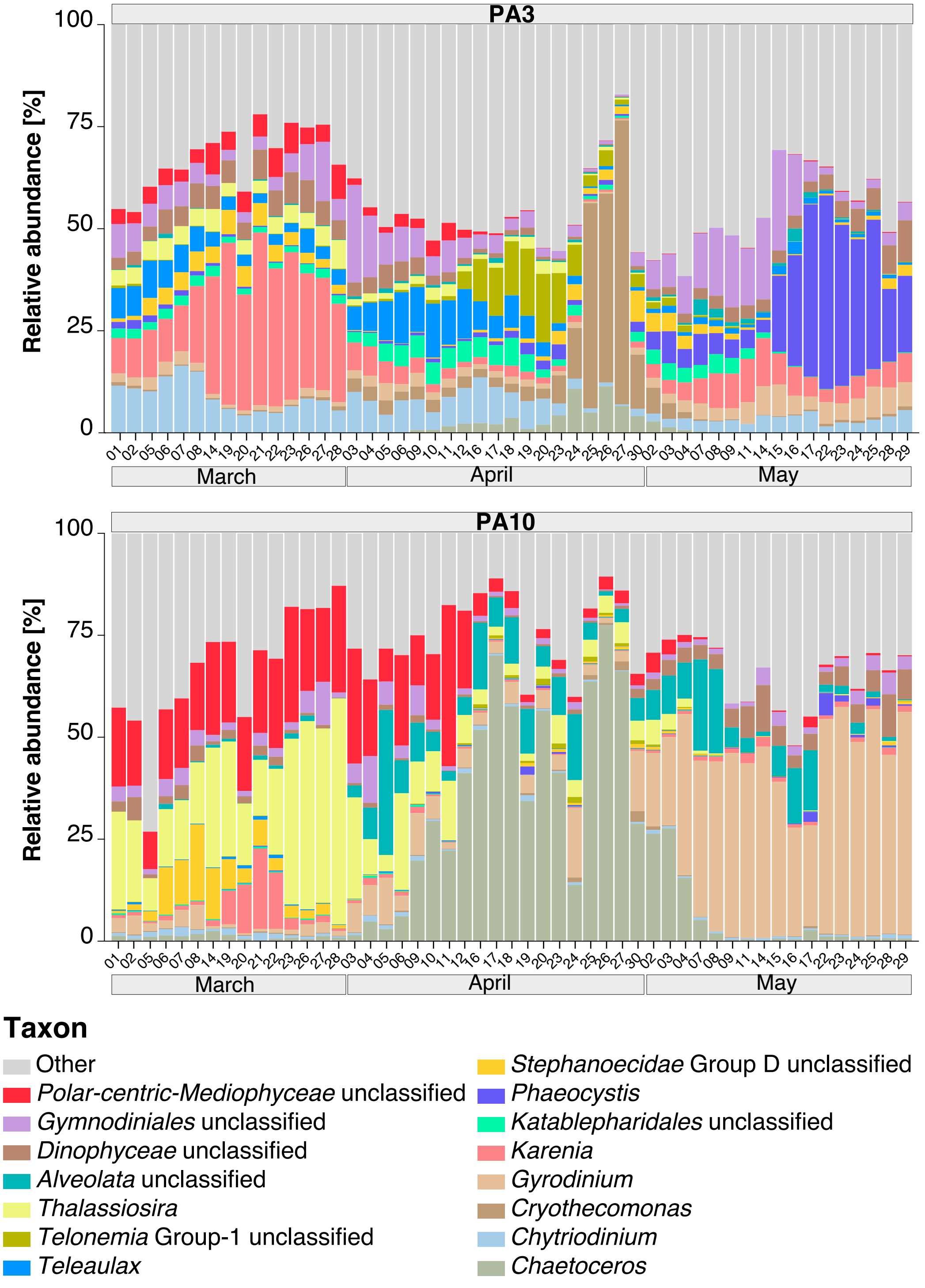


**Fig. S2. Abundant 18S rRNA gene ASVs in PA fractions.** Shown are the collective relative abundances of the 15 most abundant ASVs in each sample. PA3: 3-10 $\mu$m, PA10: >10 $\mu$m.

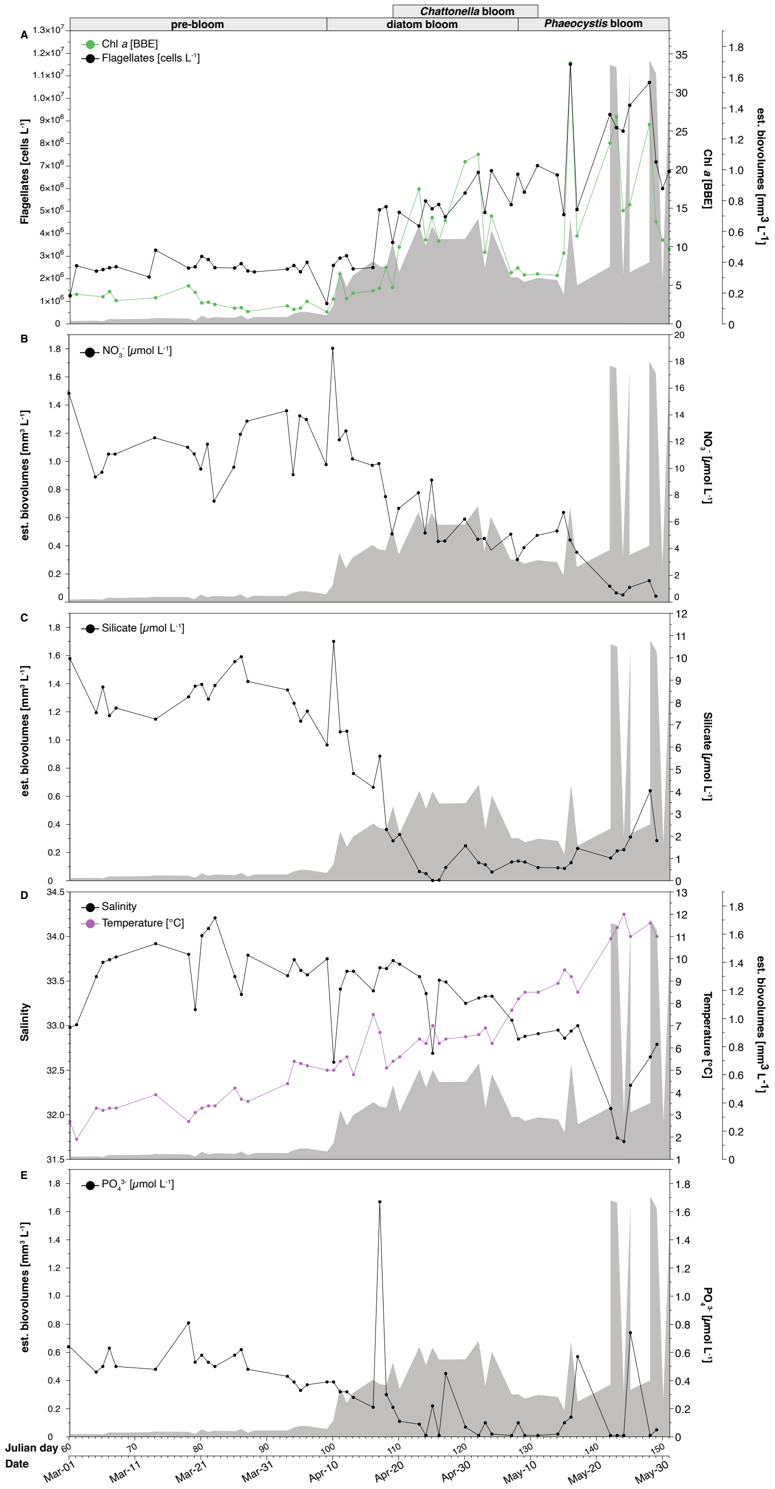


**Fig. S3. Flagellate cell counts and physicochemical data.** Data are represented by line graphs with biovolume estimates of abundant phytoplankton and non-photosynthetic dinoflagellates as background (see Fig. S1B). The distinct bloom stages are indicated on the top. Corresponding data is provided in Table S1 in Additional file 1.

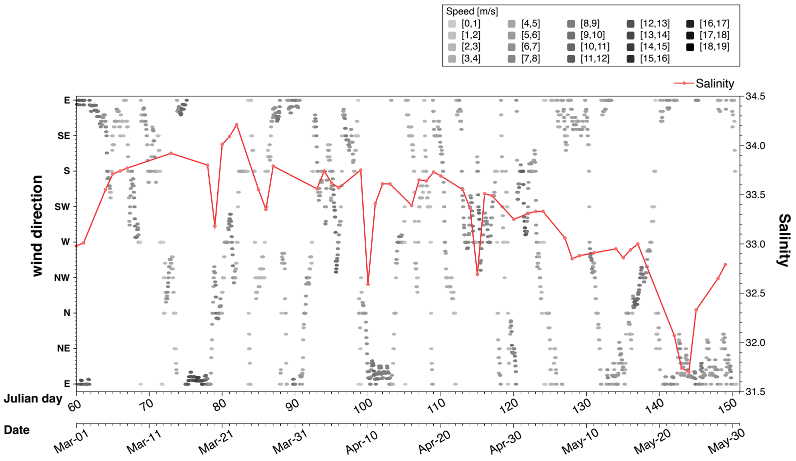


**Fig. S4. Wind directional and salinity data at Helgoland Roads during March to May 2018.** Wind components at 10 m above the sea surface were obtained from the Climate Data Store of the Copernicus Climate Change Service (ERA 5 product). Corresponding raw data is provided in Table S3 in Additional file 1. Additional weather data is provided in Table S4 in Additional file 1.

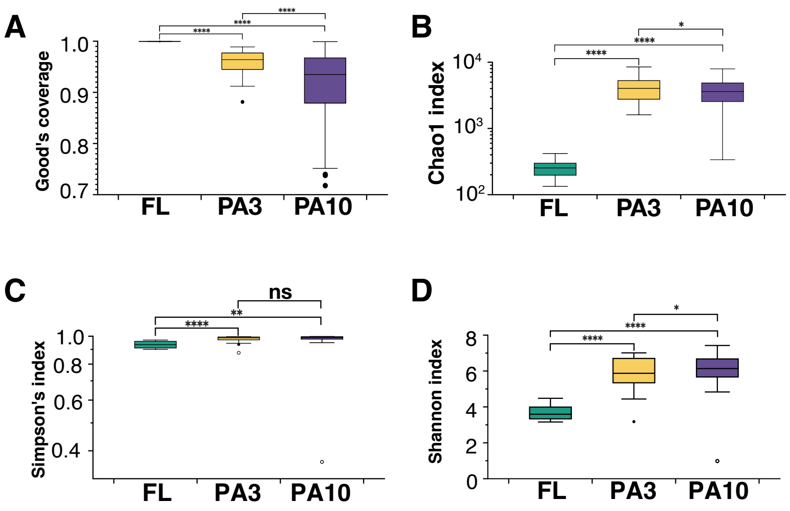


**Fig. S5. Sample diversity indices based on 16S rRNA gene ASVs.** Sample diversities of the bacterial communities of all size fractions (FL: 0.2-3 $\mu$m, PA3: 3-10 $\mu$m, PA10: >10 $\mu$m) were assessed by **A** Good's coverage, **B** Chao1, **C** Simpson and **D** Shannon indices. Statistical significance was assessed using one-way ANOVA (ns: not significant; *: $p < 0.05$; **: $p < 0.01$; ***: $p < 0.001$; ****: $p < 0.0001$).

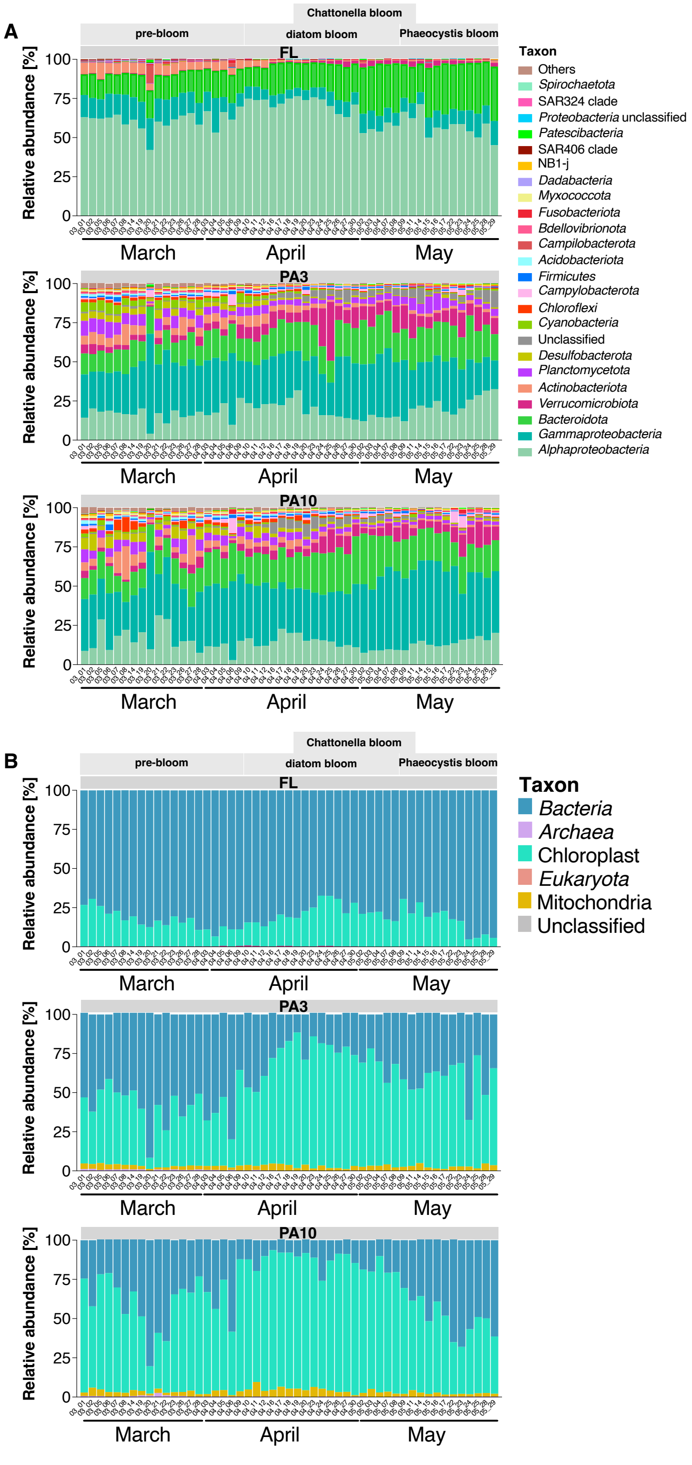


**Fig. S6. Community composition as assessed by 16S rRNA gene amplicon data at higher taxonomic levels. A** Phylum level taxa with *Proteobacteria* represented by *Alphaproteobacteria* and *Gammaproteobacteria* classes. **B** Domain level taxa. Shown are the collective relative abundances of the ten most abundant phyla in each sample. FL: 0.2-3 $\mu$m, PA3: 3-10 $\mu$m, PA10: >10 $\mu$m.

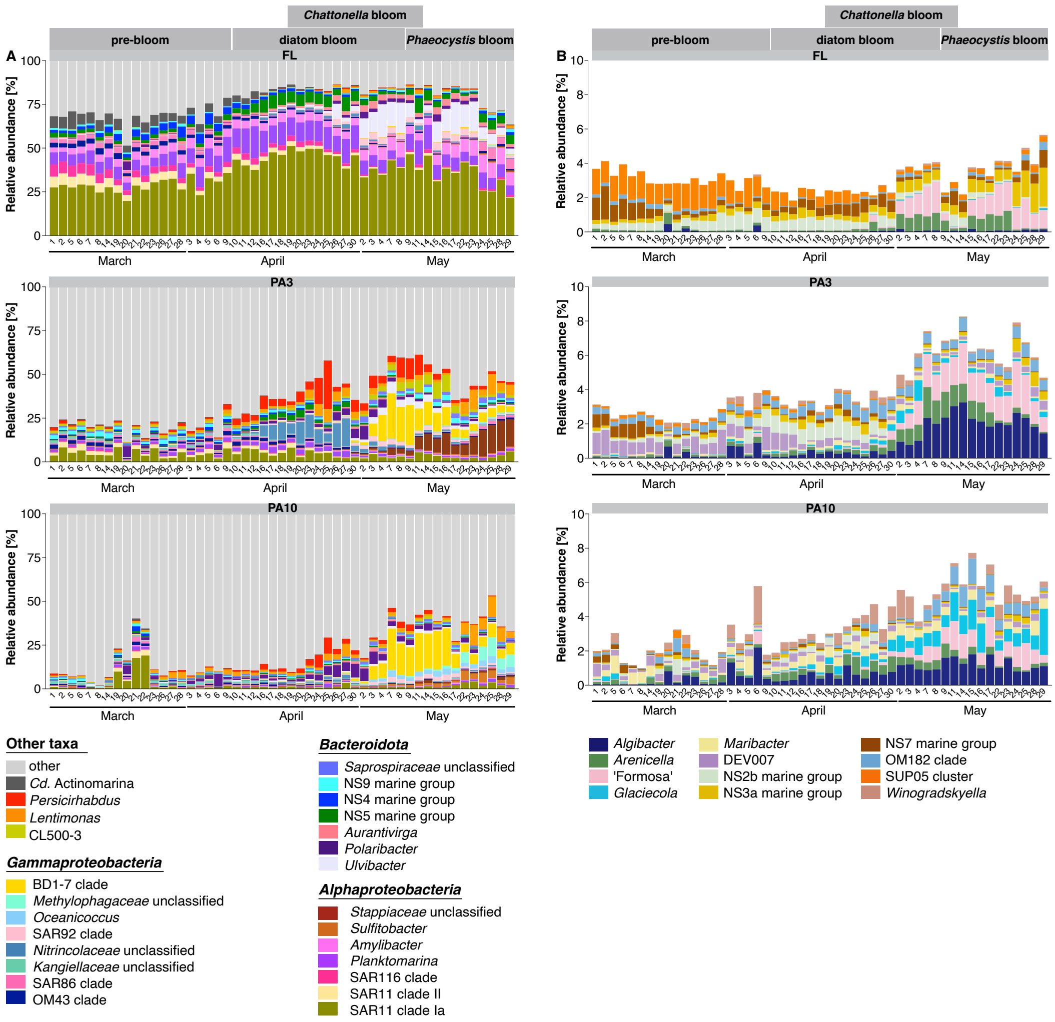


**Fig. S7. Compositional differences among fractions as assessed by 16S rRNA gene amplicon data. A** Collective relative abundances of the five most abundant genera in each sample for all three fractions (FL: 0.2-3 $\mu$m, PA3: 3-10 $\mu$m, PA10: >10 $\mu$m). Genera with high relative abundances in only few samples were subsumed as “other” in order to reduce complexity. Details are provided in Table S5 in Additional file 1. **B** Rarer genera with either clear preferences for distinct fractions or specific bloom phases.

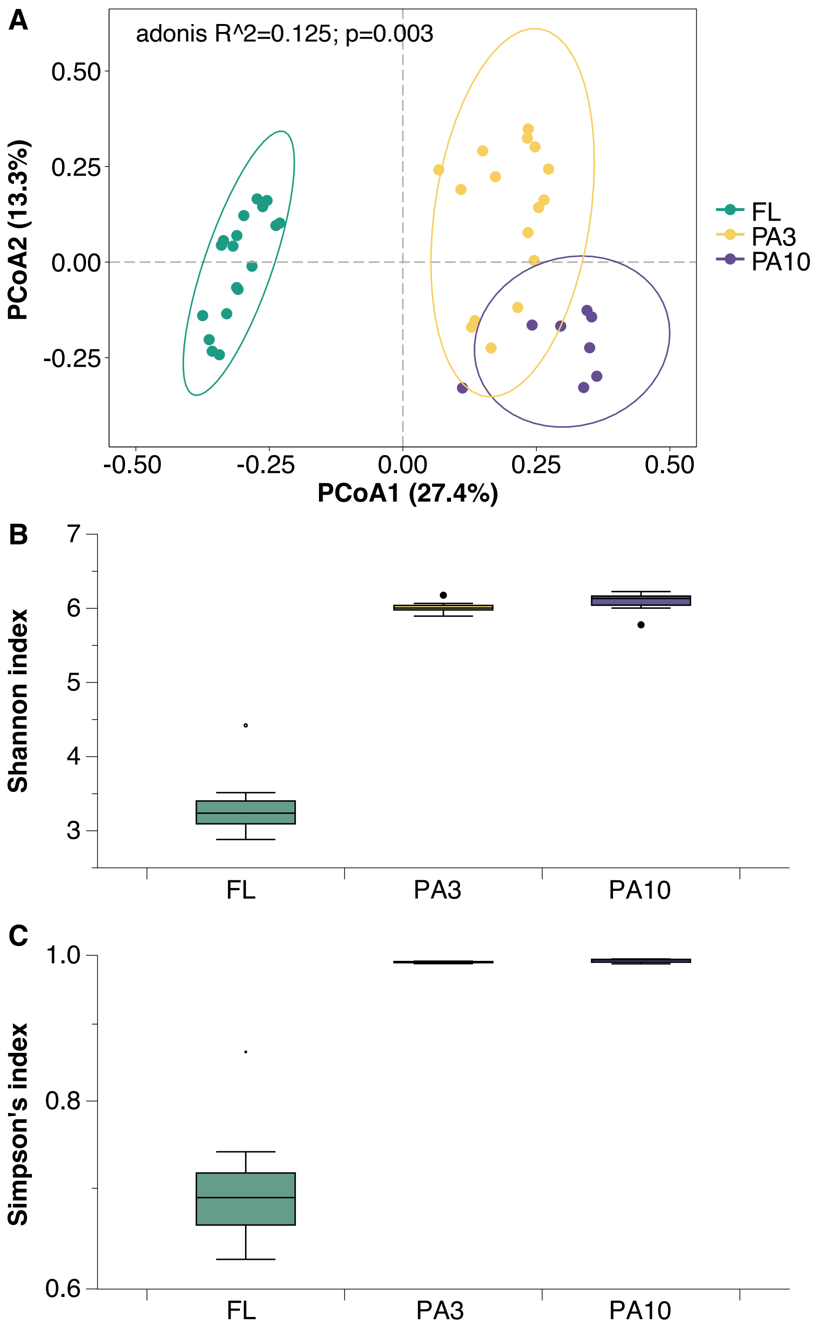


**Fig. S8. Diversity analysis of 2018 Helgoland spring bloom metagenomes.** **A** Principal component analysis of AB-Jaccard distances computed from k-mer read analysis in Simka v1.5.3 for 24 Illumina metagenomes (eight for each of the 0.2-3 $\mu$m FL, 3-10 $\mu$m PA3 and >10 $\mu$m PA10 size fractions). **B** Shannon and **C** Simpson's diversity indices of the sampled bacterial communities using the same 24 Illumina metagenomes. Corresponding data are provided in Table S14 in Additional file 1 and methodological details are provided in Additional file 3. Colors correspond to sample fractions.

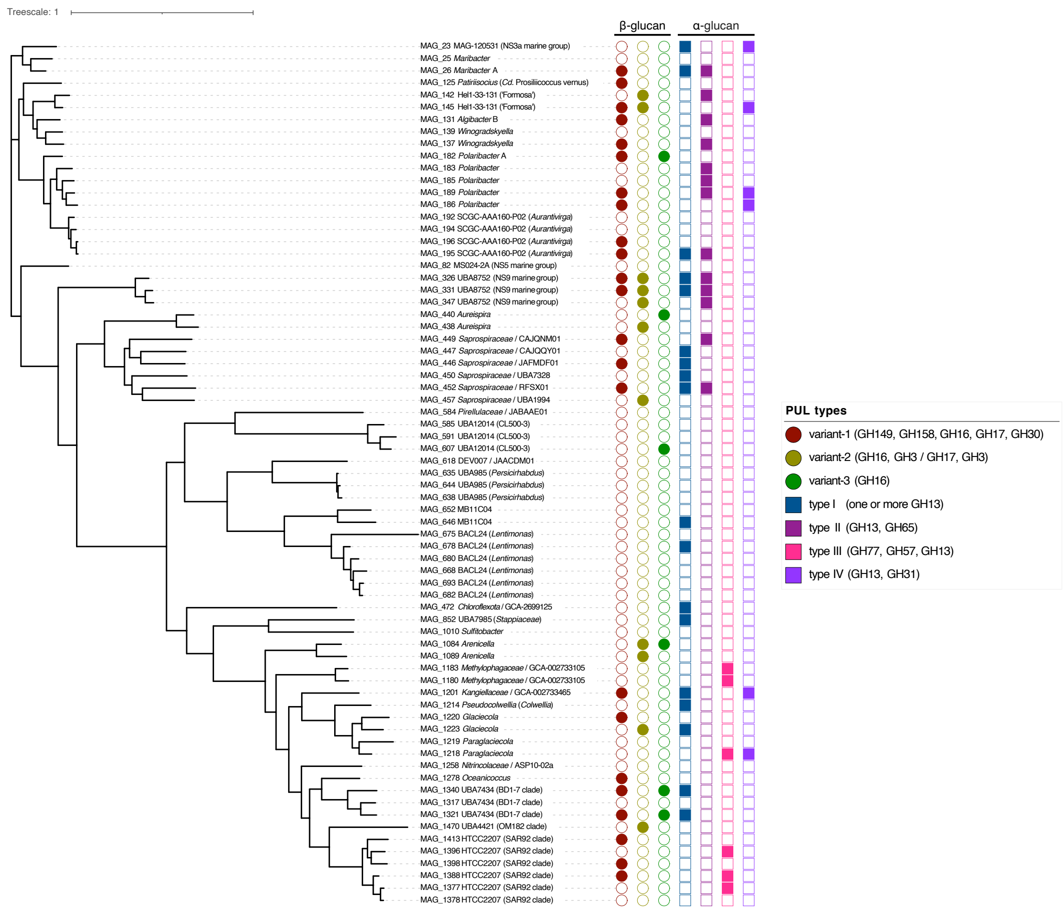


**Fig. S9. α- and β-glucan PUL-types & CAZyme clusters in 71 selected abundant MAGs.** β-glucan PULs - variant-1: GH149, GH158, GH16, GH17, GH30; variant-2: GH16, GH3/GH17, GH3; variant-3: GH16. α-glucan PULs - type I: one or more GH13; type II: GH13, GH65; type III: GH77, GH57, GH13; type IV: GH13, GH31.

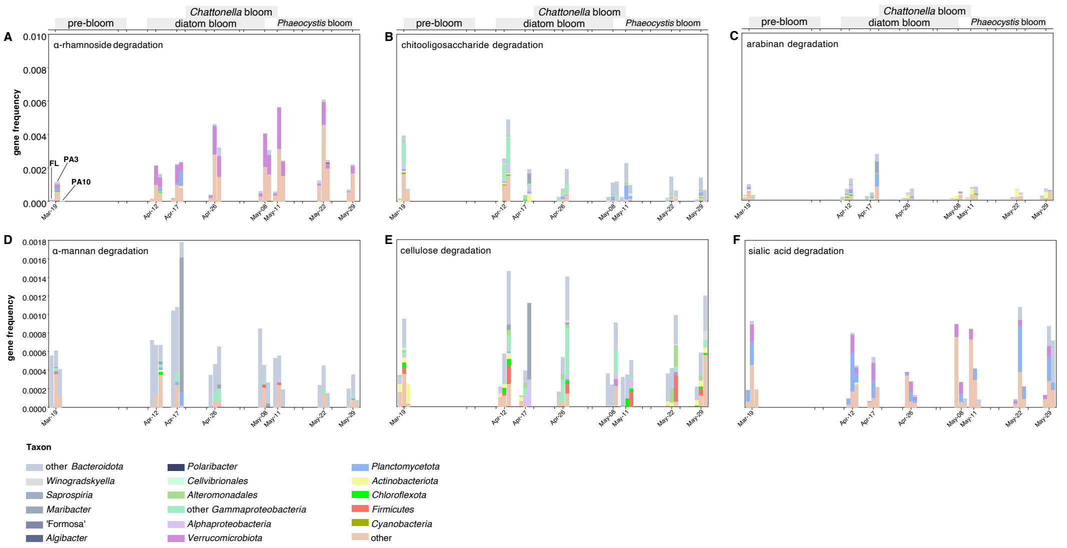


**Fig. S10. Frequencies of CAZyme genes attributed to the degradation of A α-rhamnosides, B chitooligosaccharides, C arabinan, D α-mannan, E cellulose and F sialic acids.** CAZymes for the degradation of predicted substrates were extracted from all metagenomes (Table S7 in Additional file 1), and gene frequencies were calculated as follows: frequency = Σ(average coverage of target genes) *100 / Σ(average coverage of all genes). Substrate predictions were annotated using the dbCAN3-sub database. Only dates with data for all fractions were plotted as stacked bar charts (left to right: FL: 0.2-3 $\mu$m, PA3: 3-10 $\mu$m, PA10: >10 $\mu$m). Dominating taxa are highlighted by colors.

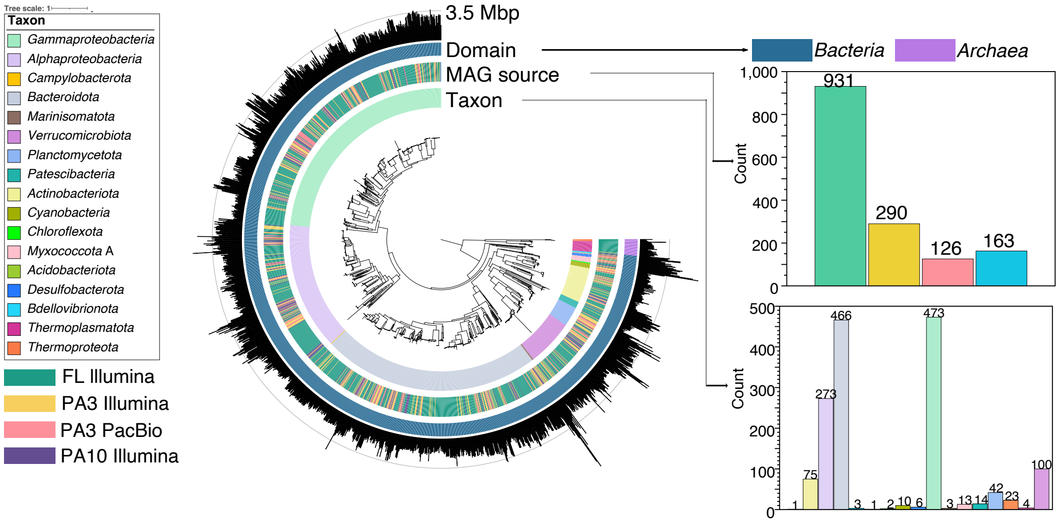


**Fig. S11. Overview of the MAGs reconstructed from the metagenome dataset of the 2018 Helgoland spring bloom.** Maximum-likelihood tree of 1,509 MAGs calculated in anvi'o v7.1 based on protein sequences of 38 universal single-copy genes with surrounding circles representing (inside to outside): (i) phylum-level taxonomy (class for *Proteobacteria*), (ii) size fraction of origin and sequencing platform (FL: 0.2-3 $\mu$m, PA3: 3-10 $\mu$m, PA10: >10 $\mu$m), (iii) domain-level taxonomy, (iv) circular bar chart representing MAG sizes (dotted line: 3.5 Mbp).

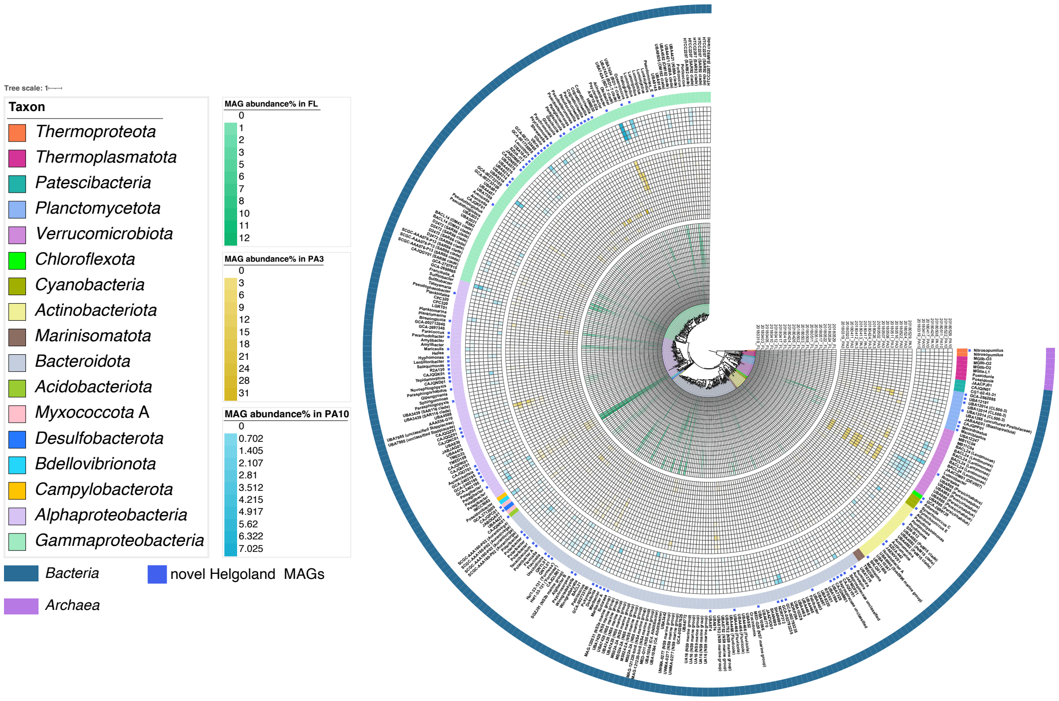


**Fig. S12. Overview of dereplicated MAGs and their calculated relative abundances during the 2018 Helgoland spring bloom.** Maximum-likelihood tree of 305 dereplicated MAGs calculated in anvi'o v7.1 based on protein sequences of 38 universal single-copy genes with surrounding circles representing (inside to outside): (i) FL MAG abundances (green), (ii) PA3 MAG abundance (yellow), (iii) PA10 MAG abundances (turquoise). (FL: 0.2-3 $\mu$m, PA3: 3-10 $\mu$m, PA10: >10 $\mu$m).

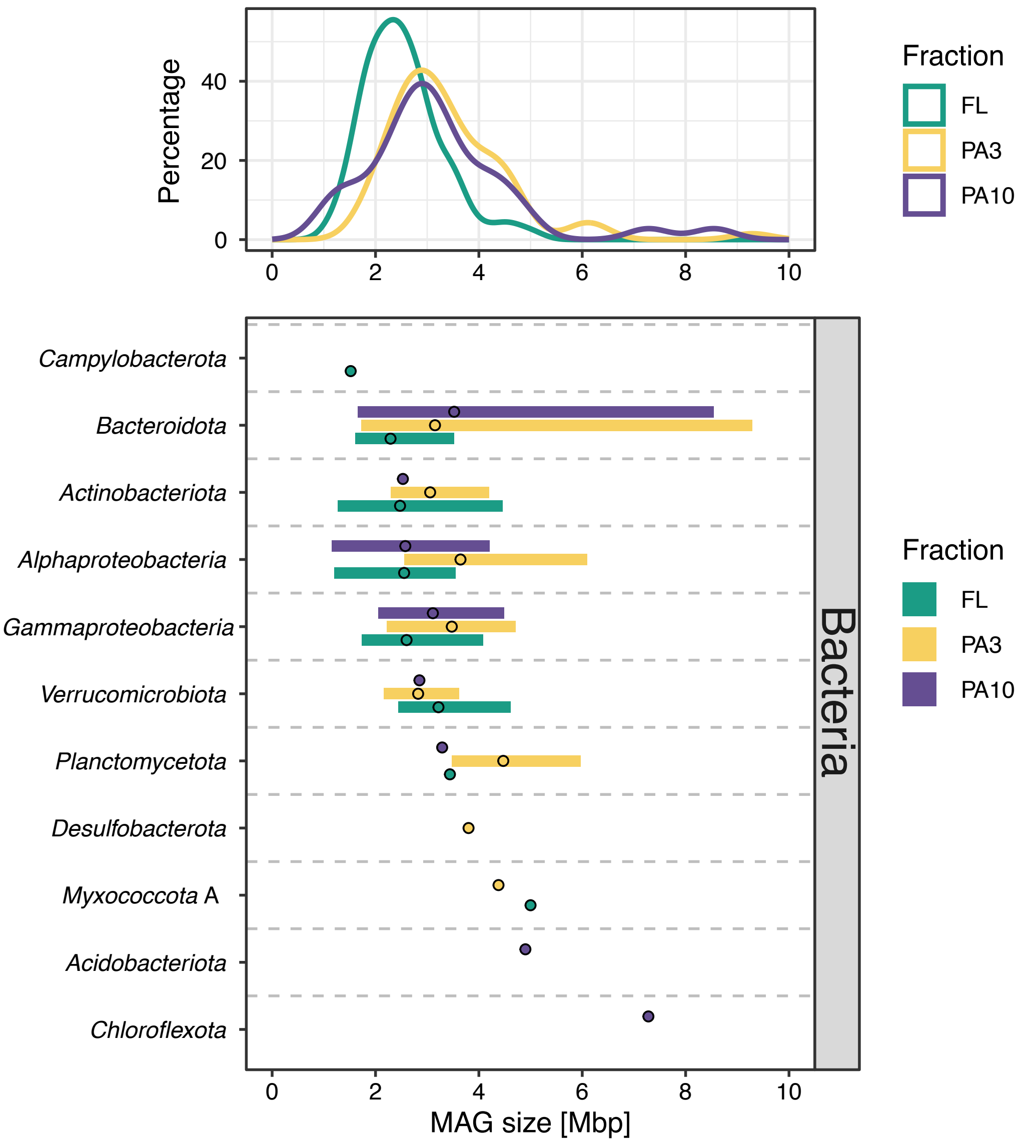


**Fig. S13. Sizes of bacterial MAGs retrieved from the 2018 FL, PA3 and PA10 metagenomes.** High-quality MAGs (n=186) were dereplicated within each fraction (FL: 0.2-3 $\mu$m, PA3: 3-10 $\mu$m, PA10: >10 $\mu$m) and used for comparison. Percentage in this context signifies the distribution of MAGs across various MAG sizes. Higher density values associated with a particular MAG size indicate a greater abundance of MAGs possessing that specific size.

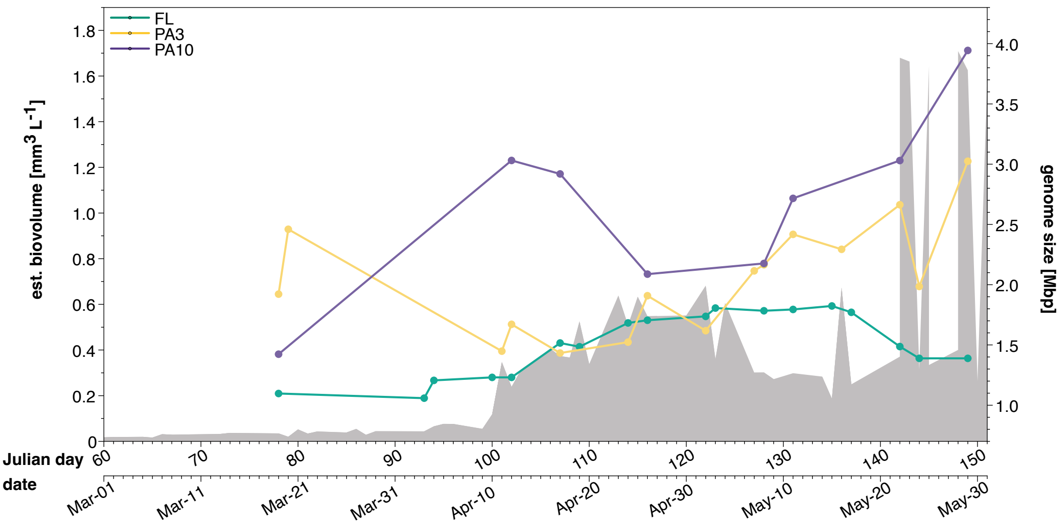


**Fig. S14. Average sizes of the ten most abundant MAGs of each sample over time (n=136).** De-replication was carried out within each fraction (FL: 0.2-3 $\mu$m, PA3: 3-10 $\mu$m, PA10: >10 $\mu$m), and MAGs belonging to the top ten in terms of abundance (as determined by MAG abundance) were specifically chosen for this analysis. The average value was derived by computing the average size of the ten most abundantly represented MAGs within the specified fraction for that particular date. The gray area in the background represents algal biovolumes including non-photosynthetic plankters (as in Fig. S1B).

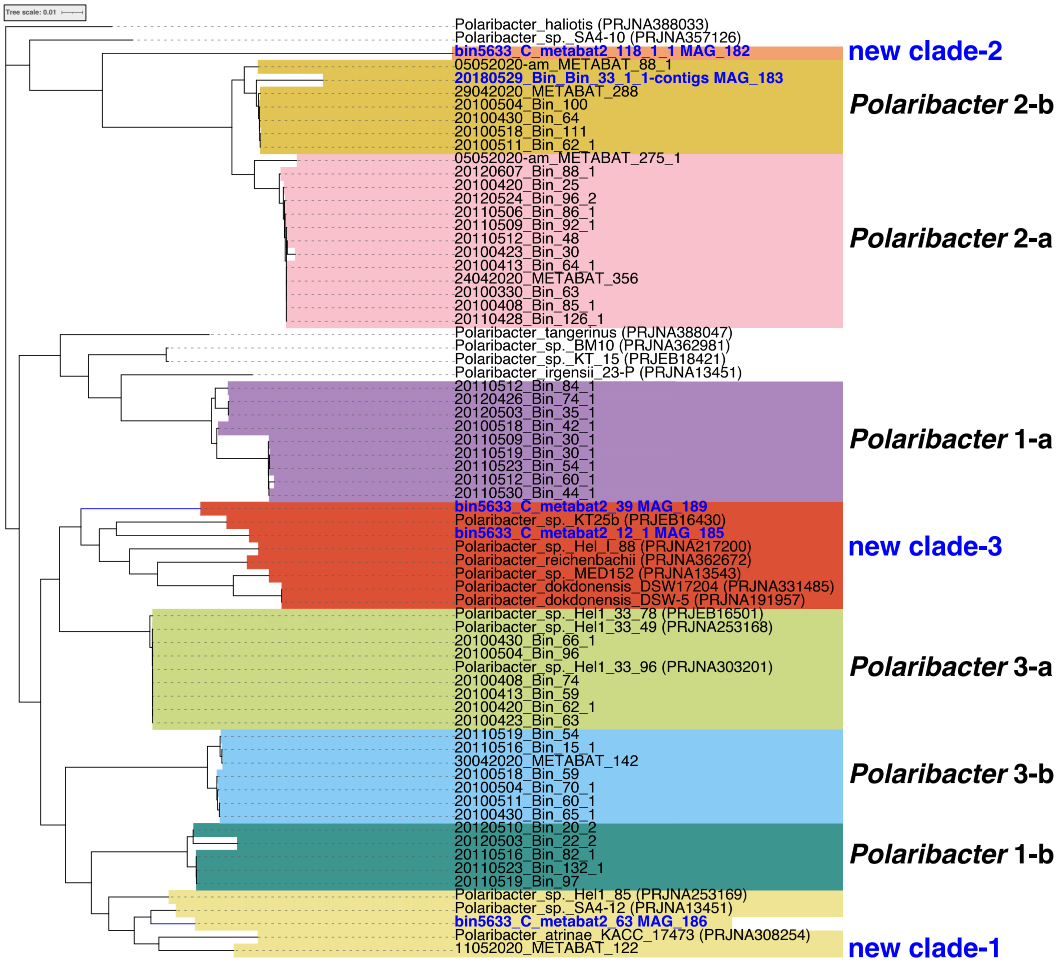


**Fig. S15. Phylogenomic tree of *Polaribacter* MAGs from Helgoland Roads.** Maximum-likelihood tree of all available *Polaribacter* MAGs from Helgoland Roads (n=52) built in anvi'o v7.1 based on protein sequences of 38 universal single-copy genes. This tree was constructed with data from a previous study as a reference [1]. MAGs from the 2018 datasets are highlighted in blue.

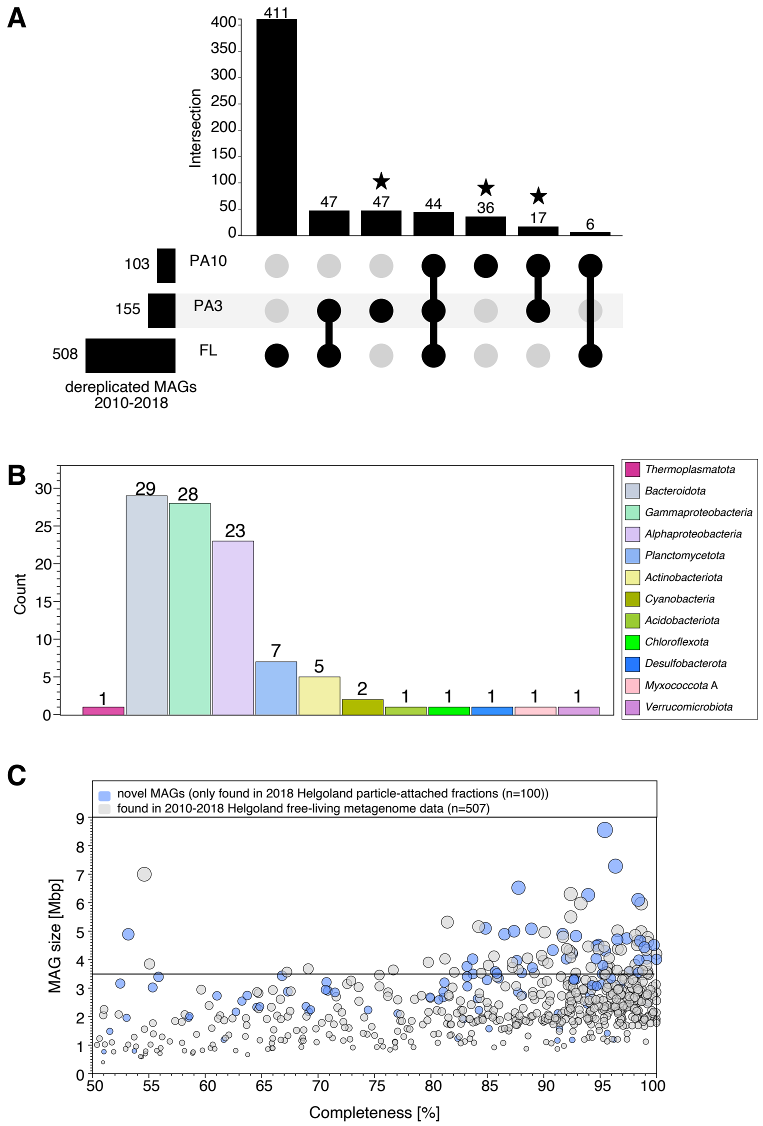


**Fig. S16. Novel MAGs from Helgoland Road samples.** We collectively analyzed MAGs from metagenomes that we sampled at Helgoland Roads during spring blooms in 2010, 2011, 2012, 2016 and 2018 (n=608). **A** MAG distribution per faction. Asterisks represent MAGs unique to the PA fractions of the 2018 data. **B** Phylum-level taxonomy of the novel 2018 PA MAGs (*Proteobacteria* are represented by the abundant *Alphaproteobacteria* and *Gammaproteobacteria* classes). **C** Sizes vs. completeness of all dereplicated MAGs (n=608). Circle area sizes correspond to MAG sizes. MAGs that were present only in the 2018 PA fractions are highlighted in blue. MAGs that were retrieved from the FL fractions of 2010, 2011, 2012, 2016 and 2018 are depicted in light gray. (FL: 0.2-3 $\mu$m, PA3: 3-10 $\mu$m, PA10: >10 $\mu$m).

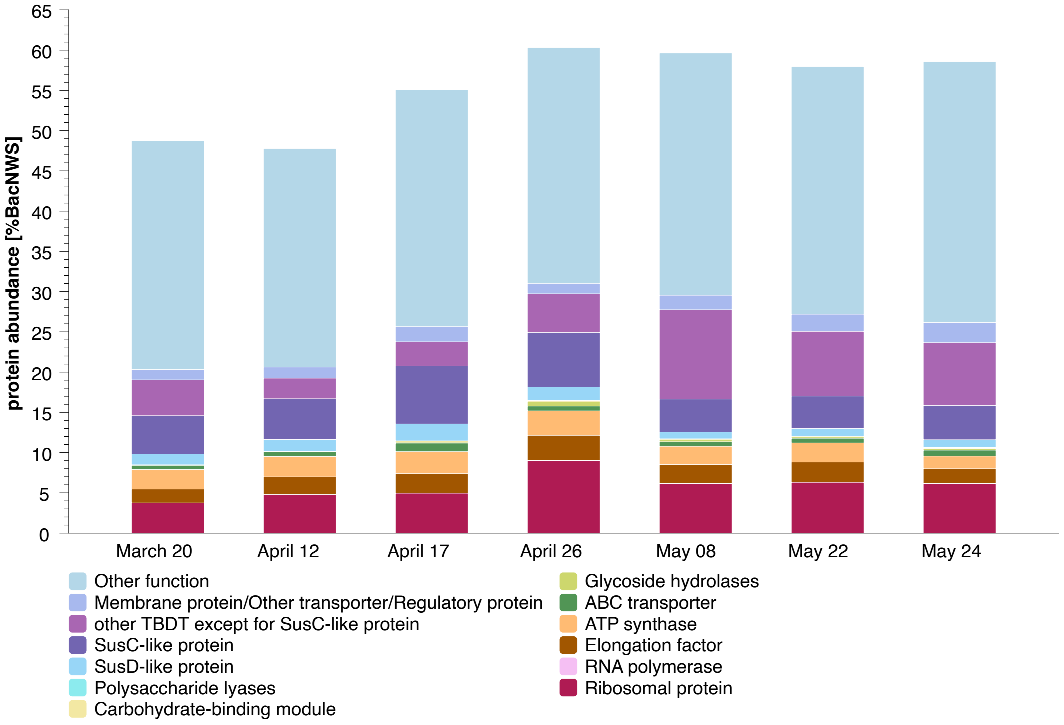


**Fig. S17. Abundant expressed proteins in the seven FL metaproteomes sampled during the 2018 spring phytoplankton bloom at Helgoland Roads.** Relative protein abundances are expressed as bacterial normalized weighted spectra (%BacNWS): We calculated the percent normalized weighted spectra (%NWS) for each protein group by dividing the 'Quantitative Value' obtained in Scaffold v4.11.1 by the sum of all quantitative values in the sample (average value for all three biological replicates for each sample). If a protein group was not identified in a replicate, we included it as '0' in the calculation. Values were normalized to 100% (%BacNWS) for comparability across samples. Noteworthy are the high proportions of SusC- and SusD-like proteins and other TonB-dependent transporters (TBDTs).

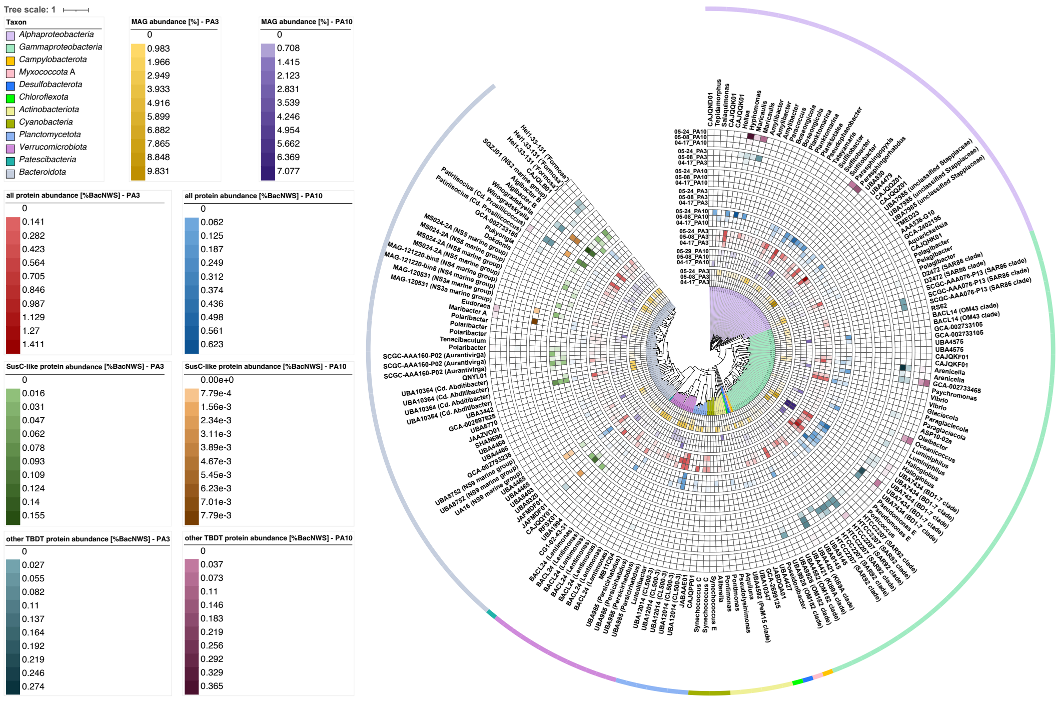


**Fig. S18. Expression of bacterial MAGs from PA samples during the 2018 spring phytoplankton bloom at Helgoland Roads.** The central maximum-likelihood tree of expressed MAGs obtained from PA3 and PA10 size fractions (n=183) was calculated in anvi'o v7.1 based on protein sequences of 38 universal single-copy genes. Predicted proteins of representative MAGs from all size fractions (FL: 0.2-3 $\mu$m, PA3: 3-10 $\mu$m, PA10: >10 $\mu$m) were used to search for the assignment of the protein fragments from proteome mass spectrometry. Circles represent (inside to outside): (i) PA3 MAG abundances, (ii) PA10 MAG abundances, (iii) PA3 MAG expression, (iv) PA10 MAG expression, (v) PA3 MAG expression of SusC-like proteins, (vi) PA10 MAG expression of SusC-like proteins, (vii) expression of TonB-dependent transporters (TBDTs) except for SusC-like proteins in PA3 MAGs, (viii) expression of TBDTs except for SusC-like proteins in PA10 MAGs, (ix) genus-level MAG taxonomy (GTDB), (x) colored arcs representing phylum-level MAG taxonomy (class for *Proteobacteria*).

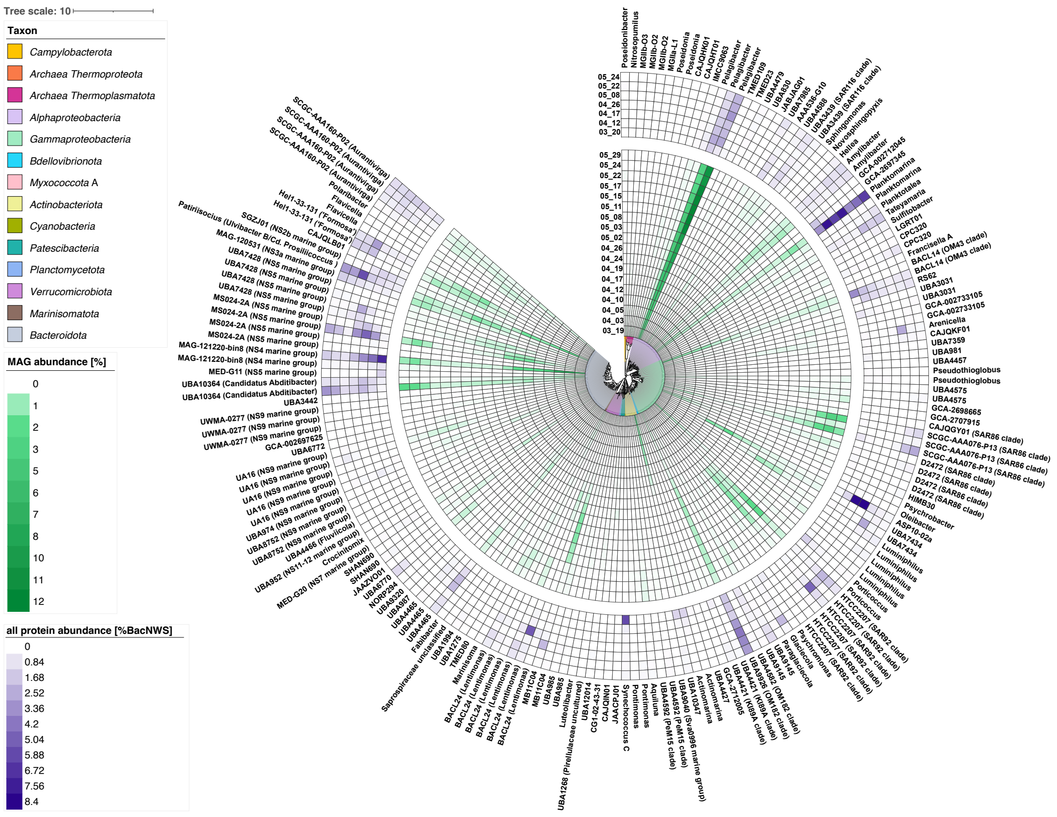


**Fig. S19. Comparison of MAG abundance and MAG expression profiles in the FL fraction.** The central maximum-likelihood tree of expressed FL (0.2-3 $\mu$m) MAGs (n=182) was calculated in anvi'o v7.1 based on protein sequences of 38 universal single-copy genes. Surrounding circles represent (inside to outside): (i) MAG abundance (green), (ii) overall expression based on summarized percentage of bacterial normalized weighted spectra (%BacNWS, purple), (iii) taxonomy as determined by GTDB r207_v2 (taxonomy in parentheses are based on Silva r138.1), (iv) phylum (class for *Proteobacteria*) level taxonomy represented by colored arcs. %BacNWS: We calculated the percent normalized weighted spectra (%NWS) for each protein group by dividing the 'Quantitative Value' obtained in Scaffold v4.11.1 by the sum of all quantitative values in the sample (average value for all three biological replicates for each sample). If a protein group was not identified in a replicate, we included it as '0' in the calculation. Values were normalized to 100% (%BacNWS) for comparability across samples.

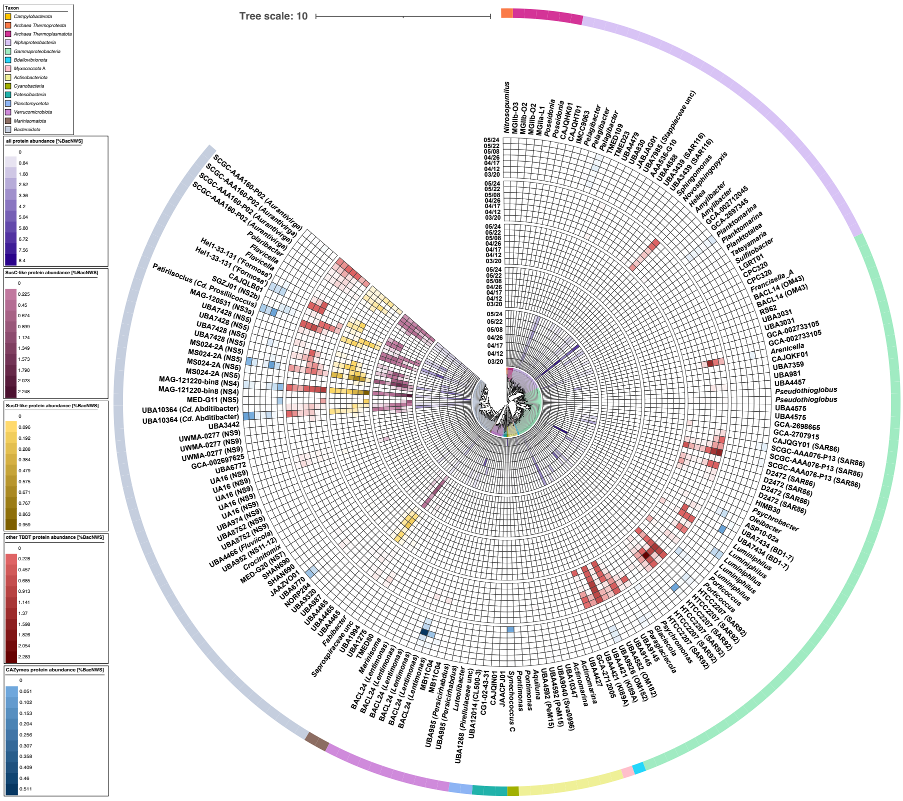


**Fig. S20. Expressed bacterial MAGs in the FL fraction.** The central maximum-likelihood tree of expressed FL (0.2-3 $\mu$m) MAGs (n=182) was calculated in anvio v7.1 based on protein sequences of 38 universal single-copy genes. Surrounding circles represent (inside to outside): (i) overall expression based on the summarized percentage of bacterial normalized weighted spectra (%BacNWS), (ii) expression of SusC-like proteins, (iii) expression of SusD-like proteins, (iv) expression of TonB-dependent transporters (TBDTs) except for SusC-like proteins, (v) expression of degradative CAZymes (GH, CE, PL), (vi) taxonomy as determined by GTDB r207_v2 (taxonomy in parentheses are based on Silva r138.1), (vii) phylum (class for *Proteobacteria*) level taxonomy represented by colored arcs. %BacNWS: We calculated the percent normalized weighted spectra (%NWS) for each protein group by dividing the 'Quantitative Value' obtained in Scaffold v4.11.1 by the sum of all quantitative values in the sample (average value for all three biological replicates for each sample). If a protein group was not identified in a replicate, we included it as '0' in the calculation. Values were normalized to 100% (%BacNWS) for comparability across samples.

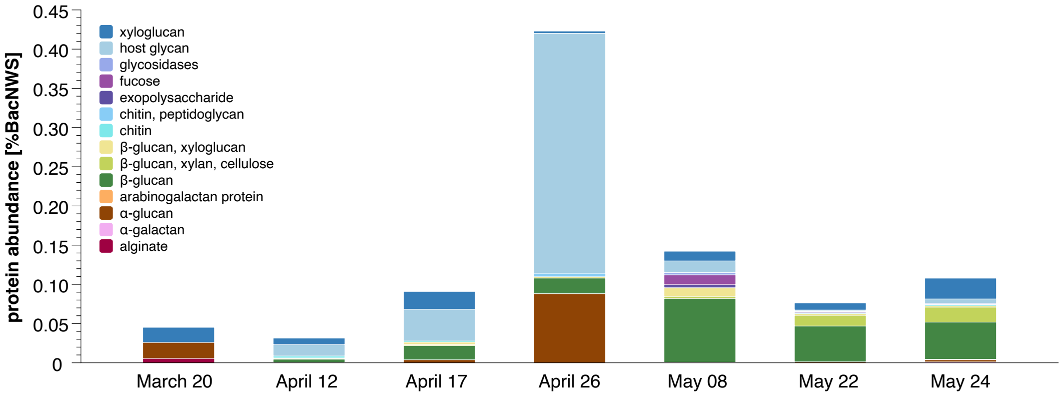


**Fig. S21. Abundant expressed CAZymes in the seven FL metaproteomes sampled during the 2018 spring phytoplankton bloom at Helgoland Roads.** Relative protein abundances are expressed as bacterial normalized weighted spectra (%BacNWS). Corresponding GHs, PLs and CEs included in CAZyme analyses were annotated with dbCAN3-sub and are listed in Table S12 in Additional file 1. %BacNWS: We calculated the percent normalized weighted spectra (%NWS) for each protein group by dividing the 'Quantitative Value' obtained in Scaffold v4.11.1 by the sum of all quantitative values in the sample (average value for all three biological replicates for each sample). If a protein group was not identified in a replicate, we included it as '0' in the calculation. Values were normalized to 100% (%BacNWS) for comparability across samples.

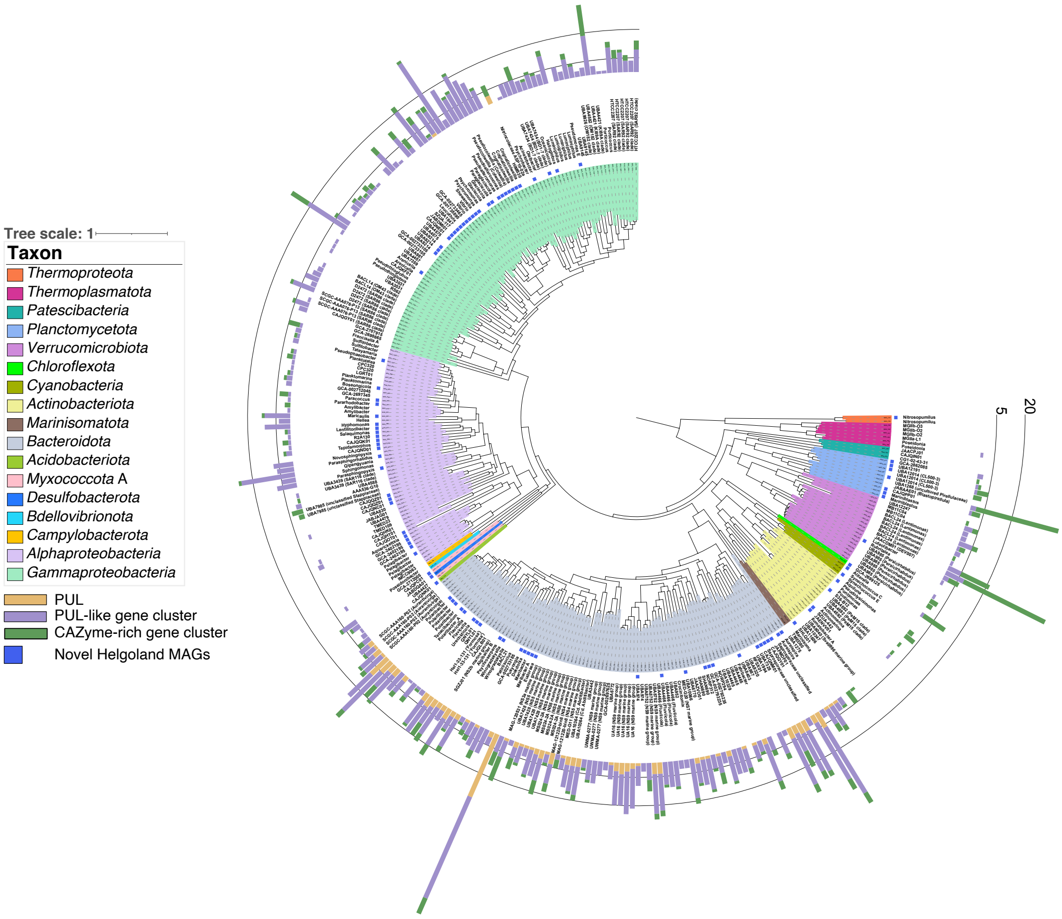


**Fig. S22. Numbers of PULs, PUL-like and CAZyme-rich gene clusters of dereplicated MAGs.** The central maximum-likelihood tree of 305 de-replicated bacterial MAGs was calculated in anvi'o v7.1 based on protein sequences of 38 universal single-copy genes. Surrounding circles represent (inside to outside): (i) phylum-level taxonomy (class for *Proteobacteria*), (ii) novelty with respect to our previous studies on Helgoland Roads (solid blue squares), (iii) stacked bar plots representing PUL counts, PUL-like clusters and CAZyme-rich gene clusters with black lines representing scales of 5 and 20.

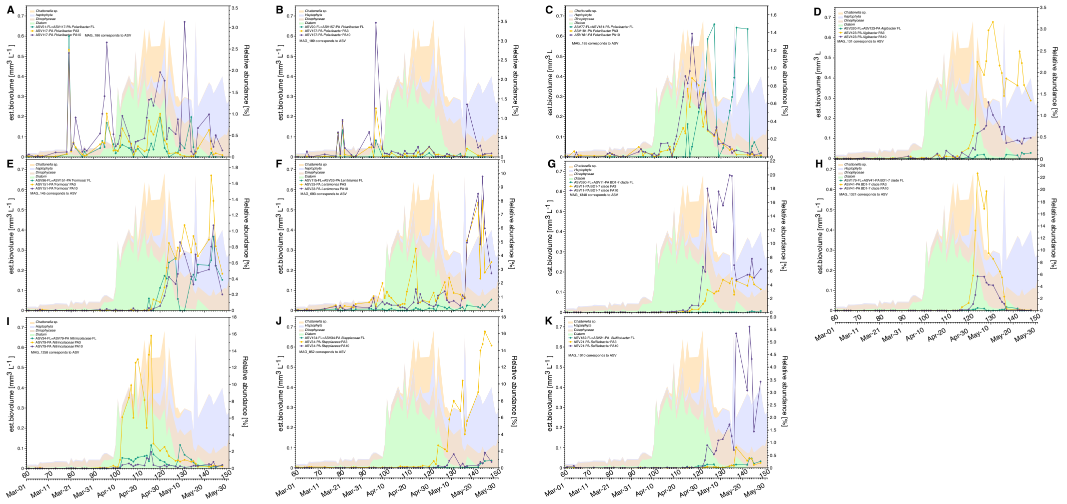

**Fig. S23. Relative abundance of ASVs corresponding to MAGs.** The four main algal groups are represented as colored areas, and abundant ASVs are represented by line graphs. Identical ASVs in different fractions are indicated in the legend (e.g., "ASV51-FL=ASV117-PA”). The MAG that corresponds to the ASVs in each panel is indicated (e.g., “MAG_186 corresponds to ASV”).

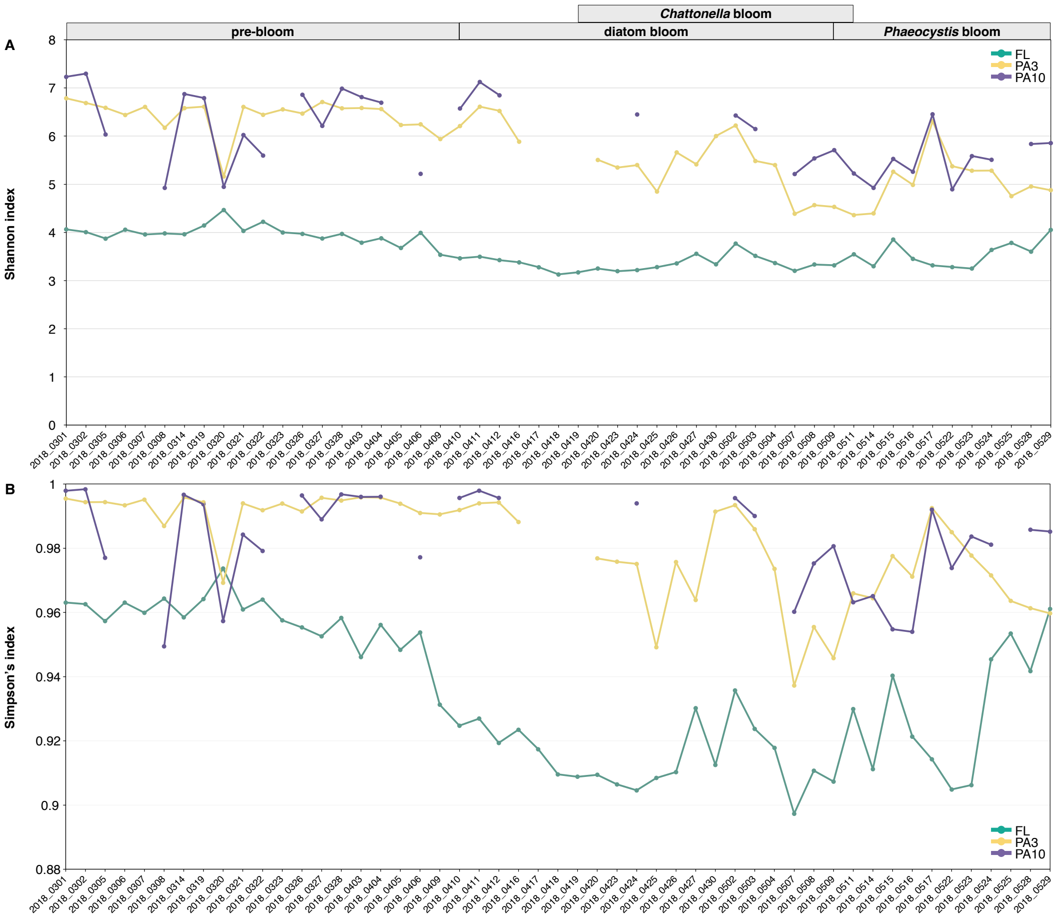


**Fig. S24. Sample diversity indices based on 16S rRNA gene ASVs with rarefaction.** Sample diversities of the bacterial communities with rarefaction of all size fractions (FL: 0.2-3 $\mu$m, PA3: 3-10 $\mu$m, PA10: >10 $\mu$m) were assessed by **A** Shannon and **B** Simpson's diversity indices. 10,000 ASVs were randomly selected. The "Rarefy" function in the R package GuniFrac [2] was used for rarefaction. Colors correspond to sample fractions.

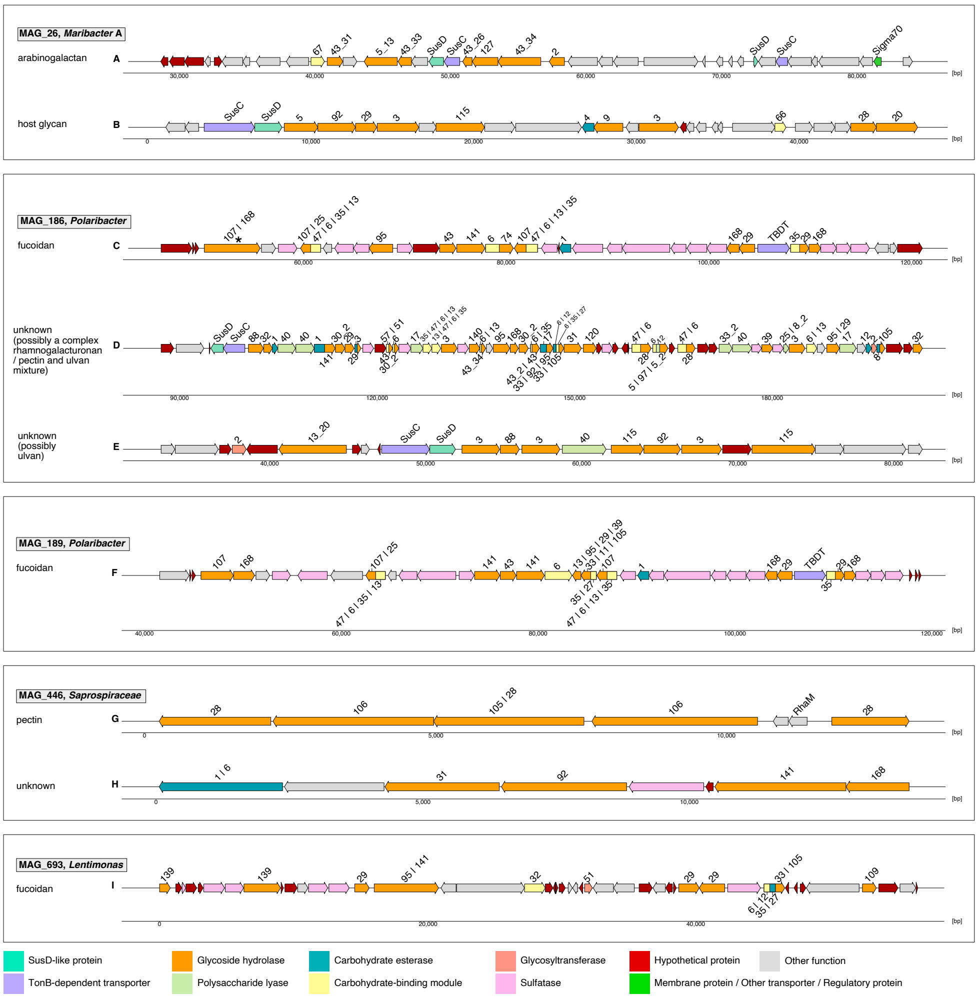


**Fig. S25. Example polysaccharide utilization loci.** MAG affiliations for each contig are indicated. Colors of gene representations indicate gene types and corresponding numbers indicate CAZyme family associations. Substrates were predicted with dbCAN3-sub. Some PULs are incomplete. A possible frameshift is marked by an asterisk.

## References


1. Avcı B, Krüger K, Fuchs BM, Teeling H, Amann RI. Polysaccharide niche partitioning of distinct *Polaribacter* clades during North Sea spring algal blooms. ISME J. 2020;14(6):1369-83.
2. Chen J, Chen MJ. Package ‘GUniFrac’. The Comprehensive R Archive Network (CRAN). 2018.
