# Supplementary material for: Particle-attached bacteria act as gatekeepers in the decomposition of complex phytoplankton polysaccharides
Source: Microbiome. 2024 Feb 20;12:32. doi: 10.1186/s40168-024-01757-5 (PMC10877868; doi:10.1186/s40168-024-01757-5)
Supplement: Supplementary file 4 — Additional file 3: Supplementary text. [file 40168_2024_1757_MOESM3_ESM.docx]

***Microbiome - Additional file 1***

**Particle-attached bacteria act as gatekeepers in the decomposition of complex phytoplankton polysaccharides**

Feng-Qing Wang^1^, Daniel Bartosik^2,3†^, Chandni Sidhu^1†^, Robin Siebers^4^, De-Chen Lu^1,5^, Anke Trautwein-Schult^4^, Dörte Becher^4^, Bruno Huettel^6^, Johannes Rick^7^, Inga V. Kirstein^7^, Karen H. Wiltshire^7^, Thomas Schweder^2,3^, Bernhard M. Fuchs^1^, Mia M. Bengtsson^4^*, Hanno Teeling^1^*, Rudolf I. Amann^1^*

^1^ Max Planck Institute for Marine Microbiology, Celsiusstraße 1, 28359 Bremen, Germany

^2^ Institute of Pharmacy, University of Greifswald, Felix-Hausdorff-Straße 3, 17489 Greifswald, Germany

^3^ Institute of Marine Biotechnology, Walther-Rathenau-Straße 49a, 17489 Greifswald, Germany

^4^ Institute of Microbiology, University of Greifswald, Felix-Hausdorff-Straße 8, 17489 Greifswald, Germany

^5^ Marine College, Shandong University, Weihai 264209, China

^6^ Max Planck Genome Centre Cologne, Carl von Linné-Weg 10, 50829 Köln, Germany

^7^ Alfred Wegener Institute for Polar and Marine Research, Biologische Anstalt Helgoland, 27483 Helgoland, Germany

^†^ These authors contributed equally to this study.

* Corresponding authors:

Rudolf I. Amann, Max Planck Institute for Marine Microbiology, Celsiusstraße 1, 28359 Bremen, e-mail: [ramann@mpi-bremen.de](mailto:ramann@mpi-bremen.de), phone: +49 421 2028 9300

Hanno Teeling, Max Planck Institute for Marine Microbiology, Celsiusstraße 1, 28359 Bremen, e-mail: [hteeling@mpi-bremen.de](mailto:hteeling@mpi-bremen.de), phone: +49 421 2028 9760

Mia M. Bengtsson, University of Greifswald, Felix Hausdorff-Straße 8, 17489 Greifswald, Germany, e-mail: [mia.bengtsson@uni-greifswald.de](mailto:mia.bengtsson@uni-greifswald.de), phone: +49 383 4420 4212

E-mail addresses and telephone numbers of all authors:

Feng-Qing Wang [fwang@mpi-bremen.de](mailto:fwang@mpi-bremen.de) +49 421 2028 9390

Daniel Bartosik [daniel.bartosik@uni-greifswald.de](mailto:daniel.bartosik@uni-greifswald.de) +49 3834 420 4918

Chandni Sidhu [csidhu@mpi-bremen.de](mailto:csidhu@mpi-bremen.de) +49 421 2028 9582

Robin Siebers [siebersr@vodafonemail.de](mailto:robin.siebers@uni-greifswald.de) +49 3834 420 5931

De-Chen Lu 202267000015@sdu.edu.cn +86 0631 5688303

Anke Trautwein-Schult anke.trautwein-schult@uni-greifswald.de +49 3834 420 5922

Dörte Becher [dbecher@uni-greifswald.de](mailto:dbecher@uni-greifswald.de) +49 3834 420 5903

Bruno Hüttel [huettel@mpipz.mpg.de](mailto:huettel@mpipz.mpg.de) +49 221 5062 828

Johannes Rick [johannes.rick@awi.de](mailto:johannes.rick@awi.de) +49 4651 956 4220

Inga V. Kirstein [inga.kirstein@awi.de](mailto:inga.kirstein@awi.de) +49 4725 819 3153

Karen H. Wiltshire [Karen.Wiltshire@awi.de](mailto:Karen.Wiltshire@awi.de) +49 4651 956 4112

Thomas Schweder [schweder](mailto:schweder@uni-greifswald.de)[@uni-greifswald.de](mailto:scheder@uni-greifswald.de) +49 3834 420 4212

Bernhard M. Fuchs [bfuchs@mpi-bremen.de](mailto:bfuchs@mpi-bremen.de) +49 421 2028 9350

Maria M. Bengtsson [mia.bengtsson@uni-greifswald.de](mailto:mia.bengtsson@uni-greifswald.de) +49 421 2028 9390

Hanno Teeling [hteeling@mpi-bremen.de](mailto:hteeling@mpi-bremen.de) +49 421 2028 9760

Rudolf I. Amann [ramann@mpi-bremen.de](mailto:ramann@mpi-bremen.de) +49 421 2028 9300

**This file includes:**

Supplementary Results

Supplementary Materials and Methods

Supplementary Code Availability

Supplementary Database Availability

Supplementary References

**Supplementary Results**

*Phytoplankton bloom characteristics and physicochemical data*

During the sampling period, surface water temperatures increased from 2.7 to 12 °C, and the salinity ranged from 31.7 to 34.2 (Fig. S3D in Additional file 2). The concentrations of silicate and nitrate decreased notably with the onset of the main bloom phase. Silicate was almost completely consumed at the peak of the diatom bloom (Fig. S3C in Additional file 2), because diatoms consume silicate to build their frustules (epitheca and hyptheca). In contrast, the decrease in nitrate concentrations was slower and reached its lowest values during the late bloom stage (Fig. S3B in Additional file 2). Since nitrate is the most important nitrogen source for phytoplankton growth, it can be assumed that the bloom went into terminal decline shortly after the end of the sampled time period.

*Weather data*

Rain was rather sparse during the sampling period. April 29 had the highest precipitation with 34 L/m^2^ (Table S4 in Additional file 1), which did not have any detectable effect on salinity or any other of the data. In contrast, the continuous increase in sunshine duration in May notably promoted the phytoplankton bloom (Table S4 in Additional file 1).

*Diversity analysis of FL and PA bacterial communities*

The 16S rRNA gene amplicon data analysis discussed in the main manuscript was obtained with different primer sets for FL and PA bacteria (see Materials and Methods in the main manuscript). Furthermore, an uneven number of sequences was obtained for FL and PA fractions even after normalization due to high proportions of chloroplast sequences in PA data (Table S5 in Additional file 1). We hence conducted diversity analyses with rarefaction to account for different sample sizes. However, the results were similar to analyses without rarefaction (Figs. 2A-S24 in Additional file 2) but led to a loss of some of the undersampled PA10 points in time. We therefore included the analyses without rarefaction in the main manuscript (Fig. 2), even more so, since rarefaction is a debated process that may induce its own bias [1, 2].

As a second test, we compared the 16S rRNA gene amplicon data with independent read-based taxonomic classifications (see supplementary Materials and Methods in this document). These data supported the 16S rRNA gene-based analyses, as they also revealed a substantially higher diversity of PA3 and PA10 communities compared to FL communities (Fig. S8B-C in Additional file 2), as well as more similar community compositions between PA3 and PA10 fractions compared to FL fractions (Fig. S8A in Additional File 2). The latter was also supported by the stark differences in the taxonomic affiliations of MAGs that were obtained from PA and FL communities. These results combined indicate that influences of different primer pairs and uneven data sizes were negligible compared to the pronounced differences in the most abundant taxa that we analyzed. Still, we do acknowledge the potential influence that the use of different primers during 16S rRNA gene sequencing may have for the analysis of less abundant or even rare taxa, for which in particular the PA10 data would benefit from additional sequencing depth.

*Composition of FL and PA bacterial communities*

After removal of chloroplast and mitochondria sequences from merged 16S rRNA gene amplicon data, 2,546 and 18,879 bacterial ASVs were retained for the FL and PA fractions, respectively. FL ASVs represented 27 phyla, 43 classes, 126 orders, 153 families and 263 genera. PA ASVs represented 68 phyla, 149 classes, 361 orders, 498 families and 1,151 genera.

For each size fraction, we investigated ASVs present in at least 80% of the samples with a relative abundance exceeding 0.1% in at least one sample. Such common ASVs represented 11.7-85.2% (FL, 45 ASVs), 47.3-77.1% (PA3, 242 ASVs), and 14.4-84.2% (PA10, 127 ASVs), respectively (Table S5 in Additional file 1).

In FL communities, members of the SAR11 clade II, SAR116 clade, unclassified *Rhodobacteraceae*, OM43 clade, NS4 marine group, and *Cd.* Actinomarina were abundant during the pre-bloom and early diatom-dominated main bloom phases, but their abundances decreased during the diatom bloom’s decline and the late surge in haptophytes (Fig. S7A in Additional file 2). Similar trends were observed for members of the SAR11 clade II, SAR116 clade and NS4 marine group with lower relative abundances in both PA fractions. Contrary to the FL fraction, Cd. Actinomarina ASVs were hardly detectable in PA fractions (FL: 0.1-10.3%, PA3: 0-0.4%, PA10: 0-1.2%).

*ASV level composition of FL and PA bacterial communities*

ASV level taxonomic assignments that are discussed in this section are summarized in Table S5 in Additional file 1.

*SAR11 clade Ia*

In the FL fraction, there were two very abundant ASVs affiliating with the SAR11 clade Ia (ASV_1, ASV_2). ASV_1 slightly declined during the late diatom and *Phaeocystis* bloom phases, whereas ASV_2 was more abundant during these bloom phases. Notably, no SAR11 clade Ia ASV with the same dynamic change was found in PA fractions. There were also ASVs with high abundances only during the pre-bloom, which declined (relatively) with the onset of the bloom. These ASVs were found in both, FL and PA fractions (ASV_25 and ASV_33 in the FL, and ASV_52 in the PA fractions).

*Planktomarina*

The genus *Planktomarina* was dominated by a single ASV in all fractions (FL ASV_3 was 100% similar to PA ASV_15).

*Amylibacter*

Two *Amylibacter* ASVs (ASV_7, ASV_17) had higher abundance in the FL fraction, with ASV_7 exhibiting higher abundance throughout all sampled points in time, and ASV_17 with higher abundance only during the *Phaeocystis* bloom. The latter ASV was not detected in the PA fractions.

BD1-7 clade

There were two dominant BD1-7 clade ASVs in the PA fractions. ASV_41 was more abundant in the PA3 (0-22.7%), while ASV_11 was more abundant in the PA10 (0-20.0%) fraction. Both were abundant during the late diatom and *Phaeocystis* bloom phases.

*Ulvibacter*

A single ASV (ASV_6) dominated *Ulvibacter* in the FL size fraction (0-17.6%), with highest relative abundances during the late diatom and early *Phaeocystis* bloom phases. In PA fractions, ASV_46 (100% similarity with FL ASV_6) was dominant in PA3, but with lower relative abundance (0-6.6%).

*unclassified Nitrincolaceae*

Unclassified *Nitrincolaceae* were dominated by a single ASV (ASV_79) in PA3 fractions with relative abundances of up to 15.8%. This clade was only abundant during the diatom bloom phase.

*unclassified Stappiaceae*

Unclassified *Stappiaceae* were dominated by ASV_34 in PA3 fractions with relative abundances of 0-16.3%. The highest relative abundances were observed during the *Phaeocystis* bloom phase.

*Persicirhabdus (Verrucomicrobiota)*

Two ASVs (ASV_29, ASV_32) dominated *Persicirhabdus* in PA fractions. Both were more abundant in the PA3 (0.1-18% and 0.02-7.8%) than in the PA10 fraction (0-6% and 0-2.8%).

*CL500-3 (Planktomycetota)*

A single ASV (ASV_99) dominated the CL500-3 clade. It reached up to 8.6% relative abundance in PA3 fractions during the late diatom and early *Phaeocystis* bloom phases.

*Sulfitobacter*

*Sulfitobacter* was represented by a single dominant ASV (ASV_21) in PA10 size fractions with up to 5.6% relative abundance, specifically during the *Phaeocystis* bloom phase.

‘Formosa’

In the FL size fractions, ‘Formosa’ was represented by two ASVs with similar relative abundances (ASV_79: 0-1.2%, ASV_96: 0-0.9%), in particular during the late diatom and early *Phaeocystis* bloom phases. Additional distinct two ‘Formosa’ ASVs were present in the PA3 fractions (ASV_271: 0-1.0%, ASV_151: 0-1.7%).

*Algibacter*

In the genus *Algibacter* a single ASV (ASV_123) dominated. It was present in PA3 fractions with up to 3.2% relative abundance, specifically during the late diatom and early *Phaeocystis* bloom phases.

*Polaribacter*

The *Polaribacter* genus was dominated by five ASVs. ASV_117, ASV_218, ASV_26 and ASV_181 were abundant in both PA3 (0-2.5%, 0-2.2%, 0-2.0% and 0-0.9%, respectively) and PA10 fractions (0-3.1%, 0-1.9%, 0-2.0% and 0-1.4%, respectively), whereas ASV_1063 was detected in PA10 (0-1.1%) but barely in PA3 (0-0.4%) fractions. All five *Polaribacter* ASVs exhibited their highest relative abundances during the diatom bloom.

*Glaciecola*

The genus *Glaciecola* was dominated by a single ASV (ASV_381) with a relative abundance of 0-1.8% in the PA10 size fraction during the late diatom and *Phaeocystis* bloom phases.

*Reconstruction of MAGs*

MAGs with an ANI ≥95% were combined to species-level clusters and subsequently taxonomically assigned. *Bacteroidota* (96 clusters, 466 MAGs), *Gammaproteobacteria* (84 clusters, 473 MAGs) and *Alphaproteobacteria* (57 clusters, 273 MAGs) represented the most abundant taxa, followed by *Actinobacteriota* (17 clusters, 75 MAGs), *Verrucomicrobiota* (17 clusters, 100 MAGs), *Planctomycetota* (10 clusters, 42 MAGs), *Patescibacteria* (3 clusters, 14 MAGs), *Cyanobacteria* (3 clusters, 10 MAGs), *Myxococcota* (2 clusters, 13 MAGs), *Marinisomatota* (2 clusters, 3 MAGs), *Desulfobacterota* (1 cluster,6 MAGs), *Bdellovibrionota* (1 cluster, 3 MAGs), *Chloroflexota* (1 cluster, 2 MAGs), *Acidobacteriota* (1 cluster, 1 MAG), *Campylobacterota* (1 cluster, 1 MAG), *Thermoplasmatota* (Archaea, 6 clusters, 23 MAGs) and *Thermoproteota* (Archaea, 2 clusters, 4 MAGs).

*152 abundant PA MAGs*

Based on ASV and MAG abundance data, we categorized MAGs into those that were most abundant in either FL or PA communities and those that were abundant in both. A total 152 MAGs of those that could be taxonomically were most abundant in PA communities. These MAGs comprised *Bacteroidota* (45 MAGs), *Gammaproteobacteria* (37 MAGs), *Alphaproteobacteria* (29 MAGs), *Verrucomicrobiota* (11 MAGs), *Actinobacteriota* (10 MAGs), *Planctomycetota* (10 MAGs), *Cyanobacteria* (3 MAGs), *Myxococcota* A (2 MAGs), *Acidobacteriota* (1 MAG), *Bdellovibrionota* (1 MAG), *Chloroflexota* (1 MAG), *Desulfobacterota* (1 MAG) and *Thermoproteota* (1 MAG).

*MAG sizes as a function of size fraction, taxonomy and time*

Average HQ MAGs sizes were larger in PA than in FL communities (Fig. S13 in Additional file 2), most notably for *Bacteroidota* and *Gammaproteobacteria*. Exceptions were *Alphaproteobacteria* and *Planctomycetota* with similar sizes in FL and PA10, but larger MAGs in PA3 communities, and *Verrucomicrobiota* with larger average MAG sizes in FL communities. The largest MAG was found in the *Bacteroidota* (*Saprospiraceae* MAG_98, 8.6 Mbp), followed by a MAG from the *Chloroflexota* (*Promineofilaceae* MAG_535, 7.3 Mbp), both from PA10 samples. However, on overall, median MAG sizes of FL community members of all phyla except for *Myxococcota* A (5.0 Mbp) were below the average aquatic genome size of 3.1 Mbp [3].

We observed an increase in size within the FL communities during the diatom bloom (Fig. S14 in Additional file 2). This trend reversed amidst the *Phaeocystis* bloom phase, likely due to increased abundances of SAR86, a clade characterized by relatively small genome sizes (0.7 to 1.5 Mbp in our data). Different trends were observed for both PA communities. Average MAG sizes of PA3 communities increased during the diatom and *Phaeocystis* bloom phases, whereas sizes of PA10 communities first decreased during the diatom bloom and increased during the *Phaeocystis* bloom phases. In general, fluctuations over time were more pronounced for both PA communities, likely due to the natural variability of particulate matter on the filters themselves.

*Categories of expressed proteins of FL communities*

Of the combined detected proteins in the metaproteomes of FL bacteria, 15,906 (36.4%) could be assigned to 177 of the 182 dereplicated FL MAGs. Annotated functions comprised 2,565 ribosomal proteins, 718 ABC transporters, 704 TBDTs including 103 SusC-like proteins, 347 CAZymes and 80 SusD-like proteins (Fig. S17 in Additional file 2).

*Expression of SusC-like proteins and other TBDTs*

In the 177 MAGs with mapped metaproteome data, we identified 168 SusC-like proteins and 2,071 other TBDTs, of which 103 SusC-like proteins and 601 other TBDTs were expressed. Combined SusC-like relative protein abundances peaked on April 17 (7.2%) and April 26 (6.8%) during the diatom-bloom, then decreased on May 8 (4.1%), May 22 (4.0%), and May 24 (4.3%), which was even lower than the expressed SusC-like proteins during the pre-bloom on the March 20 (4.8%) and during the onset of the diatom-bloom on the April 12 (5.1%). SusD-like proteins exhibited a similar expression pattern but with lower relative protein abundances (0-0.06%). The relative expression of other TBDTs decreased from March 20 (4.6%) to April 12 (2.6%) and April 17 (3.0%). Afterwards, TBDT expression started to increase again until pre-bloom levels on April 26 (4.8%), and then continued to its highest relative expression on May 8 (11.1%). After this peak, values decreased again (May 22: 8.0%, May 24: 7.8%) but stayed above pre-bloom levels.

In *Bacteroidota*, SusC-like protein expression corresponded well with overall MAG abundances (Fig. S20 in Additional file 2) with the exception of *Cd.* Abditibacter (MAG_401) which despite high overall relative abundance featured no detectable SusC-like protein expression. The expression of transporter proteins differed during different bloom periods (Fig. S17 in Additional file 2). During the diatom-dominated bloom phase on April 12, 17 and 26, SusC-like proteins were mainly expressed by the NS5 marine group, UBA4465 and UBA7428. On May 8, expressed SusC-like proteins were more from *Aurantivirga*, *Cd.* Prosiliicoccus, and ‘Formosa’. SusC-like proteins from the NS3a marine group and *Cd.* Abditibacter expressed more on May 22 and 24. The shifts in other TBDT expression profiles were more pronounced, with a significant increase on May 8, which was dominated by other TBDTs from the SAR92 clade, *Arenicella* and *Cd.* Prosiliicoccus. On May 22 and 24, the dominating MAGs with expressed TBDT shifted to the SAR86 clade.

High SusC-like protein expression in *Flavobacteriaceae* included MAG_146 (NS2b marine group), MAG_142 (‘Formosa’), MAG_131 (*Winogradskyella*), MAG_104 (*Algibacter* B), MAG_98 (*Dokdonia*), MAG_41 (NS5 marine group), MAG_183 (*Tenacibaculum*), MAG_195 (*Polaribacter*), MAG_176, MAG_194 and MAG_196 (*Aurantivirga*), and in *Saprospiraceae* MAG_446 (JAFMDF) and MAG_452 (RFSX01). In addition, MAG_26 (*Eudoraea)* featured the highest SusC-like protein expression (0.008%) in PA10 on May 24. MAG_434 (UBA4465) exhibited high SusC-like protein expression on May 8 and May 24 in PA10 and May 24 in PA3.

MAG_24 (NS4 marine group) had some expression of other TBDTs in PA10 on May 24. MAG_680 (*Lentimonas*), and MAG_585 and MAG_607 (*Planctomycetota;* UBA12014) expressed TBDTs in PA10 and PA3 fractions. Further TBDT expression was mostly observed in *Alpha-* and *Gammaproteobacteria*, for example in MAG_1474 (UBA4421 / KI89A clade), MAG_1396, MAG_1406 and MAG_1413 (SAR92), MAG_1278 (*Oceanicoccus*), MAG_1201 (GCA-002733465), MAG_1093 (*Arenicella*), MAG_1075 (SAR86), MAG_865 (*Parasphingopyxis*), MAG_869 (*Parasphingorhabdus*), MAG_887 (*Maricaulis*) and MAG_886 (*Hyphomonas*).

MAG_1005 (*Sulfitobacter*) expressed periplasmic and bacterial extracellular solute binding proteins on May 24 in both PA3 and PA10 communities. MAG_1018 (*Tateyamaria*) expressed porins and bacterial extracellular solute binding proteins on May 8 and 24 in both PA3 and PA10 communities (Fig. S18 in Additional file 2).

*Expression of catabolic CAZymes in FL communities*

GHs showed the highest relative expression on the April 26 (0.5%), which included the highest relative expression of α-fucose-containing degradation genes (GH95 and GH29), α-mannan-containing (GH92) and α-glucan-containing (GH13_9|CBM48, GH13_3 and GH13_14) substrates (Fig. S20 in Additional file 2). Expressed GH29 was detected in MAGs of the NS5 marine group and BACL24 (*Lentimonas*). Expressed GH95 and GH92 were only detected in a BACL24 (*Lentimonas*) MAG. Laminarin-targeting GH16_3 and GH149 exhibited particularly high relative protein abundances on May 8 (0.07% each), May 22 (0.04%, 0.03%) and May 24 (0.04% each) when compared to the other four metaproteome sample dates. GH16_3 and GH149 were expressed in several MAGs, not only in *Bacteroidota* but also in *Gammaproteobacteria*. The relative abundance of these MAGs increased during the late diatom and *Phaeocystis* bloom phases. Expressed GH33 was only detected on May 22 (0.02%) and May 24 (0.03%), while expressed GH74 was detected across all samples (0.01-0.1%). Expressed PL22 was also detected on all the samples except for March 20.

*MAG analyses highlight distinct polysaccharide degradation potentials in abundant FL and PA communities*

We linked MAGs with 16S rRNA gene amplicon data to leverage the high temporal resolution amplicon data to uncover variations in MAG abundances (Table S10 in Additional file 1, Fig. S23 in Additional file 2), for which we selected the 71 most abundant MAGs for in-depth PUL analysis. Nine of these harbored 40 or more CAZyme genes, all of which were prevalent in PA communities (Fig. 7). A holistic summary of the main results is provided in the discussion of the main manuscript. Here, we describe the key CAZyme genes and inferred substrates of the most prominent MAGs.

*Order* Flavobacteriales

*Maribacter* (2 MAGs): MAG_26 was abundant in PA10 communities and showed no clear abundance trend, except for a decrease during the *Phaeocystis* bloom. It possessed 24 predicted PULs and 133 CAZyme genes assigned to 18 substrates, including five PULs predicted to target complex host glycans (e.g., Fig. S25). Additional PULs were predicted to target α-glucans, β-glucans, xylans, xyloglucans, cellulose, and arabinogalactans (Fig. S25). In contrast, *Maribacter* MAG_25 featured only a single fructan (GH32) PUL, showcasing a pronounced intra-genus-level variation in PUL repertoires.

*Polaribacter* (5 MAGs including *Polaribacter* A): Three of the five *Polaribacter* MAGs correlated with distinct ASVs (Fig. S23A-C in Additional file 2). MAG_185 exhibited no discernible preference for any fraction, whereas MAG_186 and 189 were more prevalent in PA10 communities. *Polaribacter* PA MAGs correlated with the diatom bloom phase, as their abundances increased amidst the diatom phase and decreased when the diatom bloom waned. During the early *Phaeocystis* bloom, MAG abundances increased from their nadir and then rapidly declined again. MAG_186 correlated well with diatom and *Phaeocystis* biovolume estimates, while MAG_189 displayed high relative abundance in a few samples. All *Polaribacter* MAGs featured PULs for α-glucan, β-glucan, and alginate, but MAG_186 (19 predicted PULs and PULs-like clusters) and MAG_189 featured further PULs including putative fucoidan PULs (Fig. S25).

*Algibacter* (1 MAG): MAG_131 represented another PA clade, whose abundance increased rapidly and peaked during the diatom bloom's collapse, and then decreased during the *Phaeocystis* bloom to 2% abundance (Fig. S23D in Additional file 2). It featured 15 predicted PULs and PULs-like clusters and 53 CAZyme genes, including two predicted PULs for α-glucan and one for laminarin, as well as abundant genes for host glycans.

'Formosa' (2 MAGs): 'Formosa' MAG_145 consistently increased during the diatom bloom's decline and peaked during the *Phaeocystis* bloom in FL and PA communities (Fig. S23E in Additional file 2). In contrast, MAG_142, represented a FL member of the same genus as the previously cultured Helgoland strain Hel1_33_131 [4]. It was abundant during and after the diatom/*Chattonella* bloom, corroborating previously detected recurrence during coastal North Sea spring phytoplankton blooms [5]. Both, MAG_142 and 145 contained similar CAZyme gene numbers (n=30-33), with MAG_142 representing the FL MAG with the highest predicted number of PULs and PULs-like clusters (n=15). Both MAGs featured PULs for α-glucan, β-glucan, host glycans and cellulose, with MAG_142 featuring additional genes to target sulfated xylans and β-mannans.

*Order* Chitinophagales

CAZyme gene numbers in the eight *Chitinophagales* MAGs varied between 17 and 44 (Fig. 7). The highest number was observed in MAG_446 (*Saprospiraceae*; JAFMDF01) with PULs predicted to target at least eleven polysaccharide substrates, including pectin (GH28, GH106, GH105), alginate, β-glucan (variant-1) and xyloglucan (GH74). The related MAG_447 featured 35 CAZyme genes, including predicted PULs for alginate (PL7), α-glucan (type I), xyloglucan and β-glucan/xylan (CBM6, GH16, GH5, GH3, GH30). A similar PUL was also identified in MAG_461 and MAG_457 (both UBA1994). MAG_449 (24 CAZymes) harbored predicted PULs for alginate, carrageenan (GH127), β-glucan (variant-1) and α-glucan (type II). MAG_452 harbored the same PUL for β- and α-glucans plus an additional predicted fructan PUL (GH32). MAG_450 featured only a single predicted PUL for arabinogalactan (GH42), and the remaining MAGs featured predicted variant-2 and variant-3 β-glucan PULs.

*Phylum* Verrucomicrobiota

The twelve selected *Verrucomicrobiota* MAGs featured null (no CAZyme annotation using dbCAN3-sub) to 44 CAZyme genes, with the highest number present in MAG_693 (*Lentimonas*), including predicted fucoidan- (Fig. S25) and pectin-targeting CAZyme-rich gene clusters. This MAG exhibited significantly higher abundance in PA than in FL communities. Its abundance increased after the diatom bloom and peaked during the *Phaeocystis* bloom (Fig. S23F in Additional file 2). In contrast, MAG_644 (UBA985; *Persicirhabdus*) possessed only five CAZyme genes, was more abundant in PA3 communities and peaked amidst the diatom bloom.

*Genus* CL500-3 (Planctomycetota)

The three selected CL500-3 MAGs (MAG_585, MAG_591, MAG_607) all exhibited the highest relative abundances in the PA3 fractions (Fig. 7), two of which represented novel Helgoland MAGs. All these MAGs featured PULs for α-glucan, β-glucan, xylan and host-glycans. MAG_591 and MAG_607 featured also PULs for chitin.

*Order* Cellvibrionales

The BD1-7 clade (3 MAGs) was abundantly represented (Fig. S7 in Additional file 2), with a dominant ASV in each of the PA communities (Fig. S23G-H in Additional file 2). Corresponding MAG_1340 and 1321 had similar CAZyme profiles with 19 and 13 genes, respectively, whereas MAG_1317 featured only three such genes. MAG_1321 and 1340 reached significantly higher abundances (13.6% and 7.0%, respectively) than MAG_1317 (0.12%).

*Order* Alteromonadales

*Colwellia* (1 MAG)*:* MAG_1214 had the highest relative abundances in the PA fractions and represented a novel Helgoland MAG. The highest relative abundance of the corresponding ASV was detected before the diatom bloom (Table S5 in Additional file 1). MAG_1214 featured PUL-like clusters for α-glucan, β-glucan, xylan, host-glycan, alginate and chitin, and was particularly rich in PUL-like clusters for alginate.

*Paraglaciecola* (2 MAGs)*:* Both of the two selected *Paraglaciecola* MAGs were proportionally more abundant in the PA fractions. They comprise the more complete (97%) MAG_1218 and the less complete MAG_1219 (67%). The former featured PULs predicted to target α-glucan, xylan, host-glycan, alginate, chitin, α-galactan, arabinogalactan, β-mannan and cellulose, but notably no PULs for β-glucan. MAG_1218 increased during the diatom bloom's decline and decreased before the diatom bloom's decline (Table S5 in Additional file 1).

*Other proteobacterial clades*

Other noteworthy MAGs included MAG_1258 (unclassified *Nitrincolaceae*; ASP10-02a), MAG_852 (UBA7985; *Stappiaceae*), and MAG_1010 (*Sulfitobacter*), which exhibited high abundances in either PA3 or PA10 communities (Fig. S23I-K in Additional file 2) but did not feature abundant CAZyme genes (Fig. 7).

**Supplementary Materials and Methods**

*Sampling for 16S/18S rRNA gene amplicon and metagenome sequencing*

In brief, samples from about 1 m depth were successively filtered through 10, 3, and 0.2 µm pore-sized polycarbonate filters (Millipore, Schwalbach, Germany) to separate the bulk of free-living (0.2-3 µm; FL) and particle-attached bacteria (3-10 µm and >10 µm; PA3 and PA10). Filters were stored at -80 °C until further use.

*16S rRNA gene amplicon sequencing and analysis*

Adapters were trimmed with cutadapt v1.15 [6]. After removing barcoded primers, the DADA2 v1.19.2 package was used to analyze the sequences [7]. Forward and reverse fastq files were filtered using *filterAndTrim* with default parameters except for *truncLen=c(220,230)* for FL and *truncLen=c(200,200)* for PA3 and PA10 sequences. Resulting files were subsequently merged using mergePairs with default parameters. The SILVA SSU v138 Ref NR database [8] was used for taxonomic assignment. Relative read abundances were calculated using Phyloseq v1.34.0 [9] in R. Good’s coverage and diversity indices (Chao1, Simpson’s, Shannon) were calculated using the vegan v2.5-7 R package [10]. One-way analysis of variance (ANOVA) and PERMANOVA were performed in Prism (GraphPad Software, Boston, MA, USA). To determine community composition differences between FL and PA bacteria, non-metric multidimensional scaling (NMDS) with Bray-Curtis dissimilarity was computed using vegan in R.

*18S rRNA gene amplicon sequencing and analysis*

The V7 region of the 18S rRNA gene was amplified using the primers F-1183mod and R-1443mod [11] coupled to custom adaptor-barcode constructs. PCR amplification and Illumina MiSeq library preparation and sequencing (V3 chemistry) were carried out by LGC Genomics (LGC Genomics, Berlin, Germany). Sequence reads free of the adaptor and primer sequences were processed using DADA2 in R. Resulting ASVs were classified using the Protist Ribosomal Reference database (PR2) [12] (v4.13, *minboot*: 50) for 18S rRNA with the RDP classifier [13] built-in DADA2. Reads classified as metazoan (zooplankton) were removed prior to downstream analysis.

*Parameters of metagenome assembly*

Parameters used in SPAdes v3.11.1 [14]: *-meta*; k-mer lengths: 21, 33, 55, 77, 99, 127; error correction enabled. Parameters used in MEGAHIT v1.2.9 [15]: kmer length: 21. Parameters used in Flye v2.9.1 [16]: *--meta* *--pacbio-hifi* *--keep-haplotypes --hifi-error* *0.01*.

Metagenome taxonomic classification

We conducted reads-based taxonomic classifications of Illumina-sequenced metagenomes of each of the three size fractions (FL: 0.2-3 µm, PA3: 3-10 µm, PA10: >10 µm) from the following eight dates: 2018/03/19, 2018/04/12, 2018/04/17, 2018/04/26, 2018/05/08, 2018/05/11, 2018/05/22, 2018/05/29. Taxonomic classification of these 24 metagenomes was done by sequential use of Kraken 2 [17], Kaiju [18], Centrifuge [19] and CLARK [20] predictions. In brief, we first ran Kraken 2 (k-mers) and then funneled the remaining unassigned reads to Kaiju (protein-coding genes). Reads that were still unassigned were then imported into Centrifuge (Burrows-Wheeler transform plus Ferragina-Manzini indexing) and ultimately into CLARK (k-mers).

*Metagenome-assembled genome (MAG) retrieval*

Illumina metagenome assemblies were binned individually within Anvi’o v6.2 using CONCOCT [21], MetaBAT2 [22] and MaxBin2 [23]. Assemblies of PacBio metagenomes were binned individually within Anvi’o v7.1 [24] using MetaBAT2 and MaxBin2. Binning results were aggregated in DAS Tool [25] to find an optimized, non-redundant set for each assembly. Bins were manually refined using *anvi-refine* and the Anvi’o interactive interface for the reconstruction of MAGs.

*MAG abundances*

For Illumina metagenomes BBMap v38.86 (https://sourceforge.net/projects/bbmap) was used to map reads to corresponding dereplicated medium and high-quality MAGs (mode: *fast, idfilter: 97, minid: 99*), and for PacBio metagenomes Minimap2 v2.24 [26] was used in an analogous manner (*map-hifi* preset). Resulting SAM files were converted to BAM files using Samtools [27]. Sequencing depth was determined using genomecov (*-bga* option) from bedtools [28]. The *BedGraph.tad.rb* script (option range 80) from the enveomics collection [29] was then used to estimate the 80% central truncated average of the sequencing depth (TAD). Sequencing depths for all microbial genomes were determined using the MicrobeCensus pipeline [30] as follows: MAG abundance = (TAD of a MAG) / (sum of sequencing depths of all microbial genomes in the corresponding metagenome).

*Annotation of predicted genes from metagenomes and MAGs*

CAZymes were predicted via the run_dbcan program of dbCAN2 [31], Diamond blastp searches against the Carbohydrate-Active Enzymes (CAZy) database (<http://www.cazy.org/>) [32] (as of July 31, 2020) and HMMER v3.3.2 [33] against Pfam HMM models [34] (as of September 2020).

Genes coding for TonB-dependent transporters (TBDTs including SusC-like proteins) were predicted by searching against the corresponding TIGRFAM and PFAM profiles using HMMER. Profiles used for the prediction of TBDTs: TIGR01352, TIGR01778, TIGR01779, TIGR01782, TIGR01783, TIGR01785, TIGR01786, TIGR02796, TIGR02797, TIGR02803, TIGR02804, TIGR02805, TIGR04056, TIGR04057, PF00593 (TonB-dependent receptor), PF07715 (TonB-dependent receptor plug domain), PF13620 (Carboxypeptidase regulatory-like domain), PF01618 (MotA/TolQ/ExbB proton channel family) and PF13715 (CarboxypepD_reg-like domain). SusD-like proteins and sulfatases were annotated by searching against corresponding Pfam profiles (PF07980, PF12741, PF12771, PF14322, PF00884) using HMMER.

*Prediction of CAZyme-rich gene clusters and PULs*

We searched for candidate genes (genes coding for PLs, GHs, CEs, sulfatases, TBDTs, or SusD-like proteins), and once such a candidate gene was found, ten genes downstream were checked for further candidates. In case an additional candidate gene was found, the window of ten genes was shifted by one gene. The process was repeated until no further candidate genes were found [35].

*Substrate class prediction of CAZymes in metagenomes and MAGs*

Target substrate classes of CAZymes were assigned using the dbCAN3-sub database [36]. PULDB [37] was also considered as a reference.

*Selection of housekeeping genes for phylogenetic analysis*

Sequences of the following ribosomal proteins and single copy genes were used for the phylogenomic analysis of MAGs: RBFA, Ribosomal_L1, Ribosomal_L17, Ribosomal_L23, Ribosomal_L3, Ribosomal_L4, Ribosomal_L5, Ribosomal_L6, Ribosomal_S11, Ribosomal_S13, Ribosomal_S15, Ribosomal_S16, Ribosomal_S17, Ribosomal_S19, Ribosomal_S2, Ribosomal_S6, Ribosomal_S7, Ribosomal_S8, Ribosomal_S9, SecE, SecG, SecY, SmpB, ADK, AICARFT_IMPCHas, ATP-synt, ATP-synt_A, EF_TS, Ribosomal_L24, RNA_pol_L, RNA_pol_Rpb6, RRF, RsfS, RuvX, tRNA_m1G_MT, tRNA-synt_1d, TsaE and YajC [24].

*Metaproteome analyses of the FL fraction – sample preparation*

Filters for metaproteome analysis were prepared as previously described [38]. One-eighth of a filter (Millipore Express PLUS Membrane, polyethersulfone, hydrophilic, 0.2 µm pore size, diameter 142 mm) was cut into approximately 10 x 10 mm fragments and transferred to 15 mL low binding tubes containing 1 mL resuspension buffer 1 (50 mM Tris-HCl (pH 7.5), 0.1 mg mL^1^ chloramphenicol, 1 mM phenylmethylsulfonyl fluoride (PMSF)). After mixing with 1.5 mL resuspension buffer 2 (20 mM Tris-HCl pH 7.5, 2% SDS (w/v)) for 10 min at 60 °C at 1,000 rpm in a thermo-mixer (Eppendorf, Wesseling-Berzdorf, Germany), 5 mL DNAse buffer (20 mM Tris-HCl pH 7.5, 0.1 mg mL^-1^ MgCl2, 1 mM PMSF, 1 μg mL^-1^ DNAse I) was added, and cells were lysed by ultra-sonication (amplitude 51-60%; cycle 0.5; 4-times 2 min) on ice. The lysate was incubated in the thermo-mixer for 10 min at 37 °C at 1,000 rpm. After centrifugation for 10 min at 4 °C at 10,000 *× g*, the supernatant (containing protein extract) was collected and the pelleted filter pieces were stirred and centrifuged again for 1 min at 4 °C at 5,000 *× g*. The supernatant was added to the previously collected supernatant. Proteins in the supernatant were precipitated by adding pre-cooled trichloroacetic acid (20% TCA (v/v)) and after inverting the tube approximately 10-times, the precipitate was pelleted by centrifugation (30 min, 4 °C, 12,000 *× g*). The protein pellet was washed 3-times in pre-cooled (‑20 °C) acetone (10 min, 4 °C, 12,000 *× g*) and dried at room temperature. The protein pellet was resuspended in 2× SDS sample loading buffer (4% SDS (w/v), 20% glycerine (w/v), 100 mM Tris-HCl pH 6.8, bromphenol blue (tip of a spatula, to add color), 3.6% 2‑mercaptoethanol (v/v) (freshly added before use)) by incubation 5 min at 95 °C, 5 min sonication bath before vortexing, and separated by 1D SDS-PAGE (Criterion TG 4-20% Precast Midi Gel, BIO-RAD Laboratories, Inc., USA). After separation, fixation, and staining with Coomassie, each gel lane was cut into 20 pieces as described previously with some modifications [39]. The gel pieces were destained 3-times for 10 min with 1 mL of gel washing buffer (200 mM ammonium bicarbonate in 30% acetonitrile (v/v)) at 37 °C under vigorous shaking. The destained gel pieces were dehydrated in 1 mL 100% acetonitrile (v/v) for 20 min before drying in a vacuum centrifuge at 30 °C. For reduction and alkylation, the gel pieces were treated with 100 µL 10 mM Dithiothreitol in 25 mM ammonium bicarbonate buffer, incubated for 1 h at 56 °C, mixed with 100 µL 55 mM iodoacetamide in 25 mM ammonium bicarbonate buffer and further incubated in the dark for 45 min at room temperature before the supernatant was removed. The gel pieces were washed with 1 mL 25 mM ammonium bicarbonate buffer (10 min, 1,000 rpm) before the supernatant was removed. Next, the gel pieces were dehydrated with 500 µL 100% acetonitrile for 10 min, and the supernatant was removed before the gel pieces were completely dried in a vacuum centrifuge (20 min) and finally covered with 120 µL trypsin solution (2 µg/mL Trypsin (Promega™). After rehydration for 20 min at room temperature, excess trypsin solution was removed with gel loader tips and incubated in a thermo-mixer for 15 h at 37 °C without shaking. Peptides were eluted with 120 µL solvent A (water MS grade in 0,1% acetic acid (v/v)) by sonication for 15 min. The supernatant was transferred into a new tube. Peptides were eluted again with 120 µL 30% acetonitrile (v/v) by sonication for 15 min. The supernatant was transferred into the same new tube as used before. The sample volume was reduced in a vacuum centrifuge to a maximum of 15 to 20 µL. The peptides were desalted via ZipTips µC18 (Merck Millipore, P10 tip size) according to the manufacturer’s protocol. The eluted samples were dried in a vacuum centrifuge and resuspended in 10 µL 0.5x Biognosys™ iRT standard kit in solvent A.

*Metaproteome analyses of the FL fraction - LC-MS/MS measurement and data analysis*

For measurement, an Easy-nLC1000 (Thermo Fisher Scientific, Waltham, MA, USA) was coupled to an Q Exactive mass spectrometer (Thermo Fisher Scientific). Samples were loaded onto in-house packed capillary columns of 20 cm length and 75 µm inner diameter. Columns were filled with Dr. Maisch ReproSil Pur 120 C18-AQ 1.9 µm (Dr. Maisch GmbH, Ammerbuch-Entringen, Germany). Peptides were separated using a 131 min nonlinear binary gradient from 2% to 99% solvent B (99.9% acetonitrile(v/v), 0.1% acetic acid (v/v)) in solvent A at a constant flow rate of 300 nL min^-1^. The MS1 scan was recorded in the orbitrap with a mass window of 300–1,650 m/z and a resolution of 140,000 at 200 m/z. The 15 most intense precursor ions (ions with an unassigned charge or a charge of 1,7,8, >8 are excluded) were selected for HCD fragmentation with a normalized collision energy of NCE 27. The resulting MS/MS spectra were acquired were recorded with a resolution of 17.500 at 200 m/z. Dynamic exclusion and lock mass correction were enabled.

All MS/MS spectra were analyzed using Mascot (version 2.7.0.1; Matrix Science, London, UK). Mascot was set up to search the database containing all protein sequences from the 18 metagenomes obtained during the spring bloom of 2018, assuming the digestion enzyme trypsin. For database construction, redundant proteins from the 18 metagenomic samples were removed using cd-hit [40] with a clustering threshold of 97% identity. The created database was added by a set of common laboratory contaminants and reverse entries, amounting to 81,874,922 sequences in the final database.

The database search with Mascot [41] was performed with the following parameters: fragment ion mass tolerance and parent ion tolerance of 10 ppm, none missed cleavages, methionine oxidation as a variable modification, and cysteine carbamidomethylation as fixed modification. Scaffold (version 4.11.1; Proteome Software Inc., Portland, OR) was used to merge the search results and to validate MS/MS based peptide and protein identifications. During creation of the Scaffold file, an additional X! Tandem search was performed for validation (version 2017.2.1.4; The GPM, thegpm.org; version X!Tandem Alanine) with default settings (fragment ion mass tolerance and parent ion tolerance of 10 ppm, carbamidomethyl of cysteine as fixed modification, Glu->pyro-Glu of the n-terminus, ammonia-loss of the n-terminus, Gln->pyro-Glu of the n-terminus, oxidation of methionine and carbamidomethyl of cysteine as variable modifications). Peptide identifications were accepted if they could be established at greater than 95% probability by the PeptideProphet algorithm [42] with Scaffold delta-mass correction. Protein identifications were accepted if they could be established at greater than 99% probability and contained at least 2 identified peptides. Protein probabilities were assigned by the ProteinProphet algorithm [43]. Proteins that contained similar peptides and could not be differentiated based on MS/MS analysis alone were grouped to satisfy the principles of parsimony.

For (semi-)quantitative analysis, the Scaffold’s ‘Quantitative Value’ for normalized, weighted spectra for each protein group was divided by the sum of all quantitative values for the sample to calculate the percent normalized weighted spectra (%NWS). Average values were calculated from all three biological replicates of each sample. Protein groups that were not identified within a replicate were included as ‘0’ in this calculation. In order to make Bacteria-specific %NWS readily comparable across all samples, all bacterial spectra were normalized to 100% (%BacNWS).

*Assignment of expressed proteins to MAGs for FL metaproteomes*

To assign identified expressed protein groups to MAGs, amino acid sequences were aligned to all predicted proteins of all MAGs from the FL fraction 2018 using BLAST v2.11.0 [44]. Alignments with identities >99%, e-values below E-4 and coverages of at least 50% for both the query and the subject were considered correctly assigned.

*Metaproteome analyses of the PA fractions – sample preparation*

Sample preparation for the PA fractions has been described earlier for this dataset [45, 46]. In brief, sequential filtration was performed for three selected time points with polycarbonate membrane filters (142 mm diameter, Millipore) of 3 µm and 10 µm pore sizes. Proteins were extracted using the bead-beating protocol described in [45], separated by 1D SDS-PAGE and split into 20 fractions per sample. After washing and in-gel-trypsin digestion, peptides were desalted using ZipTips µC18 (Merck Millipore, P10 tip size) as described for the FL fraction and dried by vacuum centrifugation.

*Metaproteome analyses of the PA fractions - LC-MS/MS measurement and data analysis*

LC-MS / MS measurement for the PA fractions was performed in triplicates using an Orbitrap VelosTM mass spectrometer (ThermoFisher Scientific, Waltham, MA, USA). 2018 MAGs were included into the database for analysis. For this, 2018 MAGs were added to the metagenome-based database described in [46]. Redundant sequences were removed with cd-hit [40] with a clustering threshold of 97% identity before adding the 2018 MAGs to ensure that all 2018 MAG sequence information would be kept for downstream analysis. The final database used for analysis of the PA metaproteome fractions contained 15,529,863 entries.

To allow for comparison between the 3-10 µm and the >10 µm metaproteome fractions, samples for both filter sizes and from all three-time points were merged into one dataset in Scaffold. If protein groups contained more than one protein, 2018 MAG sequences were prioritized as representative proteins of that protein group. As described for the FL fraction, (semi-)quantitative values were calculated as the average of three technical replicates for each sample based on the percentage of the normalized weighted spectra value provided by the Scaffold. Protein groups that were not identified in a replicate were included with a value of ‘0’ in this calculation.

**Code Availability**

Softwares/bioinformatic tools used in this study:

*Anvio 6.2 and Anvio 7.1*: https://github.com/merenlab/anvio;

*Barrnap*: https://github.com/tseemann/barrnap;

*BBMap:* https://jgi.doe.gov/data-and-tools/software-tools/bbtools/bb-tools-user-guide/bbmap-guide/;

*BLAST*: <ftp://ftp.ncbi.nlm.nih.gov/blast/executables/blast+/LATEST>;

*Centrifuge*: https://github.com/centrifugal/centrifuge;

*CheckM*: <https://ecogenomics.github.io/CheckM>;

*CLARK:* [*http://clark.cs.ucr.edu*](http://clark.cs.ucr.edu);

*CONCOCT*: <https://github.com/BinPro/CONCOCT>;

*Cutadapt*: https://github.com/marcelm/cutadapt;

*DAS Tool:* https://github.com/cmks/DAS_Tool;

*dbCAN2*: <http://bcb.unl.edu/dbCAN2/index.php>;

*DADA2*: <https://github.com/benjjneb/dada2>;

*dRep*: <https://drep.readthedocs.io/en/latest/>;

*DIAMOND*: <http://www.diamondsearch.org/index.php>;

*FastANI*: <https://github.com/ParBLiSS/FastANI>;

*FastQC*: <http://www.bioinformatics.babraham.ac.uk/projects/fastqc>;

*FastTree*: <http://www.microbesonline.org/fasttree>;

*Flye*: https://github.com/fenderglass/Flye;

*FragGeneScan:* https://github.com/gaberoo/FragGeneScan;

*GTDB-Tk*: <https://github.com/Ecogenomics/GTDBTk>;

*HMMER*: <http://hmmer.org>;

*iTOL v6.5.6*: https://github.com/iBiology/iTOL;

*Kaiju:* <https://github.com/bioinformatics-centre/kaiju>;

*Karen2*: <https://github.com/DerrickWood/kraken2>;

*MAFFT*: <https://mafft.cbrc.jp/alignment/software>;

*MaxBin2*: <https://sourceforge.net/projects/maxbin2>;

*MEGAHIT*: <https://github.com/voutcn/megahit>;

*MetaBAT2*: <https://bitbucket.org/berkeleylab/metabat>;

*MicrobeCensus:* <https://github.com/snayfach/MicrobeCensus>;

*Minimap2:* <https://github.com/lh3/minimap2>;

*Prodigal*: <https://github.com/hyattpd/Prodigal>;

*Prokka*: <https://github.com/tseemann/prokka>;

*QUAST:* https://github.com/ablab/quast;

*R*: <https://www.r-project.org>;

*RDP classifier:* https://github.com/rdpstaff/classifier;

*SAMTools*: <http://www.htslib.org>;

*Silvangs:* https://ngs.arb-silva.de/silvangs/#;

*Simka:* <https://github.com/GATB/simka>;

*SPAdes*: https://github.com/ablab/spades;

*SqueezeMeta*: https://github.com/jtamames/SqueezeMeta;

**Database Availability**

Database used in this study:

*Cazy*: <http://www.cazy.org>;

*dbCAN-sub*: https://bcb.unl.edu/dbCAN_sub/;

*Pfam*: http://pfam.xfam.org;

*Protist Ribosomal Reference database (PR2)*: <https://pr2-database.org>;

*SILVA SSU v138 Ref NR database*: <https://www.arb-silva.de/documentation/release-138/>;

*TIGRFAM*: <https://www.jcvi.org/research/tigrfams>;

**References**

1. McMurdie PJ, Holmes S. Waste not, want not: Why rarefying microbiome data is inadmissible. PLoS Comput Biol. 2014;10(4):e1003531.

2. Hong J, Karaoz U, de Valpine P, Fithian W. To rarefy or not to rarefy: robustness and efficiency trade-offs of rarefying microbiome data. Bioinformatics. 2022;38(9):2389-96.

3. Rodriguez-Gijon A, Nuy JK, Mehrshad M, Buck M, Schulz F, Woyke T, et al. A genomic perspective across Earth's microbiomes reveals that genome size in Archaea and Bacteria is linked to ecosystem type and trophic strategy. Front Microbiol. 2022;12:761869.

4. Hahnke RL, Bennke CM, Fuchs BM, Mann AJ, Rhiel E, Teeling H, et al. Dilution cultivation of marine heterotrophic bacteria abundant after a spring phytoplankton bloom in the North Sea. Environ Microbiol. 2015;17(10):3515-26.

5. Teeling H, Fuchs BM, Bennke CM, Krüger K, Chafee M, Kappelmann L, et al. Recurring patterns in bacterioplankton dynamics during coastal spring algae blooms. eLife. 2016;5:e11888.

6. Martin M. Cutadapt removes adapter sequences from high-throughput sequencing reads. EMBnet journal. 2011;17(1):10-2.

7. Callahan BJ, McMurdie PJ, Rosen MJ, Han AW, Johnson AJA, Holmes SP. DADA2: high-resolution sample inference from Illumina amplicon data. Nat Methods. 2016;13(7):581-3.

8. Quast C, Pruesse E, Yilmaz P, Gerken J, Schweer T, Yarza P, et al. The SILVA ribosomal RNA gene database project: improved data processing and web-based tools. Nucleic Acids Res. 2013;41(Database issue):D590-6.

9. McMurdie PJ, Holmes S. phyloseq: an R package for reproducible interactive analysis and graphics of microbiome census data. Plos One. 2013;8(4):e61217.

10. Dixon P. VEGAN, a package of R functions for community ecology. J Veg Sci. 2003;14(6):927-30.

11. Ray JL, Althammer J, Skaar KS, Simonelli P, Larsen A, Stoecker D, et al. Metabarcoding and metabolome analyses of copepod grazing reveal feeding preference and linkage to metabolite classes in dynamic microbial plankton communities. Mol Ecol. 2016;25(21):5585-602.

12. Guillou L, Bachar D, Audic S, Bass D, Berney C, Bittner L, et al. The Protist Ribosomal Reference database (PR2): a catalog of unicellular eukaryote small sub-unit rRNA sequences with curated taxonomy. Nucleic Acids Res. 2013;41(Database issue):D597-604.

13. Wang Q, Garrity GM, Tiedje JM, Cole JR. Naïve Bayesian classifier for rapid assignment of rRNA sequences into the new bacterial taxonomy. Applied and environmental microbiology. 2007;73(16):5261-7.

14. Bankevich A, Nurk S, Antipov D, Gurevich AA, Dvorkin M, Kulikov AS, et al. SPAdes: a new genome assembly algorithm and its applications to single-cell sequencing. J Comput Biol. 2012;19(5):455-77.

15. Li DH, Luo RB, Liu CM, Leung CM, Ting HF, Sadakane K, et al. MEGAHIT v1.0: a fast and scalable metagenome assembler driven by advanced methodologies and community practices. Methods. 2016;102:3-11.

16. Kolmogorov M, Bickhart DM, Behsaz B, Gurevich A, Rayko M, Shin SB, et al. metaFlye: scalable long-read metagenome assembly using repeat graphs. Nat Methods. 2020;17(11):1103-10.

17. Wood DE, Salzberg SL. Kraken: ultrafast metagenomic sequence classification using exact alignments. Genome Biol. 2014;15(3):R46.

18. Menzel P, Ng KL, Krogh A. Fast and sensitive taxonomic classification for metagenomics with Kaiju. Nat Commun. 2016;7(1):11257.

19. Kim D, Song L, Breitwieser FP, Salzberg SL. Centrifuge: rapid and sensitive classification of metagenomic sequences. Genome Res. 2016;26(12):1721-9.

20. Ounit R, Wanamaker S, Close TJ, Lonardi S. CLARK: fast and accurate classification of metagenomic and genomic sequences using discriminative k-mers. Bmc Genomics. 2015;16(1):236.

21. Alneberg J, Bjarnason BS, de Bruijn I, Schirmer M, Quick J, Ijaz UZ, et al. Binning metagenomic contigs by coverage and composition. Nat Methods. 2014;11(11):1144-6.

22. Kang DWD, Li F, Kirton E, Thomas A, Egan R, An H, et al. MetaBAT 2: an adaptive binning algorithm for robust and efficient genome reconstruction from metagenome assemblies. Peerj. 2019;7:e7359.

23. Wu YW, Simmons BA, Singer SW. MaxBin 2.0: an automated binning algorithm to recover genomes from multiple metagenomic datasets. Bioinformatics. 2016;32(4):605-7.

24. Eren AM, Esen OC, Quince C, Vineis JH, Morrison HG, Sogin ML, et al. Anvi'o: an advanced analysis and visualization platform for 'omics data. PeerJ. 2015;3:e1319.

25. Sieber CMK, Probst AJ, Sharrar A, Thomas BC, Hess M, Tringe SG, et al. Recovery of genomes from metagenomes via a dereplication, aggregation and scoring strategy. Nat Microbiol. 2018;3(7):836-43.

26. Li H. Minimap2: pairwise alignment for nucleotide sequences. Bioinformatics. 2018;34(18):3094-100.

27. Danecek P, Bonfield JK, Liddle J, Marshall J, Ohan V, Pollard MO, et al. Twelve years of SAMtools and BCFtools. Gigascience. 2021;10(2):giab008.

28. Quinlan AR, Hall IM. BEDTools: a flexible suite of utilities for comparing genomic features. Bioinformatics. 2010;26(6):841-2.

29. Rodriguez-R LM, Konstantinidis KT. The enveomics collection: a toolbox for specialized analyses of microbial genomes and metagenomes. PeerJ Preprints. 2016;4:e1900v1.

30. Nayfach S, Pollard KS. Average genome size estimation improves comparative metagenomics and sheds light on the functional ecology of the human microbiome. Genome Biol. 2015;16(1):51.

31. Zhang H, Yohe T, Huang L, Entwistle S, Wu P, Yang Z, et al. dbCAN2: a meta server for automated carbohydrate-active enzyme annotation. Nucleic Acids Res. 2018;46(W1):W95-W101.

32. Drula E, Garron ML, Dogan S, Lombard V, Henrissat B, Terrapon N. The carbohydrate-active enzyme database: functions and literature. Nucleic Acids Res. 2022;50(D1):D571-D7.

33. Eddy SR. A new generation of homology search tools based on probabilistic inference. Genome Inform. 2009;23(1):205-11.

34. Mistry J, Chuguransky S, Williams L, Qureshi M, Salazar GA, Sonnhammer ELL, et al. Pfam: The protein families database in 2021. Nucleic Acids Res. 2021;49(D1):D412-D9.

35. Lu D, Wang F, Amann RI, Teeling H, Du JZ. Epiphytic common core bacteria in the microbiomes of co-located green (*Ulva*), brown (*Saccharina*) and red (*Grateloupia*, *Gelidium*) macroalgae. Microbiome. 2023;11(1):126.

36. Zheng J, Ge Q, Yan Y, Zhang X, Huang L, Yin Y. dbCAN3: automated carbohydrate-active enzyme and substrate annotation. Nucleic Acids Res. 2023;51(W1):W115-W21.

37. Terrapon N, Lombard V, Drula E, Lapebie P, Al-Masaudi S, Gilbert HJ, et al. PULDB: the expanded database of Polysaccharide Utilization Loci. Nucleic Acids Res. 2018;46(D1):D677-D83.

38. Deusch S, Seifert J. Catching the tip of the iceberg - evaluation of sample preparation protocols for metaproteomic studies of the rumen microbiota. Proteomics. 2015;15(20):3590-5.

39. Bonn F, Bartel J, Büttner K, Hecker M, Otto A, Becher D. Picking vanished proteins from the void: how to collect and ship/share extremely dilute proteins in a reproducible and highly efficient manner. Anal Chem. 2014;86(15):7421-7.

40. Li W, Godzik A. Cd-hit: a fast program for clustering and comparing large sets of protein or nucleotide sequences. Bioinformatics. 2006;22(13):1658-9.

41. Perkins DN, Pappin DJ, Creasy DM, Cottrell JS. Probability-based protein identification by searching sequence databases using mass spectrometry data. Electrophoresis. 1999;20(18):3551-67.

42. Keller A, Nesvizhskii AI, Kolker E, Aebersold R. Empirical statistical model to estimate the accuracy of peptide identifications made by MS/MS and database search. Anal Chem. 2002;74(20):5383-92.

43. Nesvizhskii AI, Keller A, Kolker E, Aebersold R. A statistical model for identifying proteins by tandem mass spectrometry. Anal Chem. 2003;75(17):4646-58.

44. Altschul SF, Gish W, Miller W, Myers EW, Lipman DJ. Basic local alignment search tool. J Mol Biol. 1990;215(3):403-10.

45. Schultz D, Zuhlke D, Bernhardt J, Francis TB, Albrecht D, Hirschfeld C, et al. An optimized metaproteomics protocol for a holistic taxonomic and functional characterization of microbial communities from marine particles. Environ Microbiol Rep. 2020;12(4):367-76.

46. Schultz D. Mechanisms of polysaccharide degradation of particle-associated microbial communities. PhD thesis, University of Greifswald. 2022.
